# Efficient Construction of Symmetrical Diaryl Sulfides via a Supported Pd Nanocatalyst-Catalyzed C-S Coupling Reaction

**DOI:** 10.3390/ijms232315360

**Published:** 2022-12-06

**Authors:** Hao Jin, Penghao Liu, Qiaoqiao Teng, Yuxiang Wang, Qi Meng, Chao Qian

**Affiliations:** 1 Jiangsu Key Laboratory of Advanced Catalytic Materials and Technology, School of Petrochemical Engineering, Changzhou University, Changzhou 213164, China; 2 Zhejiang Provincial Key Laboratory of Advanced Chemical Engineering Manufacture Technology, College of Chemical and Biological Engineering, Zhejiang University, Hangzhou 310027, China; 3 Institute of Zhejiang University—Quzhou, Quzhou 324000, China

**Keywords:** C-S coupling, DIPEA, Na_2_S_2_O_3_, nanocatalyst, Pd@COF-TB

## Abstract

Aryl sulfides play an important role in pharmaceuticals, biologically active molecules and polymeric materials. Herein, a general and efficient protocol for Pd@COF-TB (a kind of Pd nanocatalyst supported by a covalent organic framework)/DIPEA-catalyzed one-pot synthesis of symmetrical diaryl sulfides through a C-S coupling reaction from aryl iodides and Na_2_S_2_O_3_ is developed. More importantly, the addition of N,N-diisopropylethylamine (DIPEA) can not only enhance the catalytic activity of a Pd@COF-TB nanocatalyst, but also effectively inhibit the formation of biphenyl byproducts, which are a product of Ullmann reaction. Besides, it has been confirmed that the aryl Bunte salts generated in situ from Na_2_S_2_O_3_ and aryl iodides are the sulfur sources involved in this C-S coupling reaction. With the strategy proposed in this work, a variety of symmetrical diaryl sulfides could be obtained in moderate to excellent yields with a high tolerance of various functional groups. Moreover, a possible mechanism of this Pd nanoparticle-catalyzed C-S coupling reaction is proposed based on the results of controlling experiments.

## 1. Introduction

**Figure 1:**
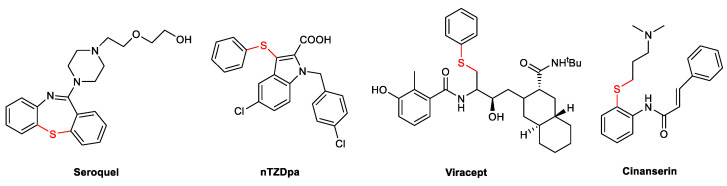
Some pharmaceutical active substances containing an aryl sulfide structure.

**Scheme 1:**
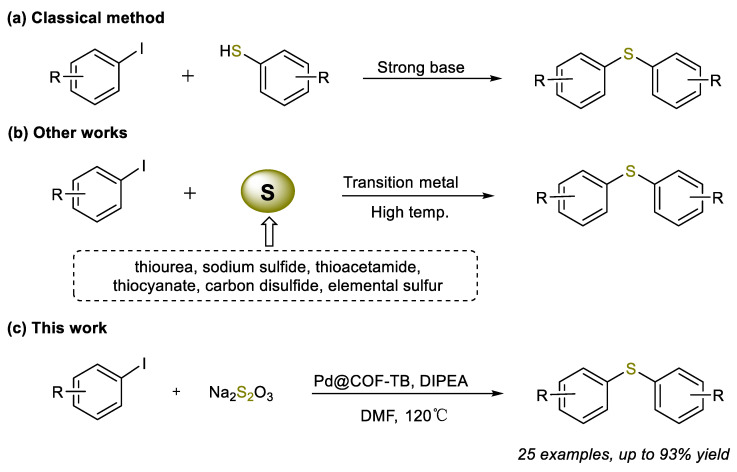
Approaches for the synthesis of symmetrical diaryl sulfides.

Sulfur-containing organic compounds have always received considerable attention due to their various functions in biology, chemistry and materials science [1,2,3]. As an important class of organic sulfur-containing structures, aryl sulfides are of great significance to the pharmaceutical industry and are a common functionality found in numerous drugs in therapeutic areas [4,5,6]. As shown in Figure 1, Seroquel is a potential antipsychotic agent, and its propensity to produce tardive dyskinesia with chronic administration to humans is markedly less than that of typical antipsychotic agents [7]. nTZDpa is an effective antibiotic against bacterial persistence [8]. Viracept is often used as an anti-human immunodeficiency virus drug together with other drugs [9]. Cinanserin exhibits good efficacy against SARS viruses and the treatment of schizophrenia [10]. In addition, sulfides are also widely used in organic synthesis. For example, Zhao and co-workers developed a new type of chiral organothioether bifunctional catalyst based on an indene skeleton, which could be effectively applied in the asymmetric trifluoromethylthioesterification of alkenoic acid [11]. Shi’s group reported a Pd(OAc)_2_-catalyzed method for the alkenylation of C-H bonds of asymmetric olefins. Here, sulfide was used as a guiding group, which could not only achieve excellent yield, enantioselectivity and stereoselectivity, but also synthesize a class of thiene ligands with a chiral axis structure [12]. Therefore, various synthetic strategies have been developed to synthesize sulfides. The most classical construction method of sulfides is the substitution reaction of halide and thiol through C-S coupling in the presence of a strong base (Scheme 1a) [13,14,15,16,17]. However, thiols are highly toxic substances with unpleasant odors. Therefore, the use of green sulfur sources to develop novel, practical and efficient methods for the construction of sulfides is demanding. After years of research, a variety of green sulfur sources, including thiourea [18,19], sodium sulfide [20,21], thioacetamide [22], thiocyanate [23,24], carbon disulfide [25] and elemental sulfur [26,27] have been successfully used in the synthesis of symmetrical diaryl sulfides via transition metal-catalyzed C-S coupling reactions.

It is well known that sodium thiosulfate (Na_2_S_2_O_3_) has been widely used in many C-S coupling reactions due to its low price and odorless and tasteless properties. Na_2_S_2_O_3_ mainly transfers the sulfur atom into organic compounds by forming Bunte salts to participate in reactions. Compared with the sulfur atom in thiols, the SO_3_Na group attached to the sulfur atom in Bunte salts would change its properties including electronic effects, steric hindrance, and resonance stability [28]. Therefore, Bunte salts are not only used as a substitute for thiols, but also could mediate some reactions that thiols cannot. In recent years, influenced by the rapid development of organosulfur chemistry, Bunte salts, formed in situ by Na_2_S_2_O_3_ and halides, are widely used as an efficient sulfur source in various C-S coupling reactions. For example, Yi et al. reported the Pd-catalyzed C-S coupling reaction of aryl halide/aryl triflate and Na_2_S_2_O_3_ to synthesize aromatic thiols [29]. In 2013, Jiang’s group reported the Pd-catalyzed one-step synthesis of sulfur-containing heterocyclic compounds through the construction of two intramolecular C-S bonds with Na_2_S_2_O_3_ as sulfur source [30]. Subsequently, the group extended the system to an intermolecular C-S coupling reaction. Asymmetric sulfides could also be synthesized by the intermolecular C-S coupling reaction of two different halides with Na_2_S_2_O_3_ under palladium catalysis [31]. In 2016, Abbasi and co-workers realized the synthesis of symmetric dialkyl disulfides from alkyl halides and Na_2_S_2_O_3_ using DMSO as a solvent [32]. In 2019, Li et al. reported a three-component tandem synthesis of isoxazole with Na_2_S_2_O_3_ catalyzed by palladium [33]. These examples also suggest that palladium nanoparticles play an important role in catalyzing C-S coupling reactions.

As a continuous work on the application of the Pd@COF-TB nanocatalyst, we herein report a novel, efficient method for the synthesis of symmetrical diaryl sulfides via a Pd@COF-TB/DIPEA-catalyzed C-S coupling reaction from aryl iodides and Na_2_S_2_O_3_ (Scheme 1c). A variety of symmetrical diaryl sulfides could be obtained in moderate to excellent yields through this protocol. In this work, DIPEA, apart from providing an alkaline environment, also acts as a palladium ligand, which could not only enhance the catalytic activity of the Pd@COF-TB nanocatalyst, but also effectively inhibit the occurrence of the Ullmann reaction. Furthermore, the reaction mechanisms and the catalytic performance of Pd@COF-TB/DIPEA are discussed.

## 2. Results and Discussion

### 2.1. Preparation of Pd@COF-TB Nanocatalyst

**Figure 2:**
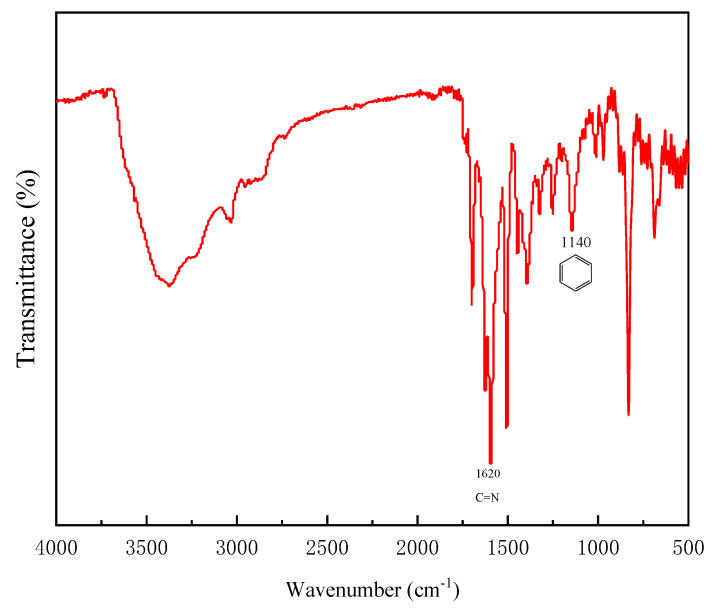
FT-IR spectra of the Pd@COF-TB nanocatalyst.

The preparation of the Pd@COF-TB nanocatalyst was carried out according to the method established in our previous work [34] and its characterization also has been reported in detail. Therefore, the newly prepared Pd@COF-TB nanocatalyst here was only characterized by Fourier transform infrared spectroscopy (FT-IR) to confirm its correct structure. As shown in Figure 2, the FT-IR absorption spectrum of the newly prepared Pd@COF-TB nanocatalyst was identical to the nanocatalyst prepared in our previous work [34], which means they have the same structure.

### 2.2. Determination of Pd Loading in Pd@COF-TB Nanocatalyst

To further determine the load of Pd in the nanocatalyst, Pd@COF-TB (4 mg) was dissolved in nitric acid (10 mL) and diluted 100 times after complete dissolution. As shown in Table 1, the content of palladium in the solution was obtained by inductively coupled plasma optical emission spectrometry (ICP-OES). The calculation showed that the palladium loading in the nanocatalyst is 4.7% wt.

### 2.3. Optimization of Reaction Conditions

It is well known that aryl halides easily undergo Ullmann reactions to form biaryl compounds (**2a**) under transition metal catalysis [35,36,37,38]. Therefore, our initial efforts focused on suppressing the occurrence of the Ullmann reaction in the C-S coupling reaction of aryl iodides with Na_2_S_2_O_3_. In order to establish standard reaction conditions, a model reaction between iodobenzene and Na_2_S_2_O_3_ to produce diphenyl sulfide (**1a**) was chosen and the effects of different reaction parameters like the solvent, base, temperature and the amount of catalyst were studied. The detailed results are summarized in Table 2. The solvent is understood to impact the reaction rate and thereby a number of solvents like DMF, DMSO, NMP, H_2_O, EtOH and PEG_200_ were tested in this model reaction. DMF was found to be best for the synthesis of diphenyl sulfide (Table 2, entry 1). Further, we explored the effect of the base on this reaction (Table 2, Entries 1, 7–13). We found that nitrogen-containing organic bases are significantly better than inorganic bases. The most effective of the bases used was observed to be DIPEA (Table 2, entry 13). Besides, the effect of the catalyst loading on this reaction was investigated (Table 2, Entries 13–18). The results showed that no product was detected without Pd@COF-TB (Table 2, entry 14), which means the Pd@COF-TB nanocatalyst could indeed catalyze the C-S coupling reaction of iodobenzene with Na_2_S_2_O_3_. Further, 20 mg was found to be the optimized in this reaction condition for the reaction to carry out (Table 2, entry 13). Besides, when Pd(OAc)_2_ with the same content of Pd replaced Pd@COF-TB, the yield and selectivity of the desired product **1a** both decreased (Table 2, entry 19), which indicated that COF-TB support could optimize the distribution of Pd nanoparticles and improve their catalytic activity. It was noticed that an increasing temperature was beneficial to the desired product **1a**. However, an excessively high reaction temperature would promote the decomposition of more DMF and generate other byproducts, so the optimal reaction temperature is 120 °C. On the basis of the results, the optimized conditions turned out to be: iodobenzene (0.2 g, 1.0 mmol), Na_2_S_2_O_3_ (0.32 g, 2.0 mmol), Pd@COF-TB (20 mg) and DIPEA (0.26 g, 2.0 mmol) in DMF (3 mL) at 120 °C.

### 2.4. Substrate Expansion under Optimal Reaction Conditions

To assess the substrate scope of this C-S coupling reaction catalyzed by Pd@COF-TB/DIPEA, we screened a range of commercially available aryl iodides with Na_2_S_2_O_3_ under the optimized conditions. The results are listed in Table 3. Unsubstituted iodobenzene exhibited an excellent reaction result, giving a 93% yield of the desired product (**1a**) under the optimized reaction conditions. Substituted iodobenzenes with an electron-rich group, such as methoxy, methyl, *tert*-butyl, hydroxyl and amino, could provide the corresponding coupling products in good to excellent yields (**1b–k**). Due to the influence of the steric hindrance of the substituents, the reaction result of *para*-substituted iodobenzene is the best, followed by *meta*-substitution, and *ortho*-substitution is the worst (**1b–g**). Besides, electron-deficient iodobenzenes bearing fluoro, chloro, bromo, cyano, trifluoromethyl, nitro group could give the desired products in moderate to good yields. It is worth mentioning that the reactions of these substituted iodobenzenes with an electron-poor group would produce more biaryl byproducts. Furthermore, multi-substituted iodobenzene could also react smoothly and give the corresponding products in good yields through this protocol (**1r, 1s**). Additionally, this Pd@COF-TB/DIPEA-catalyzed strategy could also be applied to heteroaromatic iodides, such as naphthalene (**1t**), thiophene (**1u, 1v**) and pyridine (**1w–1t**). These heterocyclic substrates could generate the desired coupling products in good to excellent yields under the optimized conditions without being affected by the position of iodine. These results show the excellent substrate compatibility and functional group tolerance of this protocol.

### 2.5. Gram-Scale Synthesis Reaction of Iodobenzene with Na_2_S_2_O_3_

**Scheme 2:**
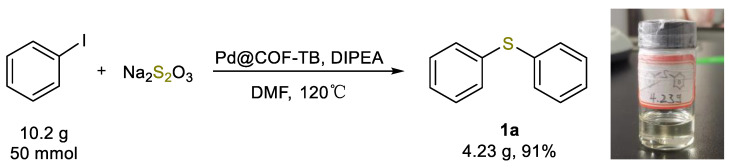
Gram-scale synthesis reaction of iodobenzene with Na_2_S_2_O_3_.

We investigated the conversion of iodobenzene as a substrate to diphenyl sulfide (**1a**) by the above method in a scale-up reaction (Scheme 2). It was shown that when iodobenzene was 50 mmol, the desired product (**1a**) could be still obtained with a high yield of 91% and good selectivity of 94%.

### 2.6. Catalyst Reuse

**Figure 3:**
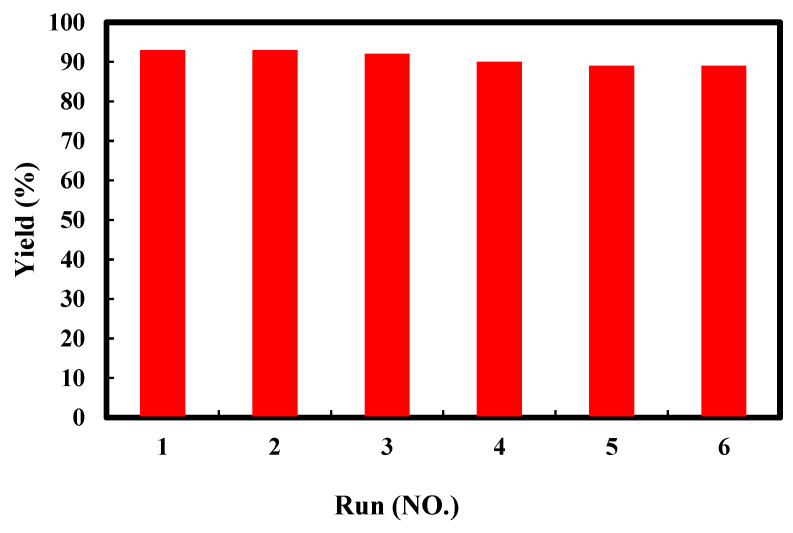
Recovery and reuse of the Pd@COF-TB nanocatalyst.

In order to test the reusability of the Pd@COF-TB nanocatalyst, cycling reuse tests were performed for the reaction of iodobenzene with Na_2_S_2_O_3_ under the optimized reaction conditions in this study. After each run, the nanocatalyst was separated by filtration, washed, dried under vacuum, and then carried out for the next consecutive cycles. As shown in Figure 3, the Pd@COF-TB nanocatalyst could be efficiently recycled and reused for six cycles without a significant decrease in the desired product (**1a**) yield, which means the catalyst has receptable reusability.

### 2.7. Mechanism Studies

**Scheme 3:**
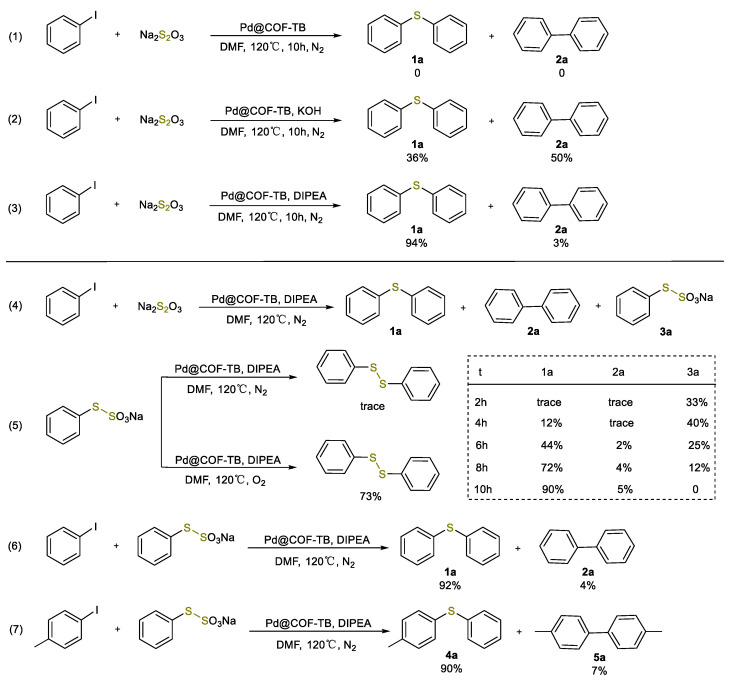
Controlling experiments. Typical conditions: iodobenzene/4-iodotoluene/sodium S-phenyl sulfurothioate (1.0 mmol, 0.5 mmol in Equations (6) and (7)), Na_2_S_2_O_3_ (2.0 mmol), Pd@COF-TB (20 mg), and DIPEA (2 mmol) in 3 mL DMF, 120 °C, in N_2_. Yields in Equations (1)–(3) were determined by GC-MS and yields in Equations (4)–(7) were determined by LC-MS.

In order to gain mechanistic insight into this protocol, some controlling experiments were carried out (Scheme 3). As shown in Equation (1), no product was detected in the absence of a base, suggesting that the existence of a base is crucial. After the addition of KOH, the product of the Ullmann reaction (Equation (2), **2a**) was significantly more than the desired C-S coupling product (Equation (2), **1a**). However, the yield and selectivity of diphenyl sulfide were significantly improved when KOH was replaced by DIPEA (Equation (3)). These results show that DIPEA, apart from providing an alkaline environment, is also an excellent ligand for transition metals [39,40,41], which could enhance the catalytic activity of a Pd@COF-TB nanocatalyst. At the same time, due to the steric hindrance effect, DIPEA could significantly inhibit the occurrence of the Ullmann reaction. Furthermore, we monitored the progress of the C-S coupling reaction of iodobenzene with Na_2_S_2_O_3_ (Equation (4)) using LC-MS. The results showed that sodium S-phenyl sulfurothioate (**3a**) was formed in situ from iodobenzene and Na_2_S_2_O_3_ in this reaction. The yield of this phenyl Bunte salt showed a trend of increasing first and then decreasing, which means that the Bunte salt was mainly produced in the early stage and then converted into the desired product. In order to verify whether the Bunte salt is the intermediate, sodium S-phenyl sulfurothioate reacted alone under standard conditions. As a result, a trace amount of diphenyl disulfide was found without diphenyl sulfide generated. The yield of diphenyl disulfide was greatly improved when the reaction was exposed to O_2_ (Equation (5)). These outcomes suggest that the Bunte salt is a component involved in the C-S coupling reaction for the synthesis of diphenyl sulfide. The reaction must be carried out under anaerobic conditions in order to inhibit the formation of diphenyl disulfide. Finally, treating iodobenzene with sodium S-phenyl sulfurothioate under standard conditions predominately afforded diphenyl sulfide in a 92% yield, along with biphenyl in a 4% yield (Equation (6)). Furtherly, 4-methyldiphenyl sulfide (**4a**) could be obtained through the reaction of 4-iodotoluene and sodium S-phenyl sulfurothioate under standard conditions (Equation (7)). These results indicated that the Bunte salts formed by aryl iodides and Na_2_S_2_O_3_ were the real sulfur sources participating in this C-S coupling reaction.

**Scheme 4:**
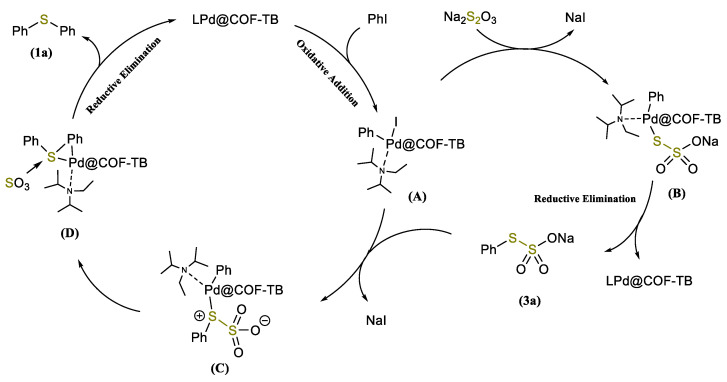
Proposed mechanism.

Based on the above-mentioned experimental results and combined with the previously similar reports [29,31,42,43], a proposed reaction mechanism for the catalytic process of Pd@COF-TB/DIPEA is proposed in Scheme 4. Firstly, a catalytically active Pd^(0)^ species generated in situ from Pd@COF-TB nanoparticles is coordinated with DIPEA to form LPd@COF-TB. Then, organopalladium intermediates (**A**) are generated via the oxidative addition of LPd@COF-TB with iodobenzene. A part of **A** reacts with Na_2_S_2_O_3_ to obtain organopalladium intermediate **B** by eliminating NaI. Through reductive elimination step, sodium S-phenyl sulfurothioate **3a** is obtained from **B**. Subsequently, another part of intermediate **A** reacts with Bunte salt **3a**, producing aryl palladium thiosulfate intermediate **C**, which could be transformed to intermediate **D** via the release of SO_3_. Finally, through a second reductive elimination, intermediate **D** gave the desired product diphenyl sulfide **1a** and regenerated LPd@COF-TB. It should be noted that the LPd@COF-TB generated in the two reductive elimination processes would continue to undergo oxidative addition reaction with unreacted iodobenzene again. In addition, we speculated that due to the steric hindrance effect of DIPEA, the phenyl group could not directly replace the -I of intermediate **A** in the process of **A** + **C** → **D**, thus inhibiting the occurrence of the Ullmann reaction.

## 3. Materials and Methods

### 3.1. Materials

All solvents and reagents were purchased at the highest commercial quality grade and were used without further purification, unless otherwise stated. All reactions were carried out under an atmosphere of nitrogen, unless otherwise stated. Purification by column chromatography was performed using E. Merck silica (60, particle size 0.040–0.045 mm). The FT-IR spectra were obtained via Nicolet IS50 FT-IR spectrometers. The Pd loading in the nanocatalyst was analyzed by an Agilent 720ES type inductively coupled plasma optical emission spectroscopy (ICP-OES) instrument. NMR spectra were recorded at room temperature on Bruker AVANCE III spectrometers. GC-MS analysis was recorded on an Agilent 5977B MSD Series spectrometer. HRMS (high-resolution mass spectra) were recorded on a Shimadzu LCMS-IT-TOF mass spectrometer.

### 3.2. Preparation of the Pd@COF-TB Nanocatalyst

A Schlenk tube was used for the addition of COF-TB (200 mg), Pd(OAc)_2_ (20 mg) and Diox (60 mL), followed by agitation at 70 °C for 15 h. After that, a yellow–green solid was obtained by high-speed centrifugation. Subsequently, the solid was washed and filtered with acetonitrile, and dried overnight in a fume hood to obtain a yellow–green powder of the Pd@COF-TB nanocatalyst (208.6 mg) [34].

### 3.3. Synthesis of Sodium S-phenyl Sulfurothioate (***3a***)

A Schlenk tube was used for the addition of iodobenzene (2.04 g, 10 mmol), Na_2_S_2_O_3_ (3.15 g, 20 mmol), Pd@COF-TB (0.20 g), DIPEA (2.60 g, 20 mmol) and DMF (15 mL) in a N_2_ atmosphere. The mixture was then stirred at 120 °C for 4 h. When the reaction was finished, the mixture was cooled to room temperature, quenched with saturated aqueous NaCl (15 mL) and then vigorously stirred at room temperature for another 5 h. Then, the precipitated solid in this system was filtered and washed with saturated aqueous NaCl and n-hexane to give sodium S-phenyl sulfurothioate [44] (0.95 g, 45%) as a white solid. ^1^H NMR (400 MHz, Methanol-d4) δ 7.81–7.63 (m, 2H), 7.61–7.32 (m, 3H). HRMS (ESI-TOF) m/z calcd. for: C_6_H_5_O_3_S_2_ [M-Na]^−^: 188.9680, found: 188.9680.

### 3.4. Synthesis of 4-Methyldiphenyl Sulfide (***4a***)

A Schlenk tube was used for the addition of iodobenzene (0.10 g, 0.5 mmol), sodium S-phenyl Sulfurothioate (0.09 g, 0.5 mmol), Pd@COF-TB (20 mg), DIPEA (0.26 g, 2.0 mmol) and DMF (3 mL) in a N_2_ atmosphere. The mixture was then stirred at 120 °C for 10 h. When the reaction was finished, the mixture was cooled to room temperature, quenched by H_2_O (3 mL) and extracted with ethyl acetate (3 mL × 3). Then the combined extract was washed with saturated aqueous NaCl (3 mL × 3), dried over anhydrous sodium sulfate and concentrated under vacuum. Purification by column chromatography on silica gel afforded 4-methyldiphenyl sulfide [45] (0.09 g, 89%) as a colorless liquid. ^1^H NMR (400 MHz, CDCl_3_) δ 7.29 (d, J = 8.2 Hz, 2H), 7.24 (q, J = 7.8 Hz, 4H), 7.18–7.13 (m, 1H), 7.11 (d, J = 8.2 Hz, 2H), 2.32 (s, 3H). ^13^C NMR (101 MHz, CDCl_3_) δ 137.73, 137.31, 132.56, 131.47, 130.34, 130.09, 129.20, 126.39. 21.34. GC-MS (EI) m/z calcd. for: C_13_H_12_S [M]^+^: 200.07, found: 200.14.

### 3.5. General Procedure for the Synthesis of Symmetrical Diaryl Sulfide

A Schlenk tube was used for the addition of aryl iodide (2.0 mmol), Na_2_S_2_O_3_ (0.63 g, 4.0 mmol), Pd@COF-TB (40 mg), DIPEA (0.52 g, 4.0 mmol) and DMF (6.0 mL) in N_2_ atmosphere. The mixture was then stirred at 120 °C and monitored by TLC and HPLC. When the reaction was finished, the mixture was cooled to room temperature, quenched by H_2_O (6 mL) and extracted with ethyl acetate (5 mL × 3). Then, the combined extract was washed with saturated aqueous NaCl (5 mL × 3), dried over anhydrous sodium sulfate and concentrated under vacuum. Purification by column chromatography on silica gel afforded the desired products and their detailed characterization data are reported in the Supporting Information.

## 4. Conclusions

In summary, we have developed a general and efficient Pd@COF-TB/DIPEA-catalyzed one-pot synthesis of symmetrical diaryl sulfides through a C-S coupling reaction with aryl iodides as the starting materials and Na_2_S_2_O_3_ as the sulfur source. An array of symmetrical diaryl sulfides have been synthesized in moderate to excellent yields through this protocol. As a good ligand, DIPEA, apart from providing an alkaline environment, could not only enhance the catalytic activity of Pd@COF-TB nanocatalyst, but also effectively inhibit the formation of biphenyl byproducts. Additionally, it has been confirmed that the aryl Bunte salts generated in situ from Na_2_S_2_O_3_ and aryl iodides are the real sulfur sources involved in this C-S coupling reaction. Lastly, a proposed mechanism of this Pd@COF-TB/DIPEA-catalyzed C-S coupling reaction was revealed in detail through many controlling experiments. This work has expanded the application of Pd@COF-TB nanocatalyst in a C-S coupling reaction, and further studies on other applications of Pd@COF-TB nanocatalyst are ongoing in our laboratory.

## Figures and Tables

**Table 1:** Pd loading determined by ICP-OES.

Entry	Content of Pd Tested by ICP-OES	Load of Pd in the Nanocatalyst (wt%)
1	0.126 ppm	4.72
2	0.123 ppm	4.61
3	0.127 ppm	4.76

**Table 2:** Optimization of conditions for the C-S coupling reaction ^1^.

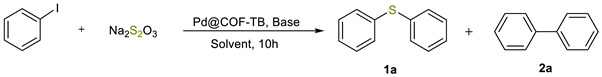						
Entry	Solvent	Base	Pd@COF-TB (mg)	Temp (°C)	Yield ^2^	
					1a	2a
1	DMF	KOH	20	100	36%	50%
2	DMSO	KOH	20	100	21%	67%
3	NMP	KOH	20	100	33%	52%
4	H_2_O	KOH	20	100	12%	35%
5	EtOH	KOH	20	100	28%	70%
6	PEG_200_	KOH	20	100	32%	59%
7	DMF	NaOH	20	100	30%	57%
8	DMF	K_2_CO_3_	20	100	16%	66%
9	DMF	Cs_2_CO_3_	20	100	24%	58%
10	DMF	NaOMe	20	100	33%	60%
11	DMF	Et_3_N	20	100	59%	17%
12	DMF	DBU	20	100	33%	0
13	DMF	DIPEA	20	100	80%	12%
14	DMF	DIPEA	0	100	0	0
15	DMF	DIPEA	10	100	61%	14%
16	DMF	DIPEA	15	100	70%	12%
17	DMF	DIPEA	25	100	80%	14%
18	DMF	DIPEA	30	100	80%	16%
19 ^3^	DMF	DIPEA	Pd(OAc)_2_, 2 mg	100	51%	35%
20	DMF	DIPEA	20	40	43%	18%
21	DMF	DIPEA	20	60	56%	15%
22	DMF	DIPEA	20	80	63%	16%
**23**	**DMF**	**DIPEA**	**20**	**120**	**94%**	**3%**
24	DMF	DIPEA	20	140	86%	4%

^1^ Typical conditions: iodobenzene (1.0 mmol), Na_2_S_2_O_3_ (2.0 mmol) and base (2.0 mmol) in 3 mL of solvent, 10 h, in N_2_. ^2^ The content of Pd in 2 mg Pd(OAc)_2_ is consistent with that in 20 mg Pd@COF-TB. ^3^ Determined by GC-MS.

**Table 3:** Substrate scope of symmetrical diaryl sulfides ^1^.

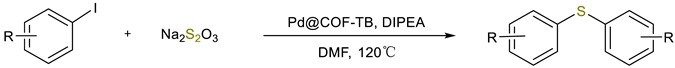
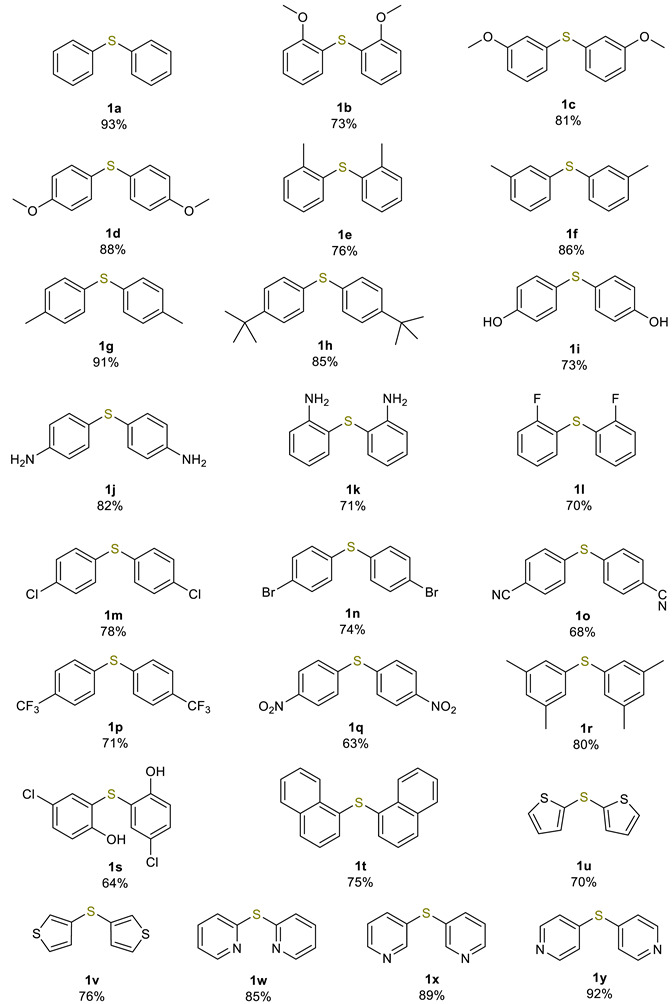

Reagents and conditions: aryl iodide (2.0 mmol), Na_2_S_2_O_3_ (0.63 g, 4.0 mmol), Pd@COF-TB (40 mg), and DIPEA (0.52 g, 4.0 mmol) in 6 mL DMF, 120 °C, in N_2_. ^1^ Isolated yield.

## Data Availability

Additional figures are available in the Supplementary Materials.

## Supplementary Materials

The following supporting information can be downloaded at: https://www.mdpi.com/article/10.3390/ijms232315360/s1. References are cited in [25,45,46,47,48,49,50,51,52].
