# Supplementary material for: Efficient Construction of Symmetrical Diaryl Sulfides via a Supported Pd Nanocatalyst-Catalyzed C-S Coupling Reaction
Source: Int J Mol Sci. 2022 Dec 6;23(23):15360. doi: 10.3390/ijms232315360 (PMC9738011; doi:10.3390/ijms232315360)

Supporting Information

Table of Contents

General Information S2

Preparation of the Pd@COF-TB Nanocatalyst S2

Synthesis of Sodium S-phenyl Sulfurothioate (3a) S3

Synthesis of 4-Methyldiphenyl Sulfide (4a) S4

General Procedure for the Synthesis of Symmetrical Diaryl Sulfide S6

Characterization of All Compounds S6

References S13

NMR Spectra S14

General Information

All solvents and reagents were purchased at the highest commercial quality grade and used without further purification, unless otherwise stated. All reactions were carried out under an atmosphere of nitrogen, unless otherwise stated. Reactions were monitored by HPLC and TLC. Column chromatography was performed using E. Merck silica (60, particle size 0.040 – 0.045 mm). TLC analysis was performed using 0.25 mm E. Merck silica plates (60F-254), using 254 nm UV light as the visualizing agent. HPLC analysis was performed on a 1220 Infinity II, Agilent with silica column (Supersil ODS2 5µm, 4.6 mm × 250 mm). Melting points were determined using a digital melting point apparatus (Shanghai INESA Physico-Optical Instrument Co., Ltd. SGW ® X-4B) and were uncorrected. The FT-IR spectra analysis was obtained on Nicolet IS50 FT-IR Spectrometers. The Pd loading in the nanocatalyst was analyzed by an Agilent 720ES type inductively coupled plasma optical emission spectroscopy (ICP-OES) instrument. ^{1}H NMR spectra was recorded at ambient temperature on 400 MHz NMR spectrometers (Bruker AVANCE III) using deuterated chloroform ($CDCl_3$) or deuterated dimethyl sulfoxide (DMSO-d_6) as solvent and tetramethylsilane (TMS, δ = 0) as internal reference. Chemical shifts are reported in parts per million (ppm) downfield and quoted to the nearest 0.01 ppm relative to the residual protons in in the NMR solvent ($CHCl_3$ = δ 7.26, DMSO-d_6 = δ 2.62), and coupling constants (J) are quoted in Hertz. Coupling constants were quoted to the nearest 0.1 Hz and multiplicity reported according to the following convention: s = singlet, d = doublet, t = triplet, q = quartet, m = multiplet, br = broad singlet, and associated combinations for example: dd=doublet of doublet, dt=doublet of triplet, tt=triplet of triplet. ^{13}C NMR spectra were measured at ambient temperature on 101 MHz NMR spectrometers (Bruker AVANCE III). Chemical shifts are reported in ppm from tetramethylsilane with the solvent resonance as the internal standard ($CDCl_3$ = δ 77.16, DMSO-d_6 = δ 39.52). GC-MS analysis was recorded on an Agilent 5977B MSD Series spectrometer. HRMS (high-resolution mass spectra) were recorded on a Shimadzu LCMS-IT-TOF mass spectrometer by electrospray ionization time of flight reflectron experiments.

Preparation of the Pd@COF-TB Nanocatalyst

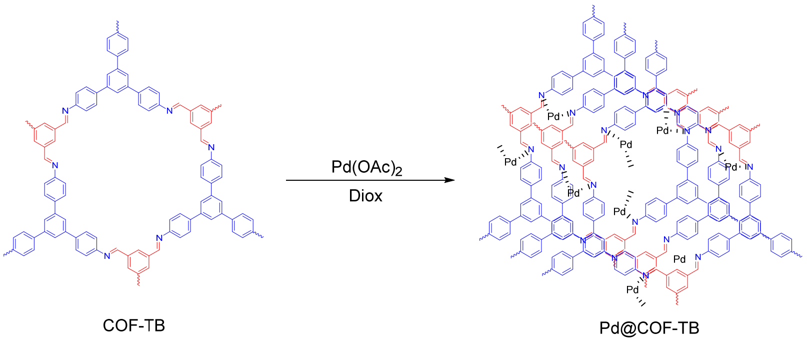

To a Schlenk tube were added COF-TB (200 mg), $Pd(OAc)_2$ (20 mg) and Diox (60 mL), followed by agitation at 70 °C for 15 h. After that, a yellow-green solid was obtained by high-speed centrifugation. Subsequently, the solid was washed and filtered with acetonitrile, and dried overnight in a fume hood to get yellow-green powder Pd@COF-TB nanocatalyst (208.6mg).

Synthesis of Sodium S-phenyl Sulfurothioate (3a)

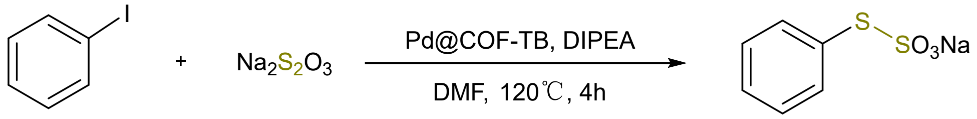

To a Schlenk tube were added iodobenzene (2.04 g, 10 mmol), $Na_2S_2O_3$ (3.15 g, 20 mmol), Pd@COF-TB (0.20 g), DIPEA (2.60 g, 20 mmol) and DMF (15 mL) in N_2 atmosphere. The mixture was then stirred at 120°C for 4 h. When the reaction was finished, the mixture was cooled to room temperature, quenched with saturated aqueous NaCl (15 mL) and then vigorously stirred at room temperature for another 5 h. Then, the precipitated solid in this system was filtered and washed with saturated aqueous NaCl and n-hexane to give sodium S-phenyl sulfurothioate [1] (0.95 g, 45%) as a white solid. **^{1}H NMR** (400 MHz, Methanol-d4) δ 7.81 – 7.63 (m, 2H), 7.61 – 7.32 (m, 3H). **HRMS** (ESI-TOF) m/z calcd. for: $C_6H_5O_3S_2$ $[M-Na]^-$: 188.9680, found: 188.9680.

^{1}H NMR

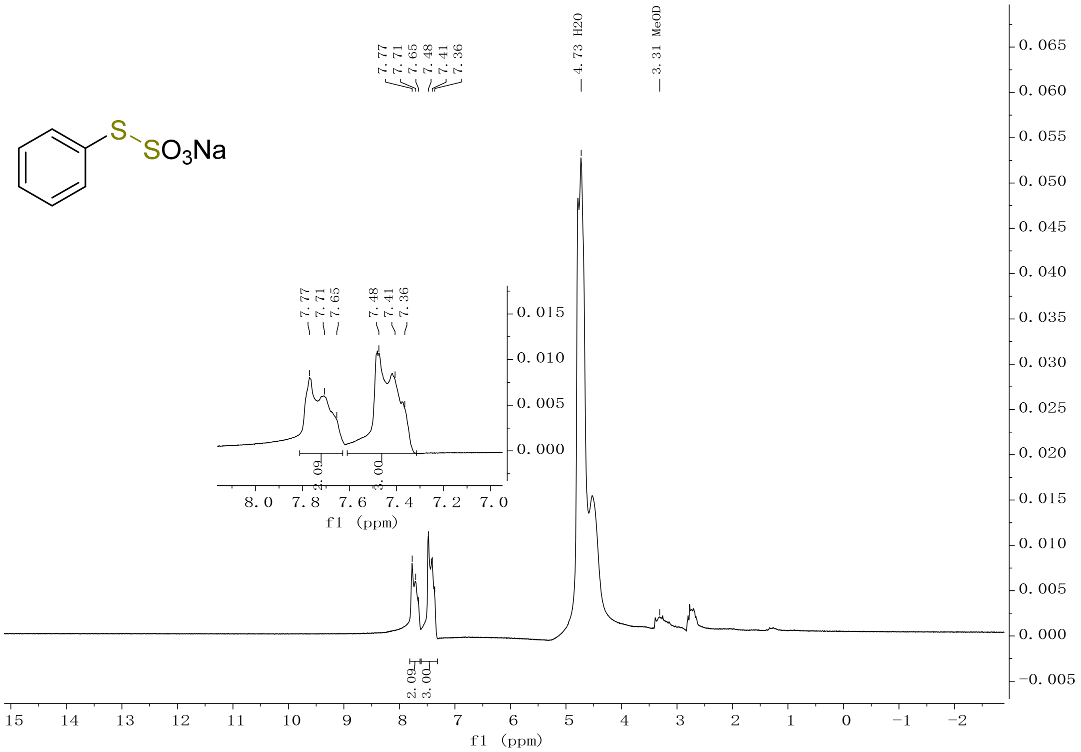

HRMS

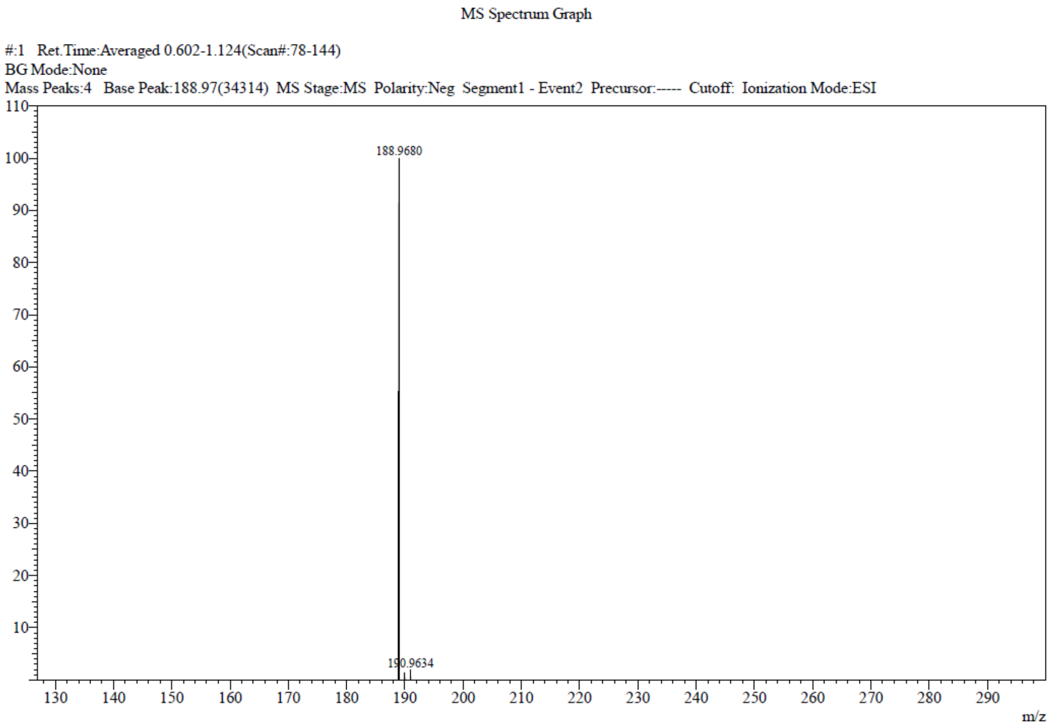

Synthesis of 4-Methyldiphenyl Sulfide (4a)

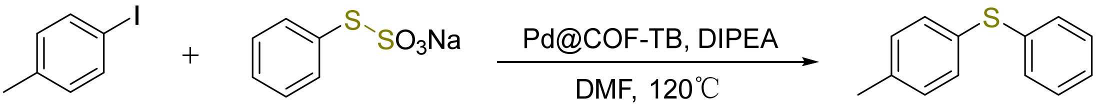

To a Schlenk tube were added iodobenzene (0.10 g, 0.5 mmol), Sodium S-phenyl Sulfurothioate (0.09 g, 0.5 mmol), Pd@COF-TB (20 mg), DIPEA (0.26 g, 2.0 mmol) and DMF (3 mL) in N_2 atmosphere. The mixture was then stirred at 120 °C for 10 h. When the reaction was finished, the mixture was cooled to room temperature, quenched by H_2O (3 mL) and extracted with ethyl acetate (3 mL×3). Then the combined extract was washed with saturated aqueous NaCl (3 mL×3), dried over anhydrous sodium sulfate and concentrated under vacuum. Purification by column chromatography on silica gel affords 4-methyldiphenyl sulfide [2] (0.09 g, 89%) as colorless liquid. ^{1}H NMR (400 MHz, $CDCl_3$) δ 7.29 (d, J = 8.2 Hz, 2H), 7.24 (q, J = 7.8 Hz, 4H), 7.18 – 7.13 (m, 1H), 7.11 (d, J = 8.2 Hz, 2H), 2.32 (s, 3H). ^{13}C NMR (101 MHz, $CDCl_3$) δ 137.73, 137.31, 132.56, 131.47, 130.34, 130.09, 129.20, 126.39. 21.34. GC-MS (EI) m/z calcd. for: $C_{13}H_{12}S$ $[M]^+$: 200.07, found: 200.14.

^{1}H NMR

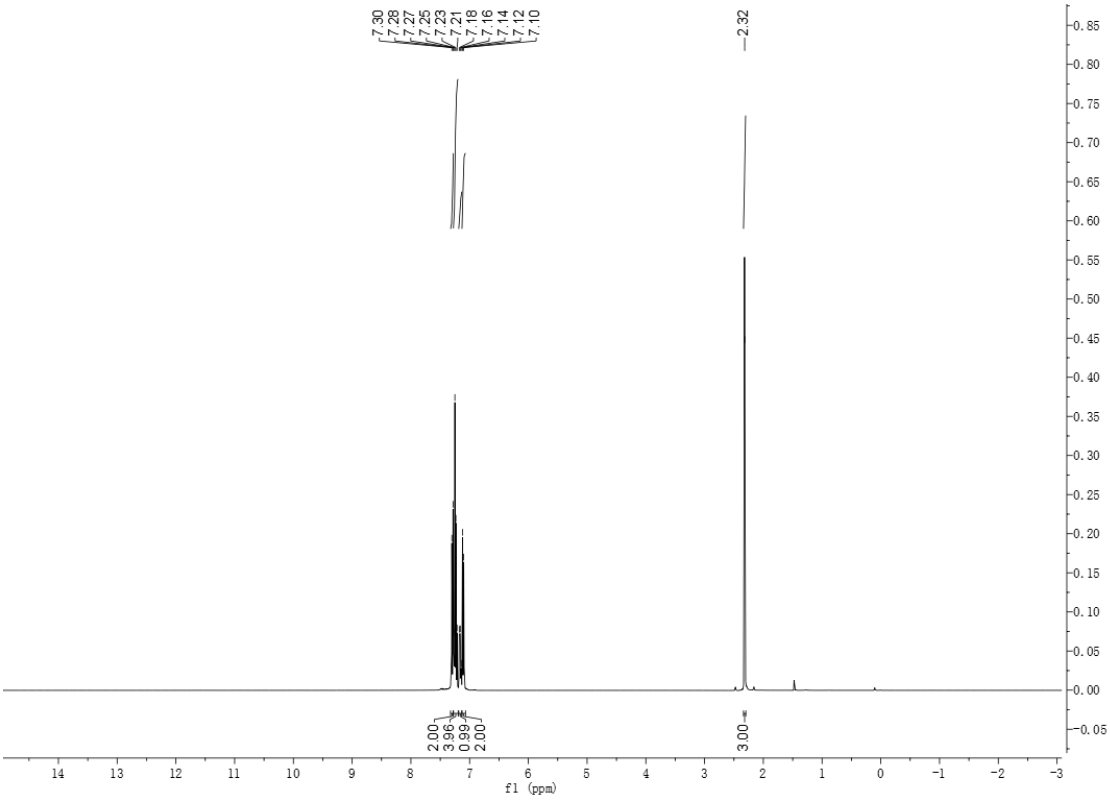

^{13}C NMR

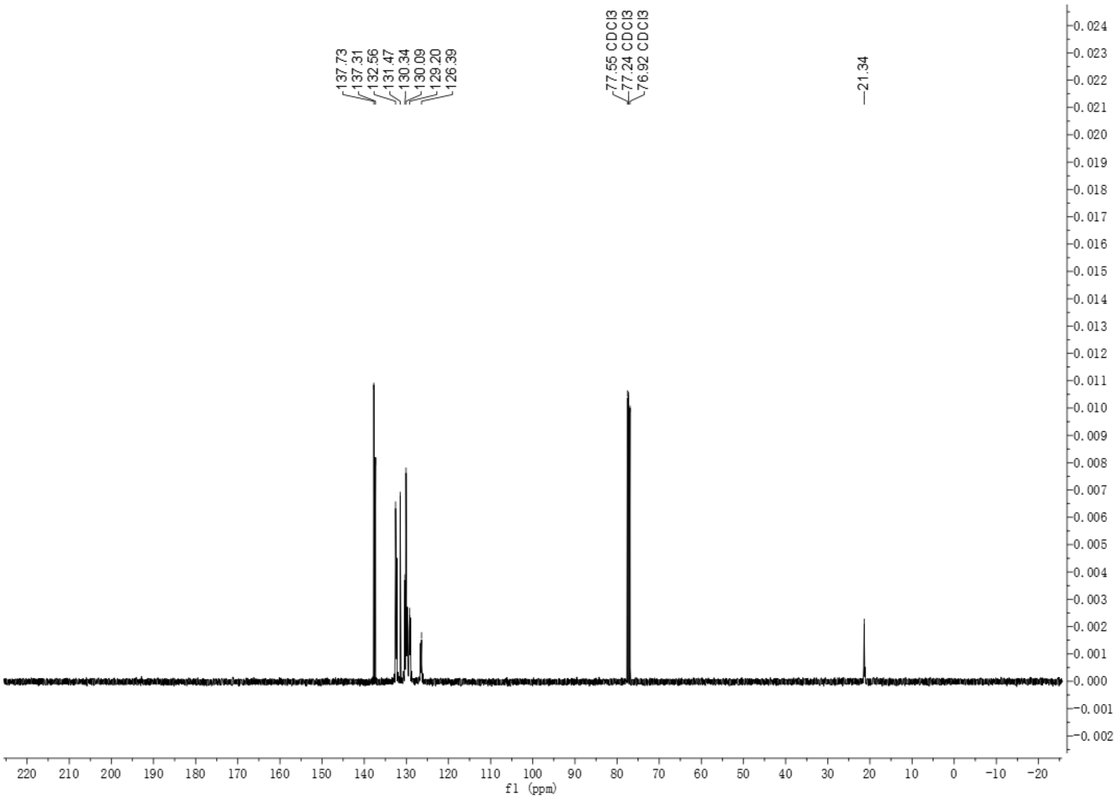

General Procedure for the Synthesis of Symmetrical Diaryl Sulfide

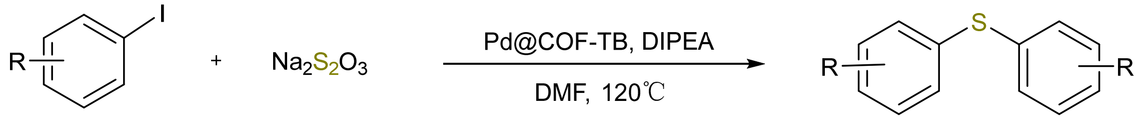

To a Schlenk tube were added aryl iodide (2.0 mmol), $Na_2S_2O_3$ (0.63 g, 4.0 mmol), Pd@COF-TB (40 mg), DIPEA (0.52 g, 4.0 mmol) and DMF (6.0 mL) in N_2 atmosphere. The mixture was then stirred at 120°C, and monitored by TLC and HPLC. When the reaction was finished, the mixture was cooled to room temperature, quenched by H_2O (6 mL) and extracted with ethyl acetate (5 mL×3). Then the combined extract was washed with saturated aqueous NaCl (5 mL×3), dried over anhydrous sodium sulfate and concentrated under vacuum. Purification by column chromatography on silica gel affords the desired products.

Characterization of All Compounds

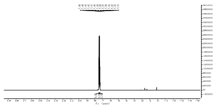

Diphenyl sulfide（1a）

Flash column chromatography on a silica gel (petroleum ether) afforded the product [3] (0.17 g, 93%) as pale yellow oil. **^{1}H NMR** (400 MHz, $CDCl_3$) δ 7.47 – 7.42 (m, 4H), 7.42 – 7.36 (m, 4H), 7.36 – 7.30 (m, 2H); **^{13}C NMR** (101 MHz, $CDCl_3$) δ 135.82, 131.08, 129.24, 127.09. **GC-MS** (EI) m/z calcd. for: $C_{12}H_{10}S$ [M]: 186.05, found: 186.03.

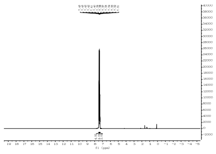

Bis(2-methoxyphenyl) sulfide（1b）

Flash column chromatography on a silica gel (petroleum ether) afforded the product [4] (0.18 g, 73%) as a light yellow solid, **mp.** 72.8 – 74.0 °C. **^{1}H NMR** (400 MHz, DMSO-d_6) δ 7.29 (d, J = 7.0Hz, 2H), 7.08 (d, J = 5.8 Hz, 2H), 6.90 (d, J = 9.8 Hz, 4H), 3.80 (s, 6H); **^{13}C NMR** (101 MHz, DMSO-d_6) δ 156.94, 130.74, 128.27, 121.17, 120.81, 111.07, 55.35. **GC-MS** (EI) m/z calcd. for: $C_{14}H_{14}O_2S$ [M]: 246.07, found: 246.06.

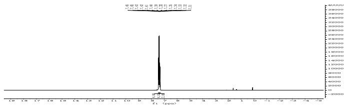

Bis(3-methoxyphenyl) sulfide（1c）

Flash column chromatography on a silica gel (petroleum ether) afforded the product [4] (0.20 g, 81%) as a yellow solid, **mp.** 47.9 – 48.7 °C. **^{1}H NMR** (400 MHz, DMSO-*d*$_6$) δ 7.26 (t, J = 8.2 Hz, 2H), 6.91 – 6.84 (m, 6H), 3.70 (s, 6H); **^{13}C NMR** (101 MHz, DMSO-*d*$_6$) δ 159.40, 135.42, 129.94, 122.41, 115.61, 112.61, 54.71. **GC-MS** (EI) m/z calcd. for: $C_{14}H_{14}O_2S$ [M]: 246.07, found: 246.09.

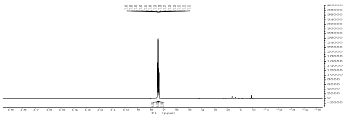

Bis(4-methoxyphenyl) sulfide（1d）

Flash column chromatography on a silica gel (petroleum ether) afforded the product [4] (0.22 g, 88%) as a yellow solid, **mp.** 49.0 – 50.2 °C. **^{1}H NMR** (400 MHz, $CDCl_3$) δ 7.31 – 7.26 (m, 4H), 6.84 (d, J = 9.0 Hz, 4H), 3.78 (s, 6H); **^{13}C NMR** (101 MHz, $CDCl_3$) δ 159.09, 132.84, 127.53, 114.87, 55.44. **GC-MS** (EI) m/z calcd. for: $C_{14}H_{14}O_2S$ [M]: 246.07, found: 246.08.

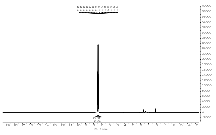

2,2´-Dimethyldiphenyl sulfide（1e）

Flash column chromatography on a silica gel (petroleum ether) afforded the product [5] (0.17 g, 78%) as a white solid, **mp.** 65.3 – 66.1 °C. **^{1}H NMR** (400 MHz, DMSO-*d*$_6$) δ 7.32 (d, *J* = 7.6 Hz, 2H), 7.23 (t, *J* = 7.4 Hz, 2H), 7.16 (t, *J* = 7.6 Hz, 2H), 7.00 (d, *J* = 7.8 Hz, 2H), 2.31 (s, 6H); **^{13}C NMR** (101 MHz, DMSO-*d*$_6$) δ 138.19, 133.29, 130.67, 130.64, 127.46, 127.01, 19.90. **GC-MS** (EI) m/z calcd. for: $C_{14}H_{14}S$ [M]: 214.08, found: 214.09.

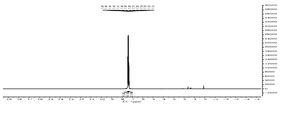

3,3´-Dimethyldiphenyl sulfide（1f）

Flash column chromatography on a silica gel (petroleum ether) afforded the product [5] (0.18 g, 86%) as colorless liquid. **^{1}H NMR** (400 MHz, DMSO-*d*$_6$) δ 7.25 (t, *J* = 7.8 Hz, 2H), 7.16 (s, 2H), 7.12 – 7.07 (m, 4H), 2.26 (s, 6H); **^{13}C NMR** (101 MHz, DMSO-*d*$_6$) δ 138.90, 134.62, 131.10, 129.31, 128.09, 127.78, 20.80. **GC-MS** (EI) m/z calcd. for: $C_{14}H_{14}S$ [M]: 214.08, found: 214.02.

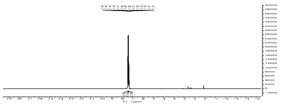

4,4′-Dimethyldiphenyl sulfide（1g）

Flash column chromatography on a silica gel (petroleum ether) afforded the product [5] (0.19 g, 91%) as a white solid, **mp.** 55.2 – 56.3 °C. **^{1}H NMR** (400 MHz, $CDCl_3$) δ 7.23 (d, *J* = 8.2 Hz, 4H), 7.10 (d, *J* = 8.2 Hz, 4H), 2.33 (s, 6H); **^{13}C NMR** (101 MHz, $CDCl_3$) δ 137.00, 132.73, 131.14, 130.01, 21.19. **GC-MS** (EI) m/z calcd. for: $C_{14}H_{14}S$ [M]: 214.08, found: 214.05.

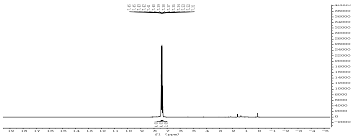

4,4′-Di-tert-butyldiphenyl sulfide（1h）

Flash column chromatography on a silica gel (petroleum ether) afforded the product [6] (0.25 g, 85%) as a white solid, **mp.** 79.6 – 80.8 °C. **^{1}H NMR** (400 MHz, DMSO-d_6) δ 7.36 (d, *J* = 8.0 Hz, 4H), 7.22 (d, *J* = 7.8 Hz, 4H), 1.24 (s, 18H); **^{13}C NMR** (101 MHz, DMSO-d_6) δ 149.65, 131.38, 130.23, 126.03, 33.94, 30.65. **GC-MS** (EI) m/z calcd. for: $C_{20}H_{26}S$ [M]: 298.18, found: 298.14.

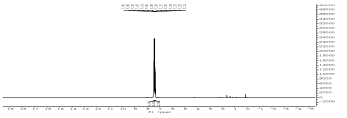

Bis(p-hydroxhphenyl) sulfide（1i）

Flash column chromatography on a silica gel (ethyl acetate: petroleum ether = 1:50) afforded the product [3] (0.16 g, 73%) as a white solid, **mp.** 158.9 – 159.8 °C. **^{1}H NMR** (400 MHz, DMSO-d_6) δ 9.64 (d, *J* = 11.6 Hz, 2H), 7.31 – 7.01 (m, 4H), 6.74 (dd, *J* = 12.4, 7.8 Hz, 4H); **^{13}C NMR** (101 MHz, DMSO-d_6) δ 156.94, 132.71, 124.61, 116.26. **GC-MS** (EI) m/z calcd. for: $C_{12}H_{10}O_2S$ [M]: 218.04, found: 218.01.

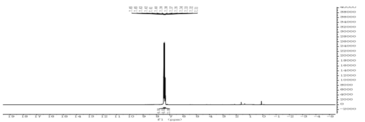

Bis(4-aminophenyl)sulphide（1j）

Flash column chromatography on a silica gel (ethyl acetate: petroleum ether = 1:5) afforded the product [3] (0.18 g, 82%) as a brown solid, **mp.** 110.4 – 111.2 °C. **^{1}H NMR** (400 MHz, $CDCl_3$) δ 7.15 (d, *J* = 8.6 Hz, 4H), 6.60 (d, *J* = 8.6 Hz, 4H), 3.67 (s, 4H); **^{13}C NMR** (101 MHz, $CDCl_3$)

δ 145.64, 132.77, 125.00, 115.77. **GC-MS** (EI) m/z calcd. for: $C_{12}H_{12}N_2S$ [M]: 216.07, found: 216.05.

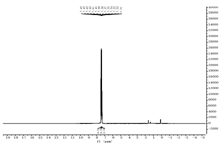

Bis(2-aminophenyl)sulphide（1k）

Flash column chromatography on a silica gel (ethyl acetate: petroleum ether = 1:5) afforded the product [7] (0.15 g, 71%) as an off-white solid, **mp.** 86.8 – 88.2 °C. **^{1}H NMR** (400 MHz, $CDCl_3$) δ 7.22 (d, *J* = 7.8 Hz, 2H), 7.14 – 7.10 (m, 2H), 6.78 – 6.66 (m, 4H), 3.99 (s, 4H); **^{13}C NMR** (101 MHz, $CDCl_3$) δ 146.71, 133.41, 129.22, 119.05, 117.21, 115.53. **GC-MS** (EI) m/z calcd. for: $C_{12}H_{12}N_2S$ [M]: 216.07, found: 216.04.

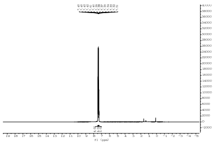

Bis(2-fluorophenyl) sulfide（1l）

Flash column chromatography on a silica gel (petroleum ether) afforded the product [4] (0.16 g, 70%) as light yellow oil. **^{1}H NMR** (400 MHz, $CDCl_3$) δ 7.31 – 7.23 (m, 4H), 7.13 – 7.05 (m, 4H); **^{13}C NMR** (101 MHz, $CDCl_3$) δ 161.36 (d, J = 247.2 Hz), 133.34 (d, J = 1.0 Hz), 129.75 (d, J = 7.8 Hz), 124.94 (d, J = 4.0 Hz), 116.10 (d, J = 22.1 Hz); **^{19}F NMR** (376 MHz, $CDCl_3$) δ -108.61. **GC-MS** (EI) m/z calcd. for: $C_{12}H_8F_2S$ [M]: 222.03, found:222.03.

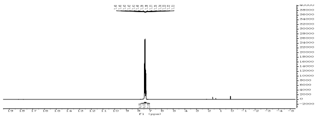

Bis(4-chlorophenyl) sulfide（1m）

Flash column chromatography on a silica gel petroleum ether) afforded the product [5] (0.20 g, 78%) as a light yellow solid, **mp.** 89.9 – 91.3 °C. **^{1}H NMR** (400 MHz, $CDCl_3$) δ 7.28 (d, *J* = 8.8 Hz, 4H), 7.24 (d, *J* = 8.8 Hz, 4H); **^{13}C NMR** (101 MHz, $CDCl_3$) δ 133.93, 133.46, 132.31, 129.50. **GC-MS** (EI) m/z calcd. for: $C_{12}H_8Cl_2S$ [M]: 253.97, found: 253.95.

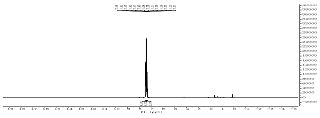

Bis(4-bromophenyl) sulphide（1n）

Flash column chromatography on a silica gel (petroleum ether) afforded the product [5] (0.25 g, 74%) as a white solid, **mp.** 113.8 – 114.6 °C. **^{1}H NMR** (400 MHz, $CDCl_3$) δ 7.43 (d, *J* = 8.6 Hz, 4H), 7.18 (d, *J* = 8.6 Hz, 4H); **^{13}C NMR** (101 MHz, $CDCl_3$) δ 134.47, 132.58, 132.45, 121.50. **GC-MS** (EI) m/z calcd. for: $C_{12}H_8Br_2S$ [M]: 343.87, found: 343.83.

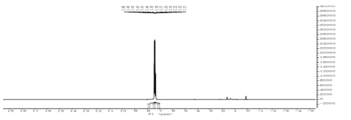

4,4′-Dicyanodiphenyl sulfide（1o）

Flash column chromatography on a silica gel (ethyl acetate: petroleum ether = 1:10) afforded the product [3] (0.16 g, 68%) as milky white solid, **mp.** 134.2 – 135.6 °C. **^{1}H NMR** (400 MHz, $CDCl_3$) δ 7.61 – 7.57 (m, 4H), 7.54 – 7.39 (m, 4H); **^{13}C NMR** (101 MHz, $CDCl_3$) δ 140.71, 133.07, 131.25, 126.60, 111.47. **GC-MS** (EI) m/z calcd. for: $C_{14}H_8N_2S$ [M]:236.04, found: 236.05.

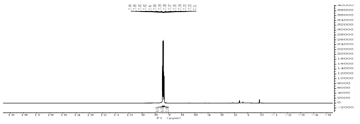

Bis(4-(trifluoromethyl)phenyl)sulfane（1p）

Flash column chromatography on a silica gel (ethyl acetate: petroleum ether = 1:20) afforded the product [5] (0.24 g, 71%) as a white solid, **mp.** 75.6 – 77.3 °C. **^{1}H NMR** (400 MHz, $CDCl_3$) δ 7.58 (d, *J* = 8.2 Hz, 4H), 7.44 (d, *J* = 8.6 Hz, 4H); **^{13}C NMR** (101 MHz, $CDCl_3$) δ 139.58, 131.09, 129.74 (q, *J* = 33.0 Hz), 126.35 (q, *J* = 3.8 Hz), 122.59 (q, *J* = 1.0 Hz); **^{19}F NMR** (376 MHz, $CDCl_3$) δ -62.63. **GC-MS** (EI) m/z calcd. for: $C_{14}H_8F_6S$ [M]: 322.03, found: 322.03.

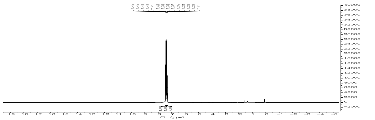

4,4′-Dinitrodiphenyl sulfide（1q）

Flash column chromatography on a silica gel (ethyl acetate: petroleum ether = 1:10) afforded the product [3] (0.17 g, 63%) as an orange solid, **mp.** 152.1 – 153.0 °C. **^{1}H NMR** (400 MHz, DMSO-*d*$_6$) δ 8.28 – 8.21 (m, 4H), 7.68 – 7.61 (m, 4H); **^{13}C NMR** (101 MHz, DMSO-*d*$_6$) δ 146.71, 142.11, 131.31, 124.70. **GC-MS** (EI) m/z calcd. for: $C_{12}H_8N_2O_4S$ [M]:276.02, found: 276.01.

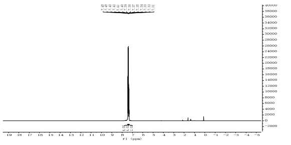

Bis(3,5-dimethylphenyl) sulfide（1r）

Flash column chromatography on a silica gel (petroleum ether) afforded the product [8] (0.19 g, 80%) as a white solid, **mp.** 155.3 – 156.6 °C. **^{1}H NMR** (400 MHz, DMSO-d_6) δ 6.92 (s, 4H), 6.85 (s, 2H), 2.17 (s, 12H); **^{13}C NMR** (101 MHz, DMSO-d_6) δ 138.45, 134.56, 128.82, 128.28, 20.63. **GC-MS** (EI) m/z calcd. for: $C_{16}H_{18}S$ [M]: 242.11, found: 242.11.

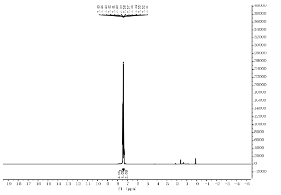

Bis(2-hydroxy-5-chlorophenyl) sulfide（1s）

Flash column chromatography on a silica gel (ethyl acetate: petroleum ether = 1:10) afforded the product [9] (0.18 g, 64%) as a white solid, **mp.** 180.5 – 181.0 °C. **^{1}H NMR** (400 MHz, DMSO-d_6) δ 10.36 (s, 2H), 7.19 (dd, *J* = 8.6, 2.6 Hz, 2H), 6.96 – 6.90 (m, 4H); **^{13}C NMR** (101 MHz, DMSO-d_6) δ 154.85, 130.15, 128.30, 122.88, 121.32, 116.75. **GC-MS** (EI) m/z calcd. for: $C_{12}H_8Cl_2O_2S$ [M]: 285.96, found: 285.94.

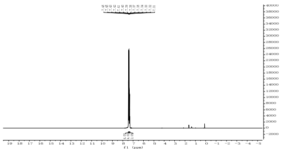

Di(1-naphthyl) sulfide（1t）

Flash column chromatography on a silica gel (petroleum ether) afforded the product [3] (0.21 g, 75%) as a white solid, **mp.** 111.0– 111.9 °C. **^{1}H NMR** (400 MHz, $CDCl_3$) δ 8.52 – 8.52 (m, 2H), 7.97 – 7.89 (m, 2H), 7.81 (d, *J* = 7.8 Hz, 2H), 7.61 – 7.54 (m, 4H), 7.42 – 7.30 (m, 4H); **^{13}C NMR** (101 MHz, $CDCl_3$) δ 134.27, 132.77, 132.58, 130.07, 128.75, 128.14, 126.91, 126.57, 126.03, 125.24. **GC-MS** (EI) m/z calcd. for: $C_{20}H_{14}S$ [M]: 286.08, found: 286.05.

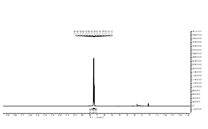

Dithienyl sulphide（1u）

Flash column chromatography on a silica gel (petroleum ether) afforded the product [3] (0.14 g, 70%) as brownish yellow oil. **^{1}H NMR** (400 MHz, $CDCl_3$) δ 7.36 – 7.27 (m, 2H), 7.20 (dd, J =

6.3, 3.5 Hz, 2H), 6.98 – 6.88 (m, 2H); ^{13}**C NMR** (101 MHz, $CDCl_3$) δ 135.63, 133.06, 129.97. **GC-MS** (EI) m/z calcd. for: $C_8H_6S_3$ [M]: 197.96, found: 197.96.

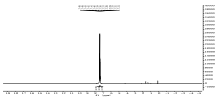

3-Thiophen-3-ylsulfanyl-thiophene（1v）

Flash column chromatography on a silica gel (petroleum ether) afforded the product [10] (0.15 g, 76%) as yellow oil. ^{1}**H NMR** (400 MHz, DMSO-d_6) δ 7.63 (dd, J = 5.0, 3.0 Hz, 2H), 7.49 (dd, J = 3.0, 1.2 Hz, 2H), 7.03 (dd, J = 5.0, 1.4 Hz, 2H); ^{13}**C NMR** (101 MHz, DMSO-d_6) δ 129.83, 129.24, 127.40, 125.34. **GC-MS** (EI) m/z calcd. for: $C_8H_6S_3$ [M]: 197.96, found: 197.95.

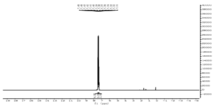

Di(2-pyridyl) sulfide（1w）

Flash column chromatography on a silica gel (ethyl acetate: petroleum ether = 1:10) afforded the product [5] (0.16 g, 85%) as a light yellow solid, mp. 220.3 – 221.2 °C. ^{1}**H NMR** (400 MHz, $CDCl_3$) δ 8.44 (d, J = 4.0 Hz, 2H), 7.56 – 7.50 (m, 2H), 7.38 – 7.32 (m, 2H), 7.11 – 7.04 (m, 2H); ^{13}**C NMR** (101 MHz, $CDCl_3$) δ 156.85, 150.20, 137.17, 125.93, 121.86. **GC-MS** (EI) m/z calcd. for: $C_{10}H_8N_2S$ [M]: 188.04, found: 188.03.

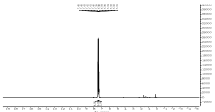

Di(3-pyridyl) sulfide（1x）

Flash column chromatography on a silica gel (ethyl acetate: petroleum ether = 1:10) afforded the product [7] (0.16 g, 89%) as a yellow solid, mp. 40.1 – 41.2 °C. ^{1}**H NMR** (400 MHz, DMSO-d_6) δ 8.55 (d, J = 2.4 Hz, 2H), 8.50 (dd, J = 4.8, 1.6 Hz, 2H), 7.76 (dt, J = 8.0, 2.0 Hz, 2H), 7.38 (dd, J = 8.0, 4.6 Hz, 2H); ^{13}**C NMR** (101 MHz, DMSO-d6) δ 150.67, 148.36, 138.43, 130.84, 124.27. **GC-MS** (EI) m/z calcd. for: $C_{10}H_8N_2S$ [M]: 188.04, found: 188.05.

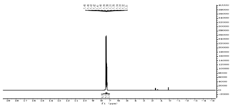

Di(4-pyridyl) sulfide（1y）

Flash column chromatography on a silica gel (ethyl acetate: petroleum ether = 1:10) afforded

the product [5] (0.17 g, 92%) as a white solid, **mp.** 71.3 – 72.4 °C. **^{1}H NMR** (400 MHz, $CDCl_3$) δ 8.51 (d, *J* = 6.6 Hz, 4H), 7.21 (d, *J* = 6.4 Hz, 4H); **^{13}C NMR** (101 MHz, $CDCl_3$) δ 150.37, 143.95, 124.79. **GC-MS** (EI) m/z calcd. for: $C_{10}H_8N_2S$ [M]: 188.04, found: 188.02.

References

1. Tan, W.; Jänsch, N.; Öhlmann, T.; Meyer-Almes, F.-J.; Jiang, X. Thiocarbonyl surrogate via combination of potassium sulfide and chloroform for dithiocarbamate construction. *Org. Lett.* **2019**, *21*, 7484-7488.
2. Xu, J.; Lu, F.; Sun, L.; Huang, M.; Jiang, J.; Wang, K.; Ouyang, D.; Lu, L.; Lei, A. Electrochemical reductive cross-coupling of acyl chlorides and sulfinic acids towards the synthesis of thioesters. *Green Chem.* **2022**, *24*, 7350-7354.
3. Ghodsinia, S.S.E.; Akhlaghinia, B. Cu I anchored onto mesoporous SBA-16 functionalized by aminated 3-glycidyloxypropyltrimethoxysilane with thiosemicarbazide (SBA-16/GPTMS-TSC-Cu I): a heterogeneous mesostructured catalyst for S-arylation reaction under solvent-free conditions. *Green chem.* **2019**, *21*, 3029-3049.
4. Li, X.; Du, J.; Zhang, Y.; Chang, H.; Gao, W.; Wei, W. Synthesis and nano-Pd catalyzed chemoselective oxidation of symmetrical and unsymmetrical sulfides. *Org. Biomol. Chem.* **2019**, *17*, 3048-3055.
5. Zhao, P.; Yin, H.; Gao, H.; Xi, C. Cu-catalyzed synthesis of diaryl thioethers and S-cycles by reaction of aryl iodides with carbon disulfide in the presence of DBU. *J. Org. Chem.* **2013**, *78*, 5001-5006.
6. Kollár, L.; Rao, Y.V.R.; Zugó, A.; Pongrácz, P. Palladium-catalysed thioetherification of aryl and alkenyl iodides using 1, 3, 5-trithiane as sulfur source. *Tetrahedron* **2022**, *104*, 132602.
7. Liu, Y.; Kim, J.; Seo, H.; Park, S.; Chae, J. Copper (II)-Catalyzed Single-Step Synthesis of Aryl Thiols from Aryl Halides and 1, 2-Ethanedithiol. *Adv. Synth. Catal.* **2015**, *357*, 2205-2212.
8. Zhang, Y.; Iiu, II; Chen, J. Efficient synthesis of diaryl sulfides by copper-catalysed coupling of aryl halides and thioacetate in water. *J. Chem. Res.* **2013**, *37*, 19-21.
9. Csokai, V.; Gruen, A.; Balázs, B.; Tóth, G.; Horváth, G.; Bitter, I. Unprecedented cyclizations of calix [4] arenes with glycols under the mitsunobu protocol, part 2.1 O, O-and O, S-bridged calixarenes. *Org. Lett.* **2004**, *6*, 477-480.
10. May, L.; Müller, T.J.J. Electron-Rich Phenothiazine Congeners and Beyond: Synthesis and Electronic Properties of Isomeric Dithieno [1, 4] thiazines. *Chem. Eur. J.* **2020**, *26*, 12111-12118.

NMR Spectra

Diphenyl sulfide（1a）

^{1}H NMR

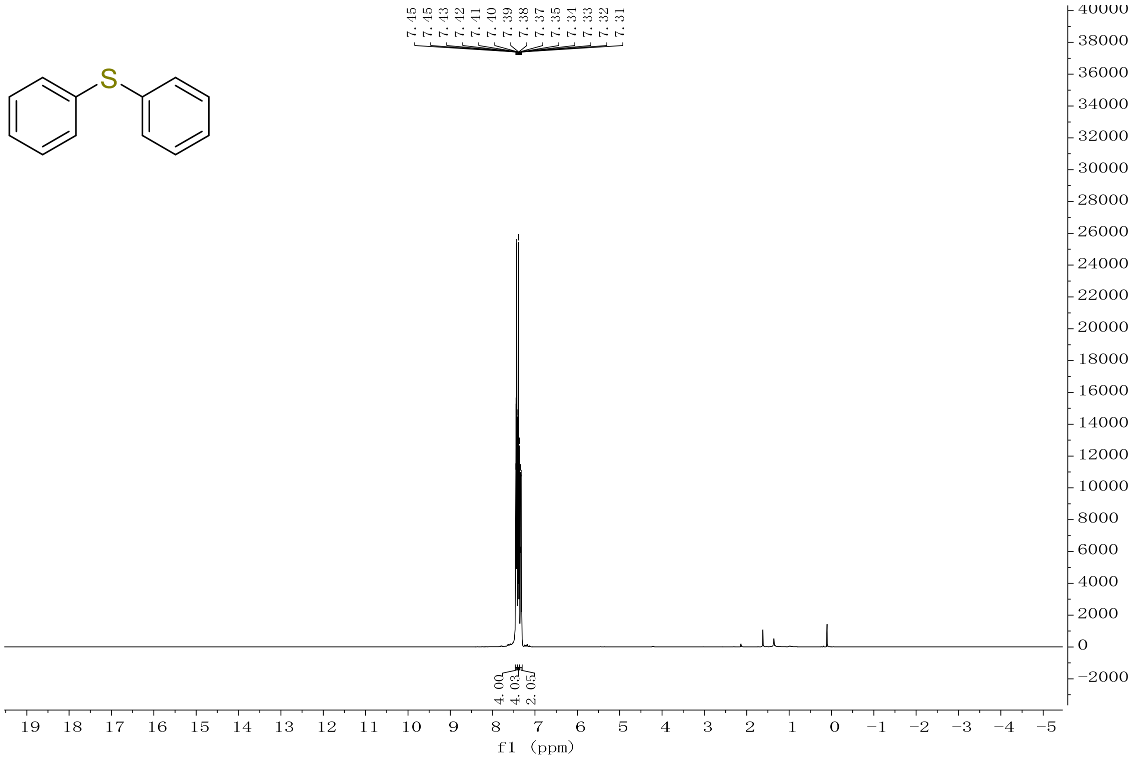

^{13}C NMR

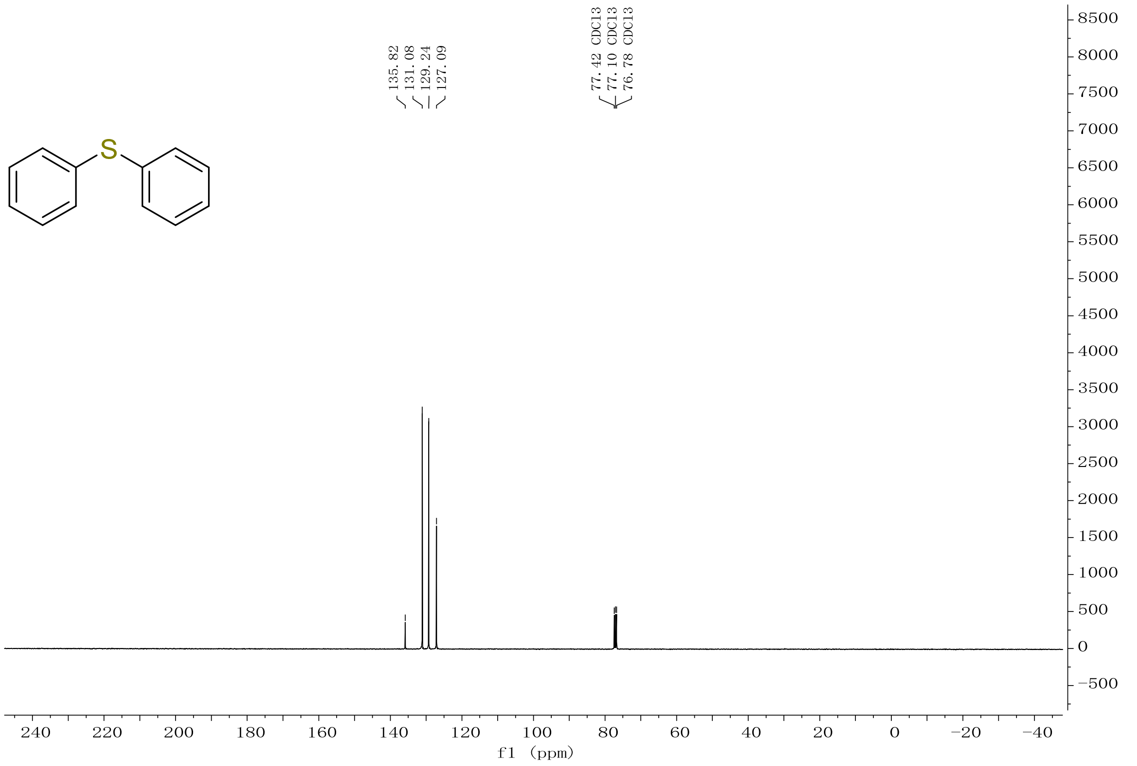

Bis(2-methoxyphenyl) sulfide（1b）

^{1}H NMR

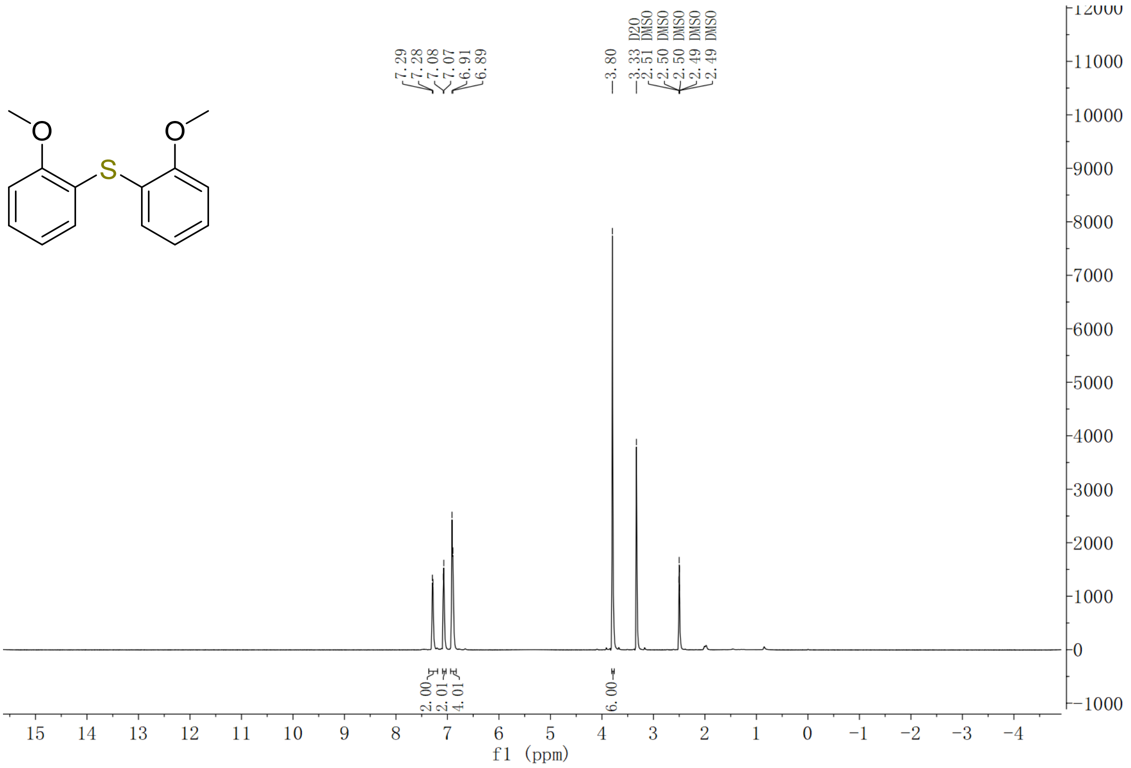

^{13}C NMR

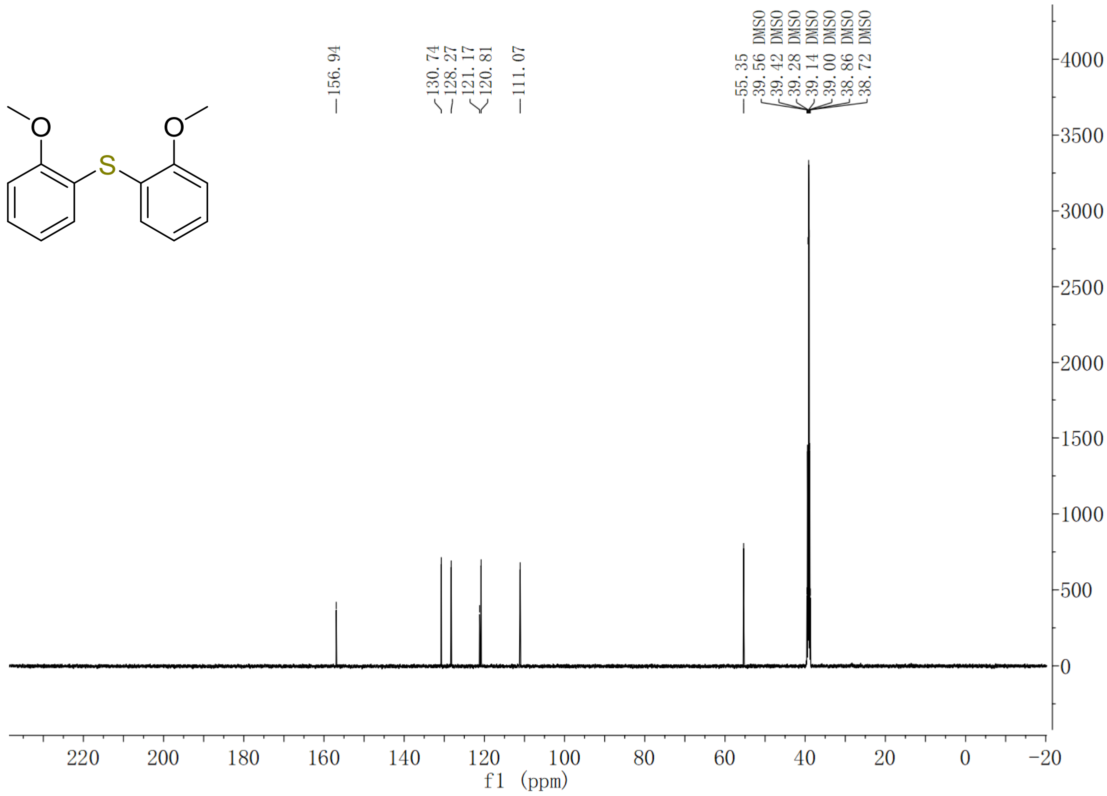

Bis(3-methoxyphenyl) sulfide（1c）

^{1}H NMR

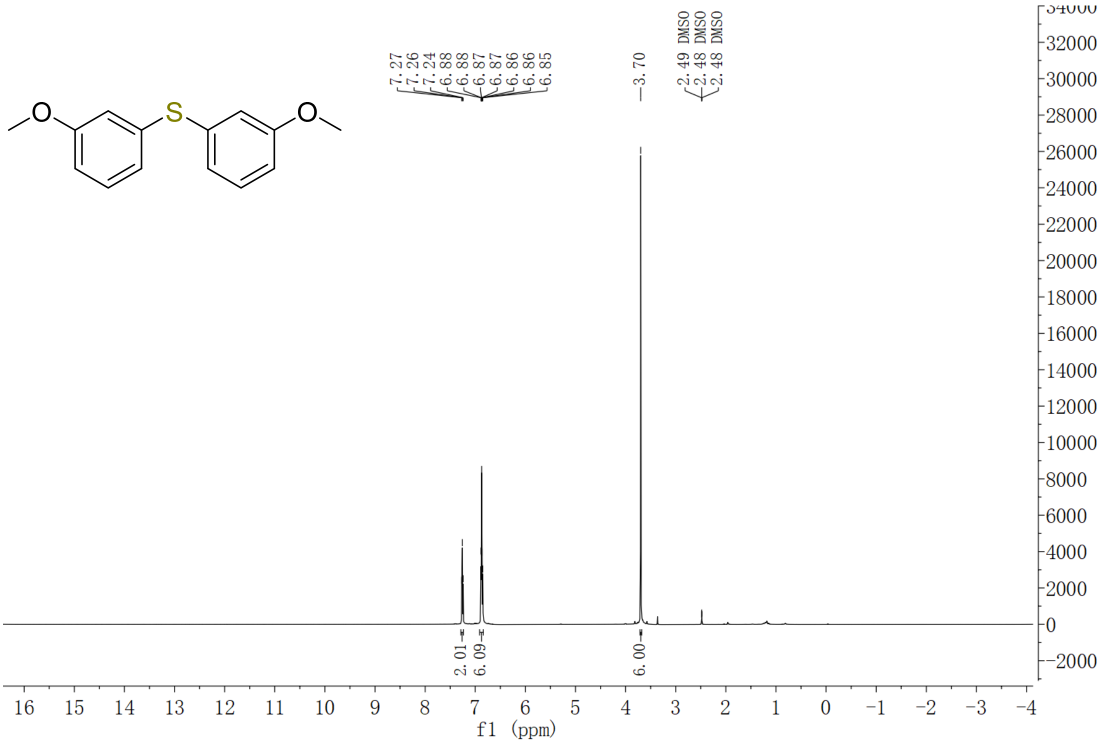

^{13}C NMR

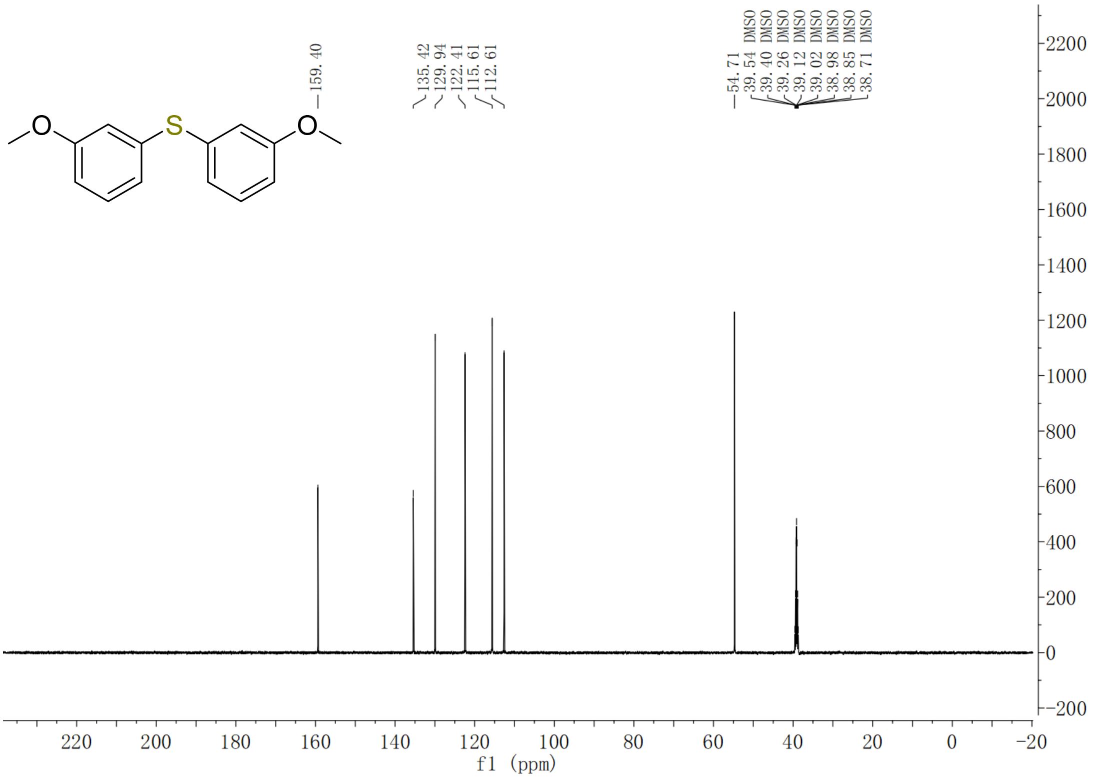

Bis(4-methoxyphenyl) sulfide（1d）

^{1}H NMR

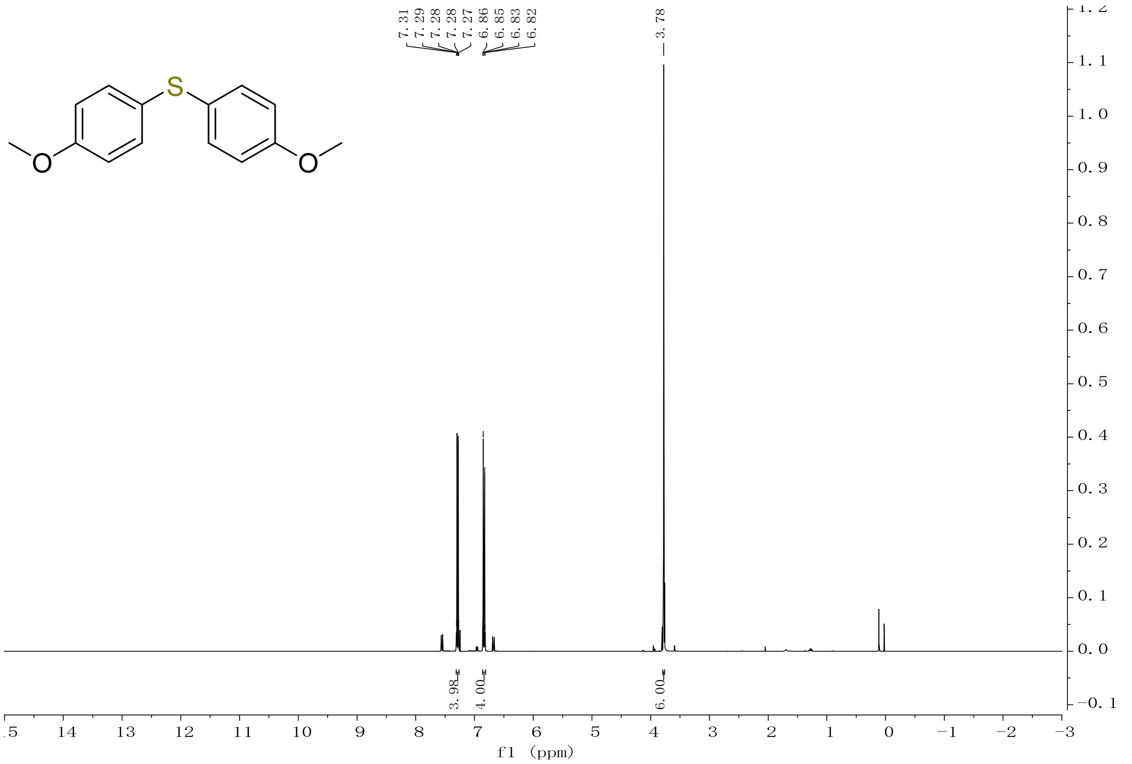

^{13}C NMR

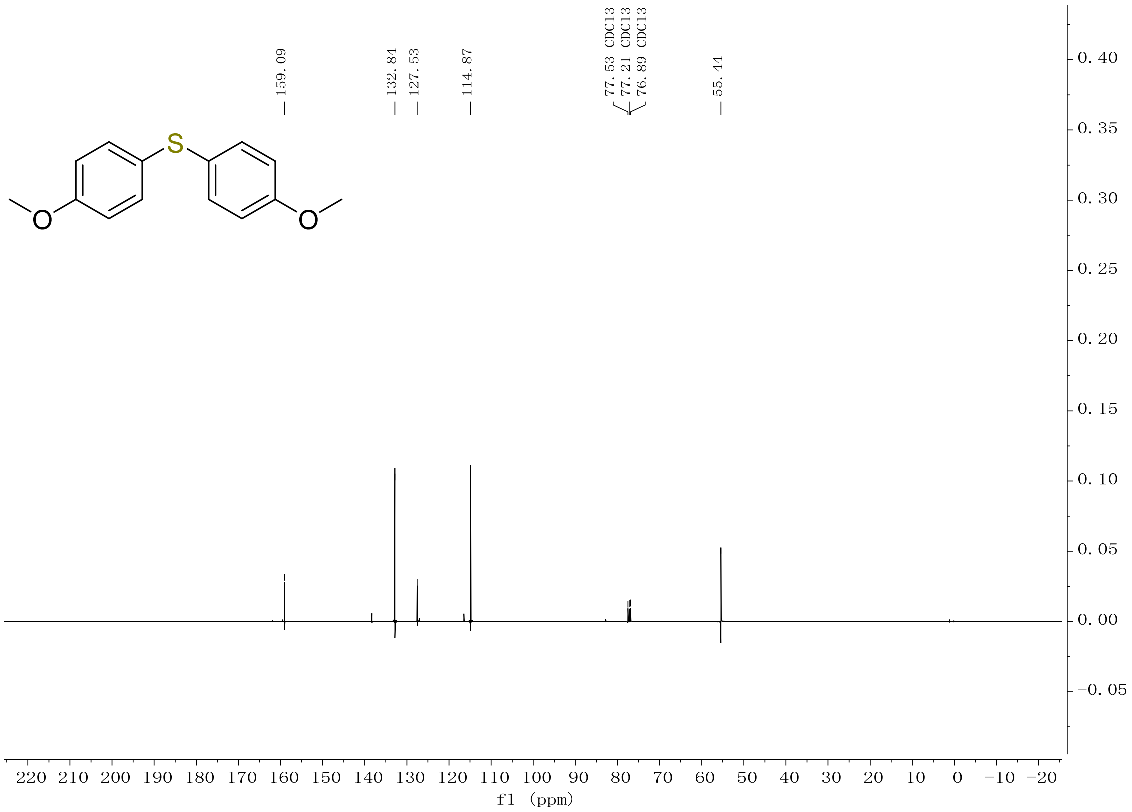

2,2´-Dimethyldiphenyl sulfide（1e）

^{1}H NMR

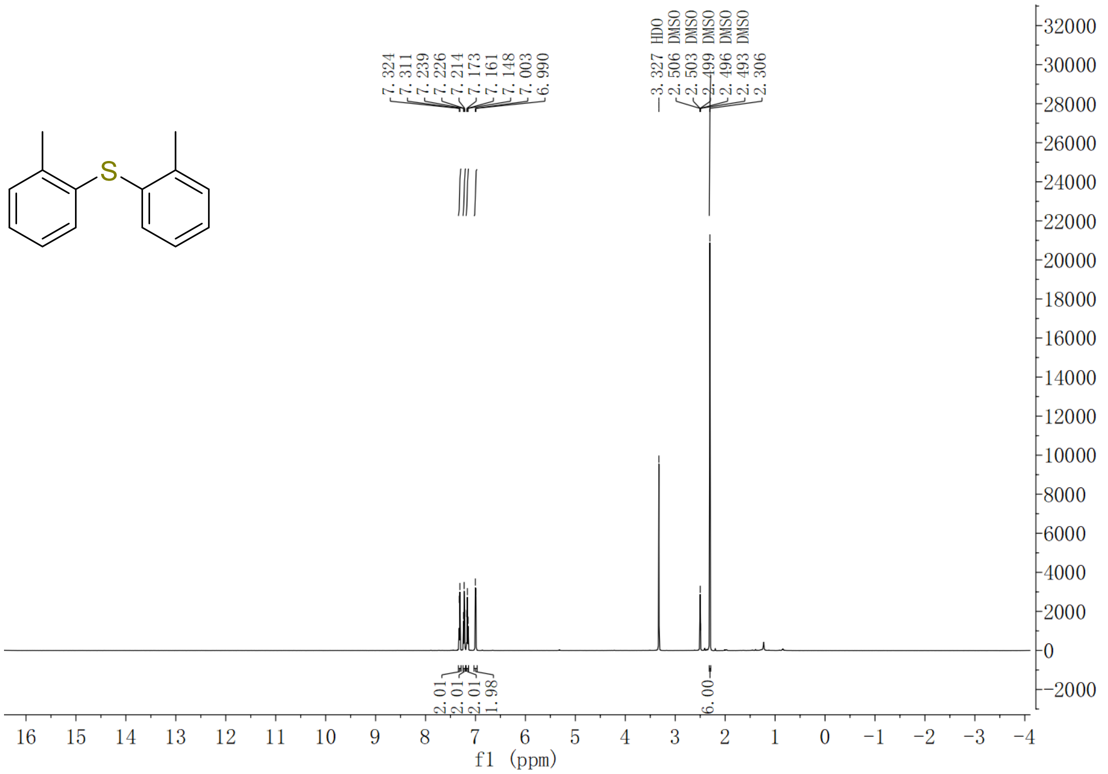

^{13}C NMR

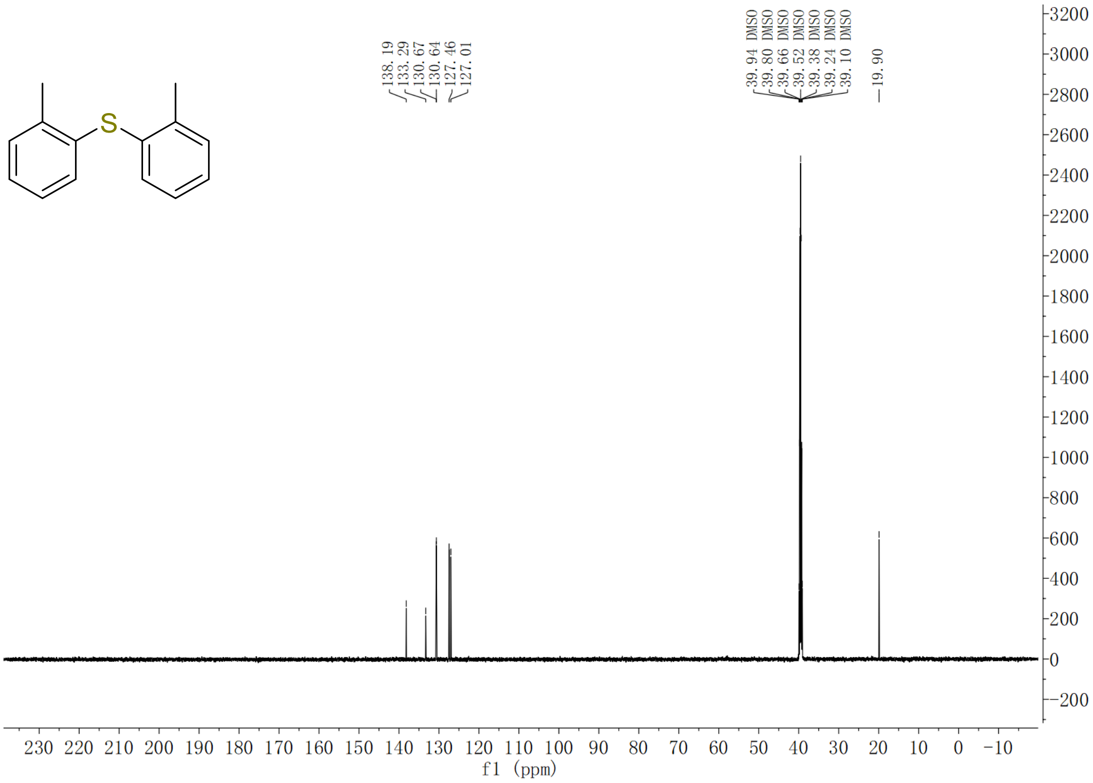

3,3´-Dimethyldiphenyl sulfide（1f）

^{1}H NMR

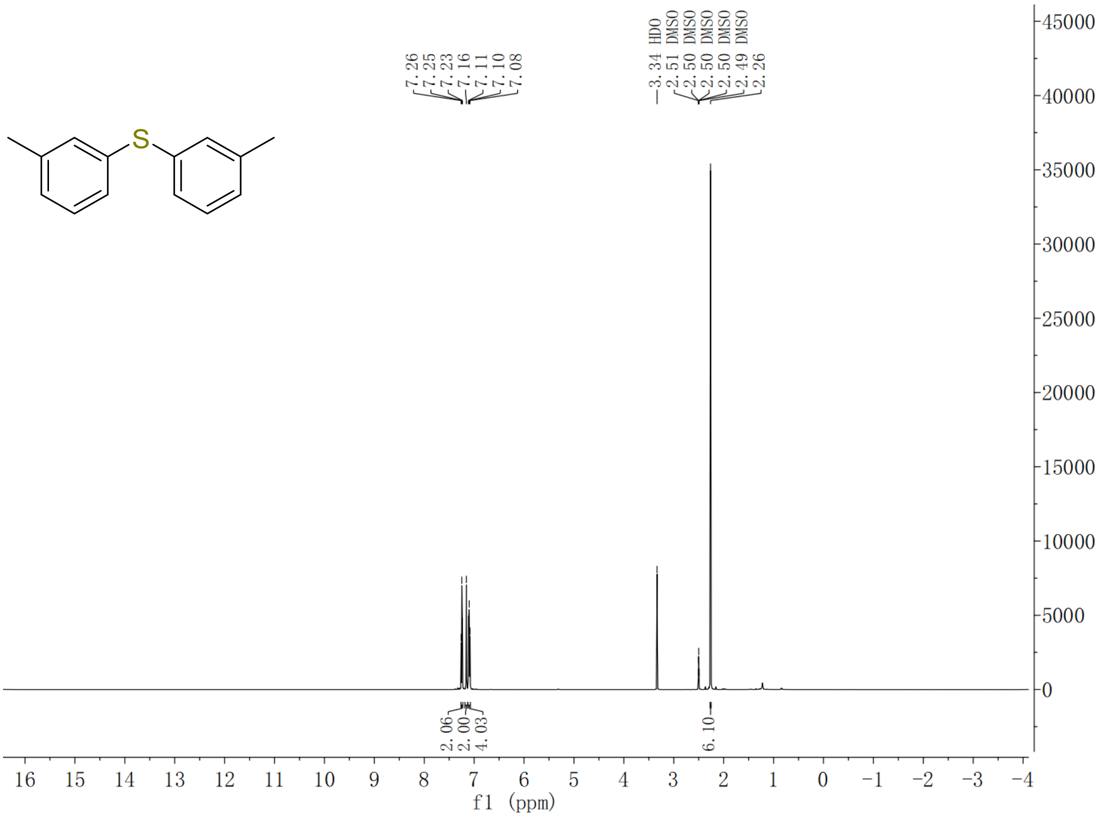

^{13}C NMR

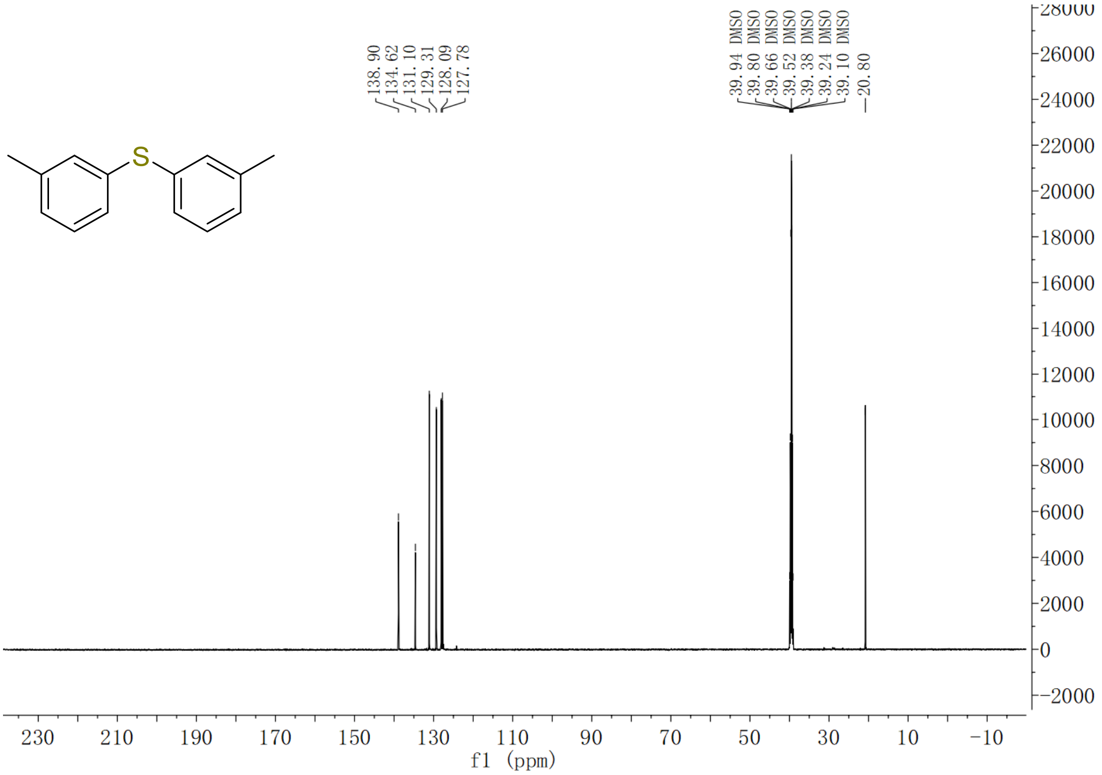

4,4´-Dimethyldiphenyl sulfide（1g）

^{1}H NMR

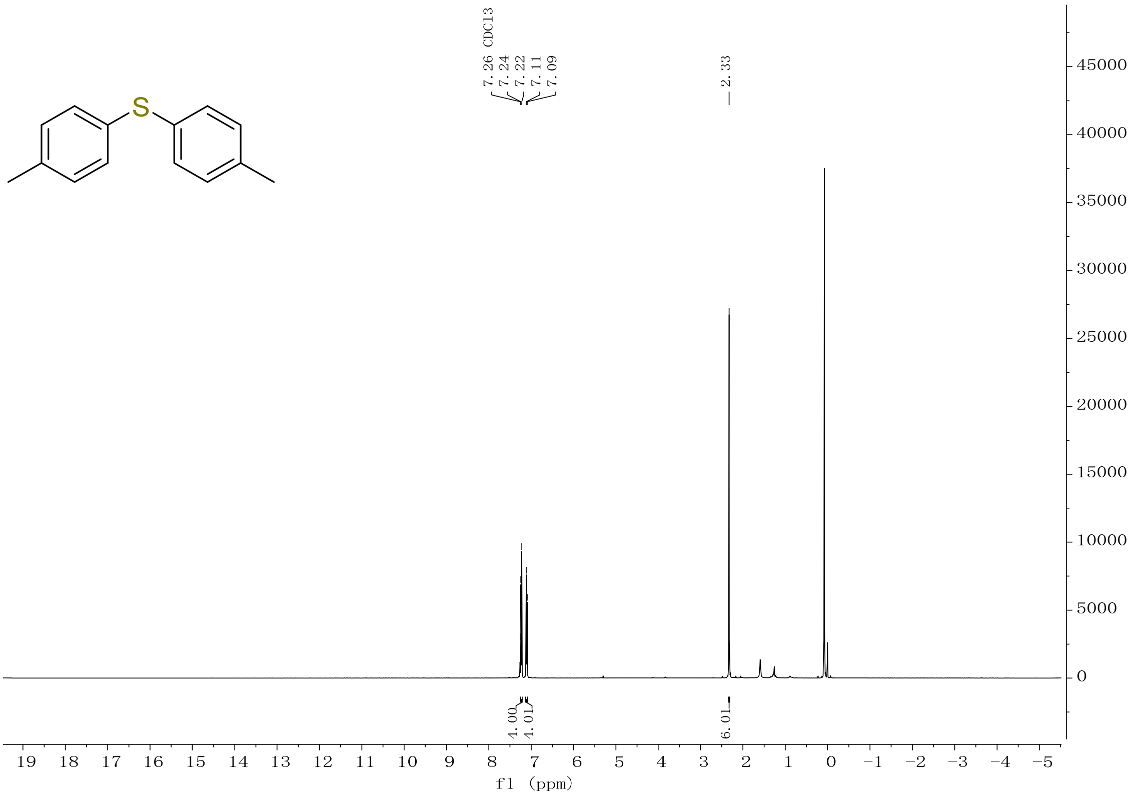

^{13}C NMR

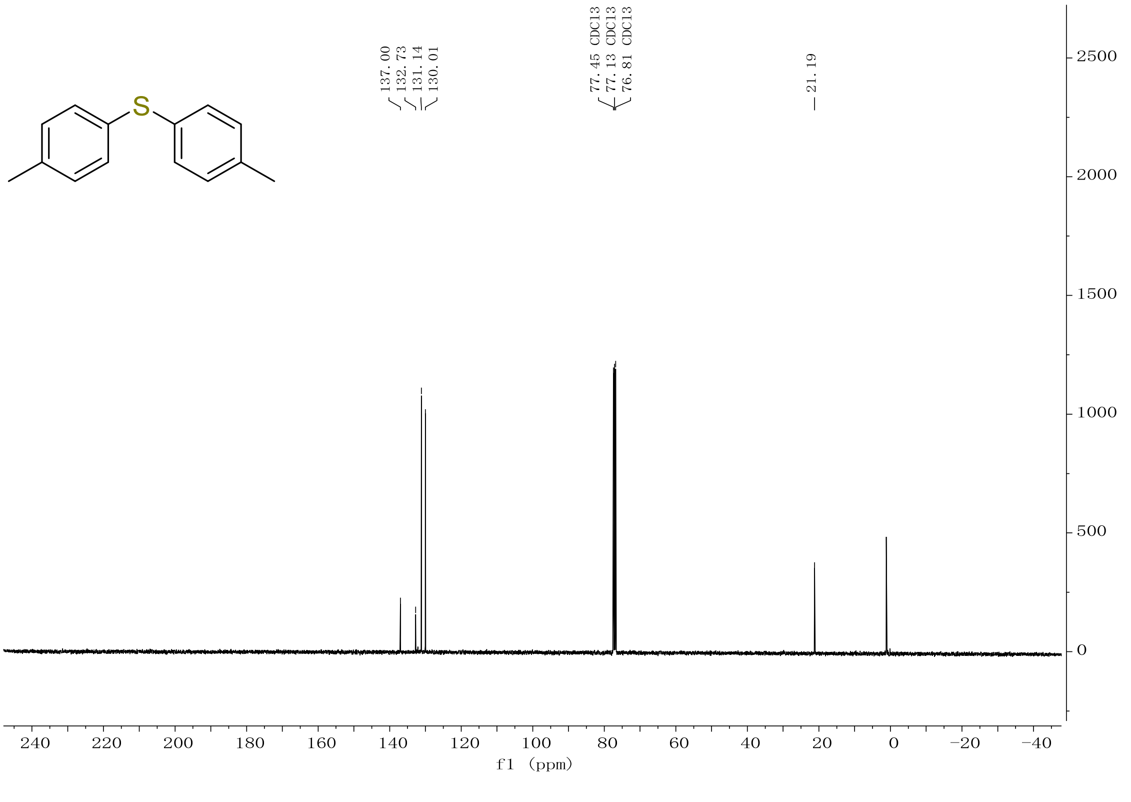

4,4´-Di-tert-butyldiphenyl sulfide（1h）

^{1}H NMR

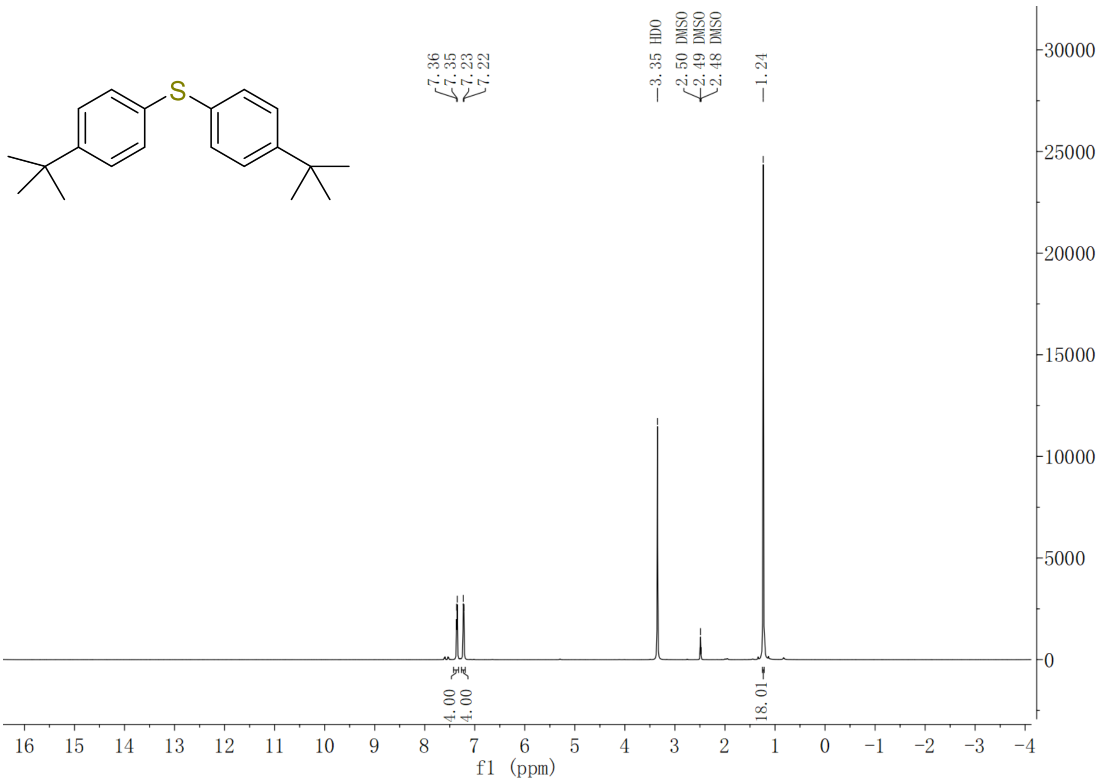

^{13}C NMR

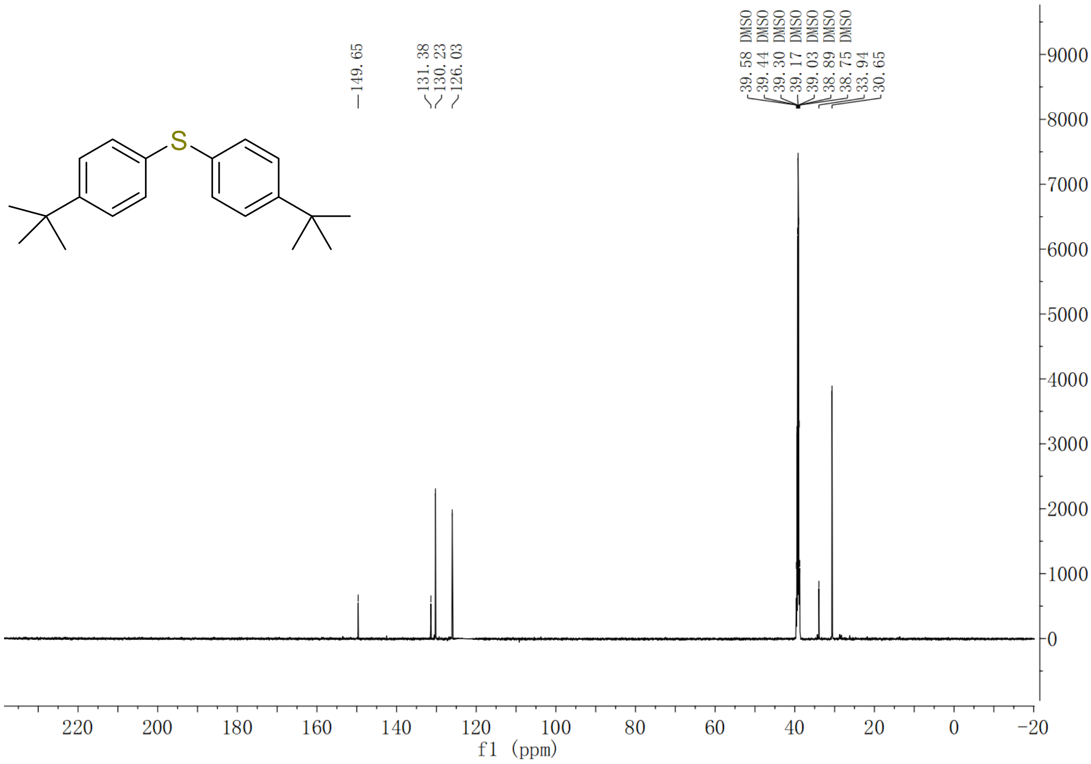

Bis(p-hydroxhphenyl) sulfide（1i）

^{1}H NMR

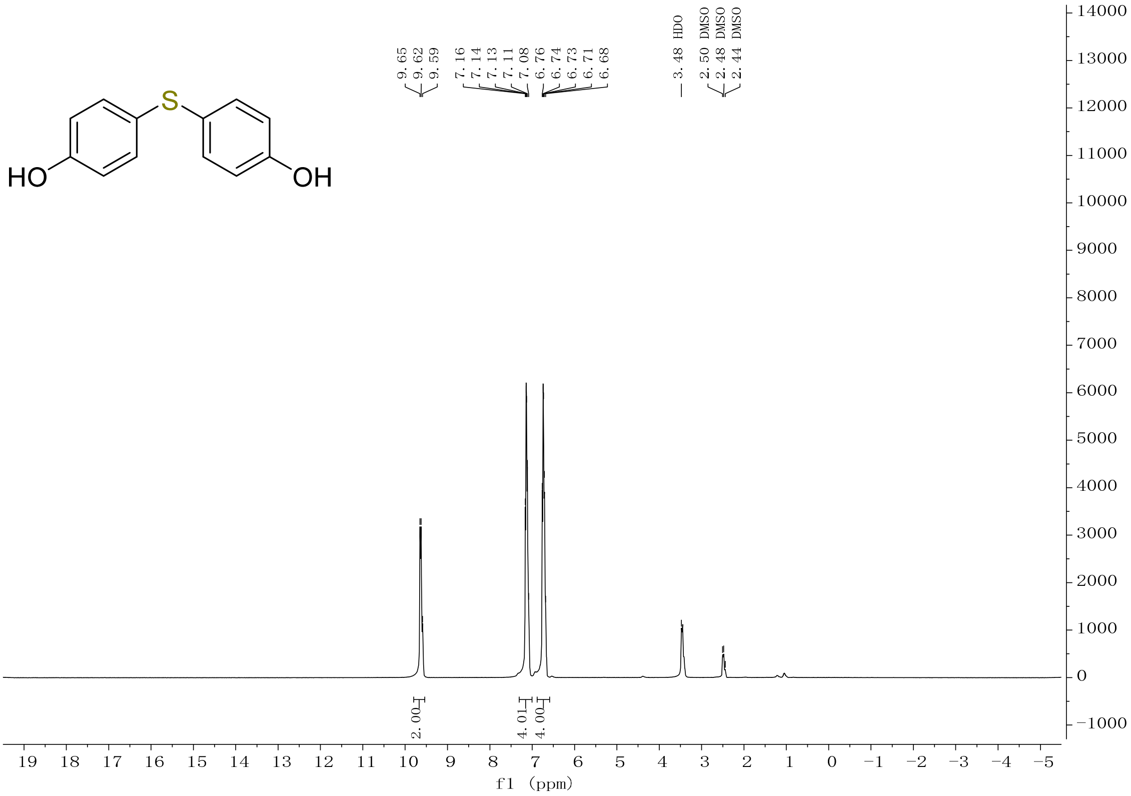

^{13}C NMR

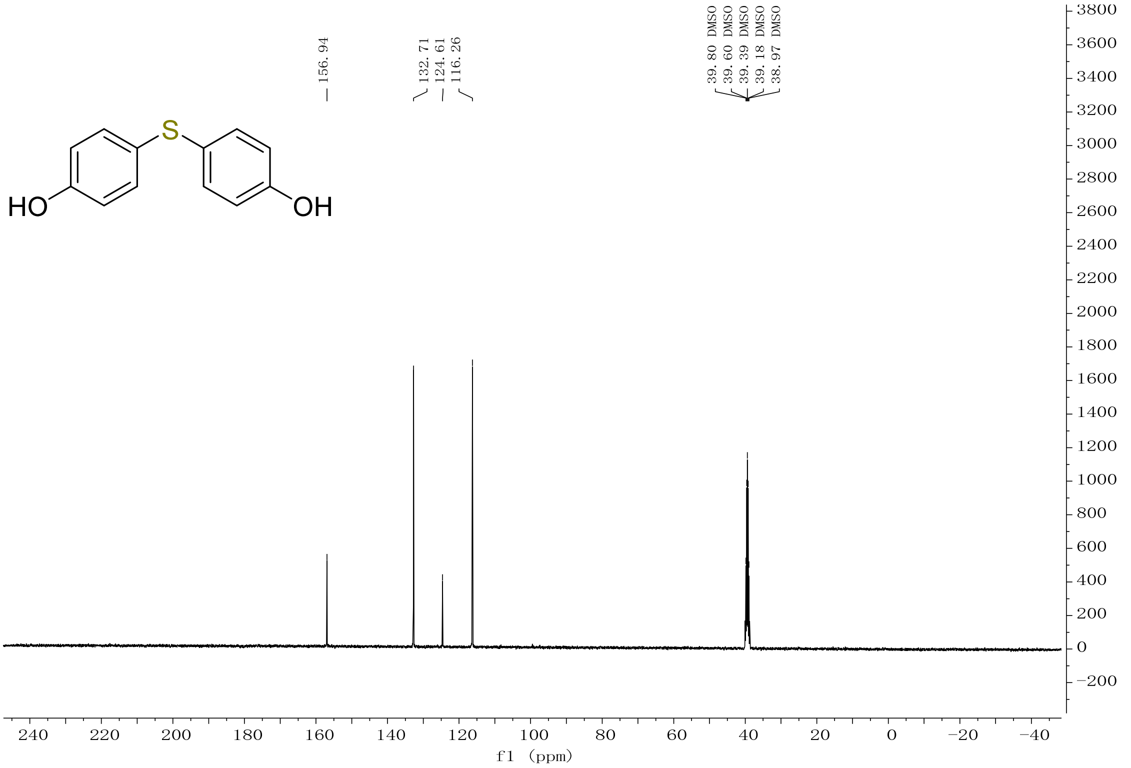

Bis(4-aminophenyl)sulphide（1j）

^{1}H NMR

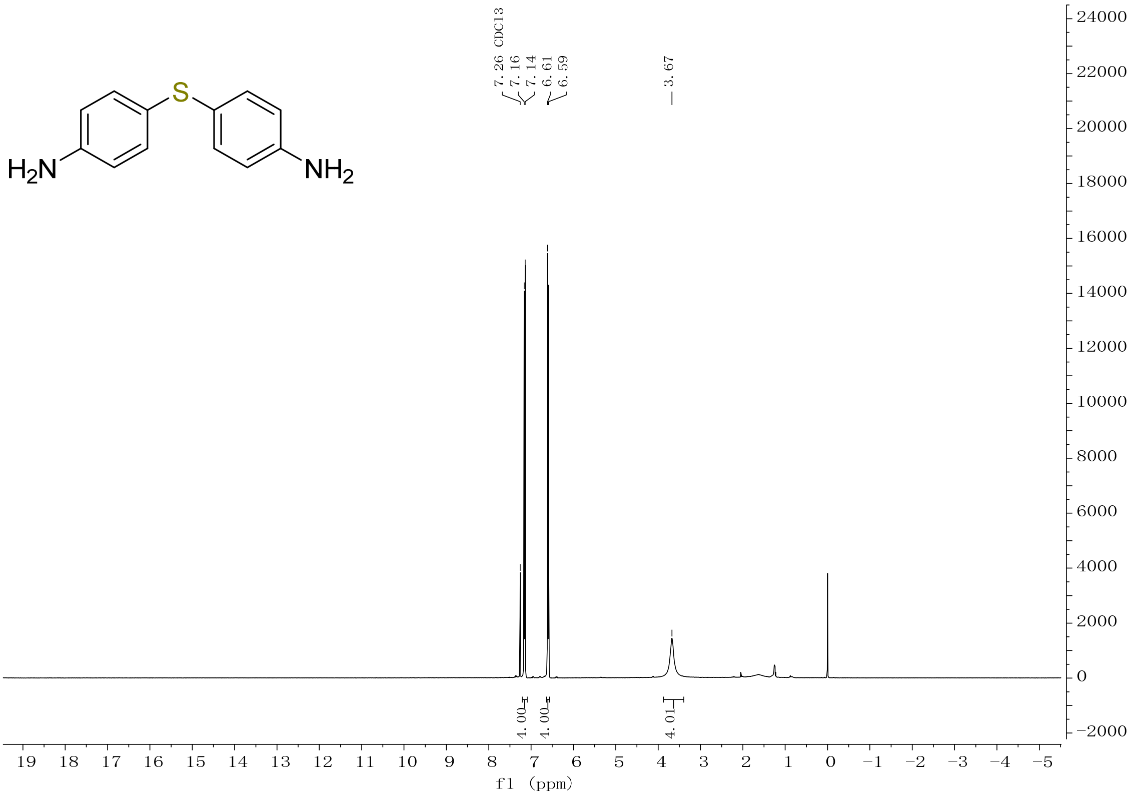

^{13}C NMR

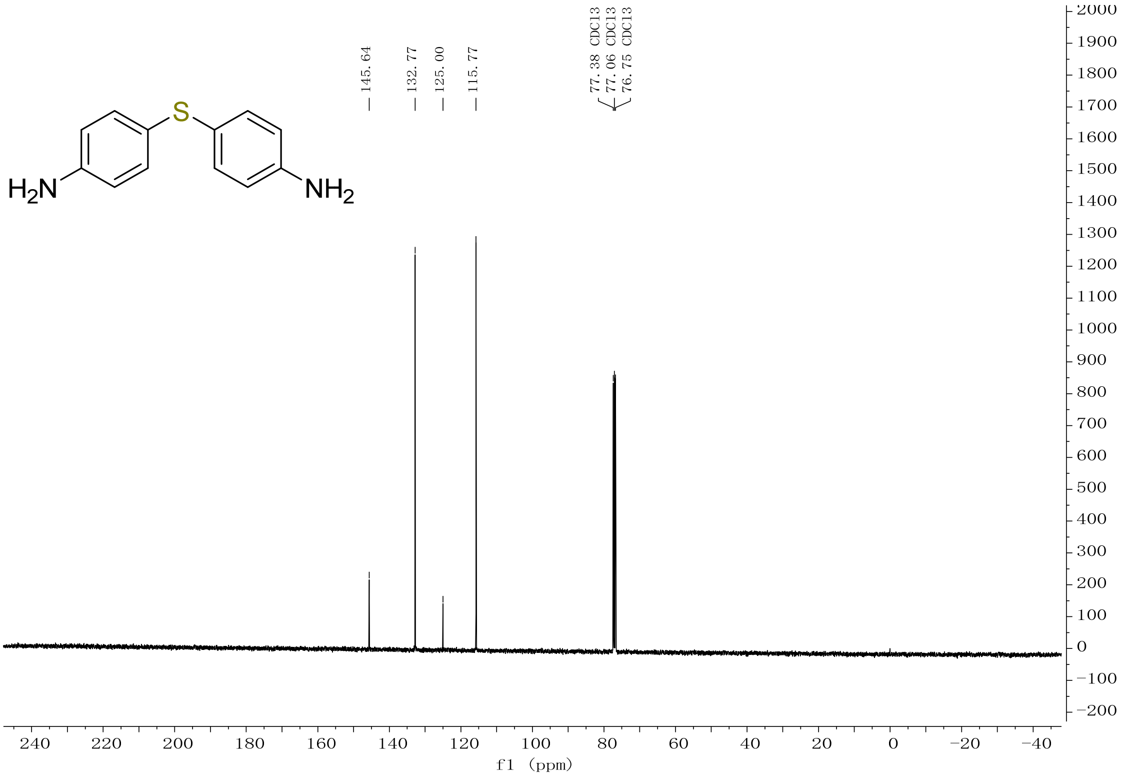

Bis(2-aminophenyl)sulphide（1k）

^{1}H NMR

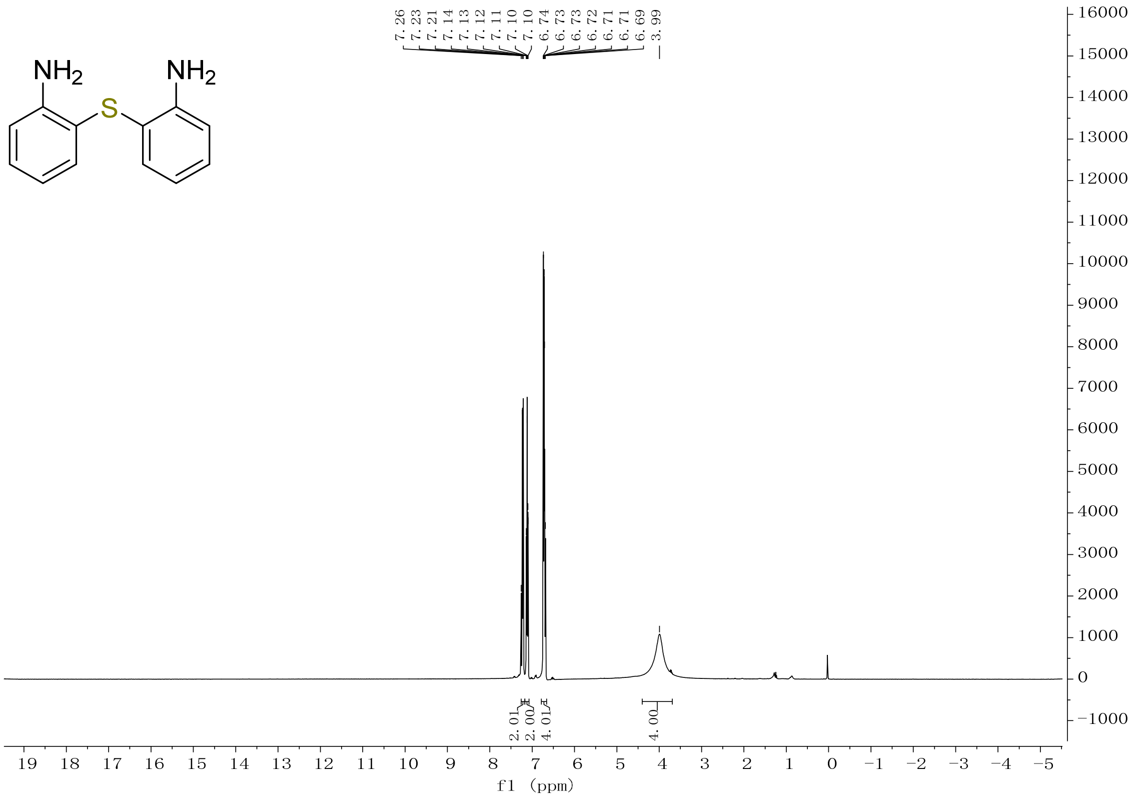

^{13}C NMR

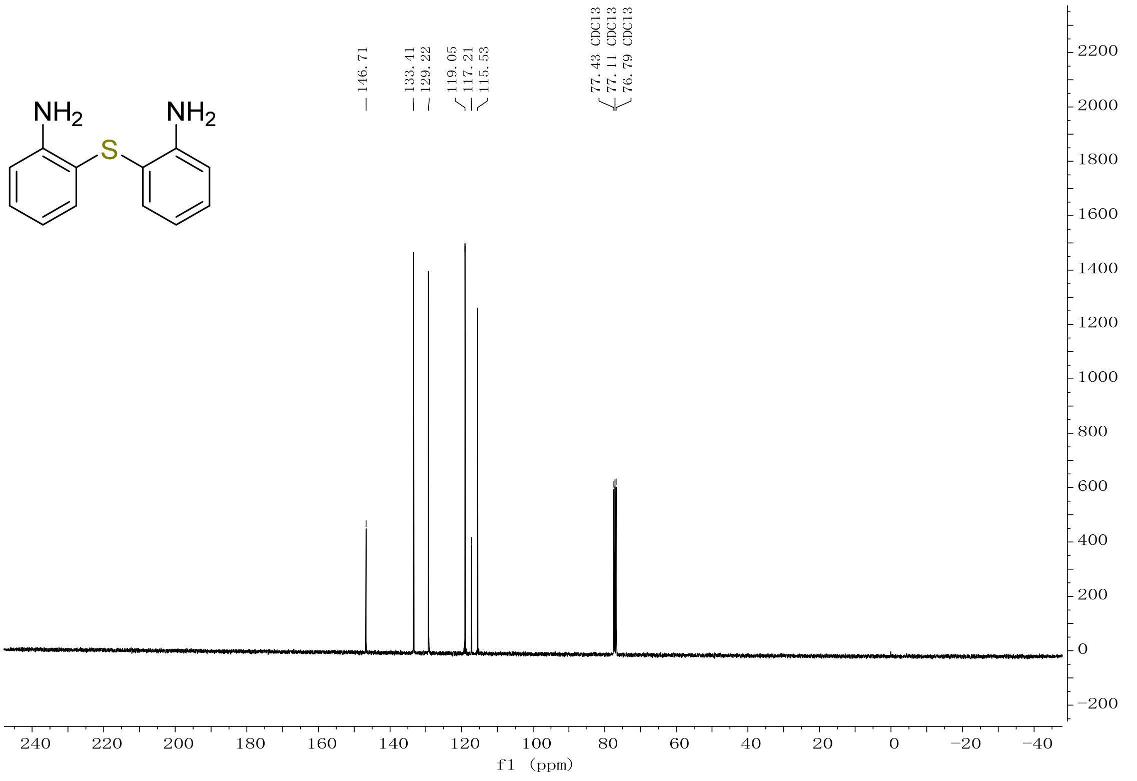

Bis(2-fluorophenyl) sulfide（1l）

^{1}H NMR

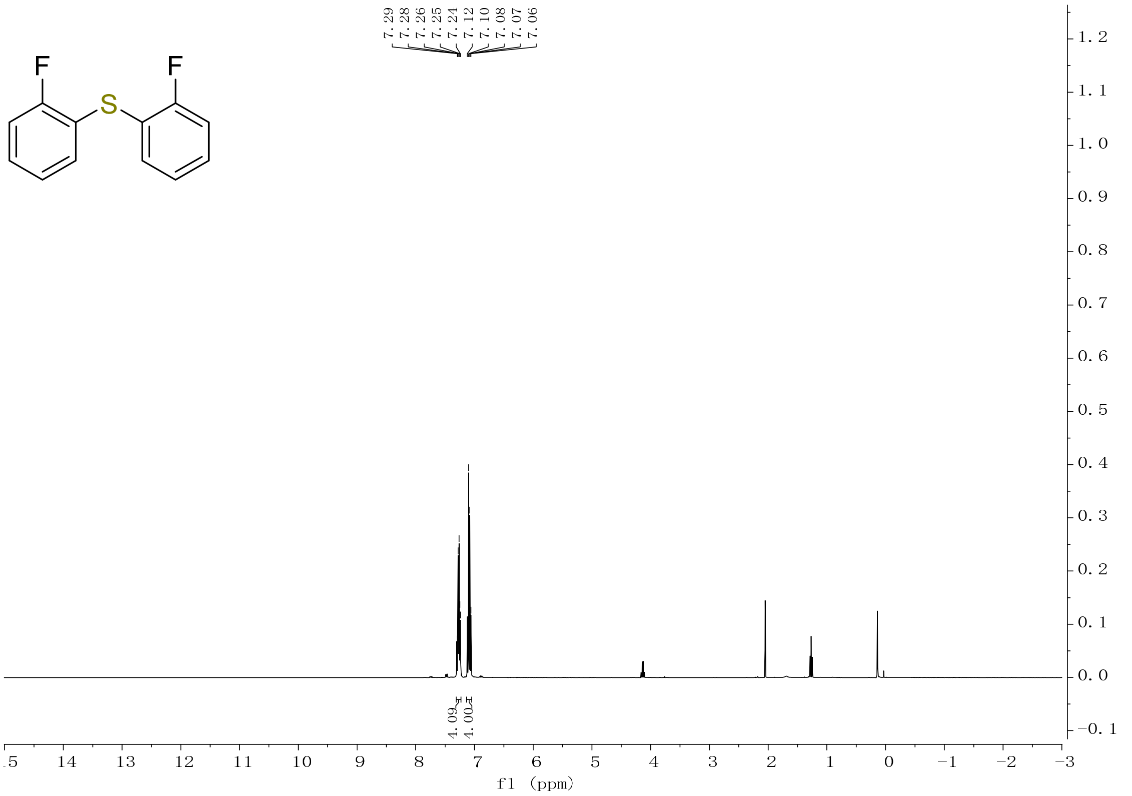

^{13}C NMR

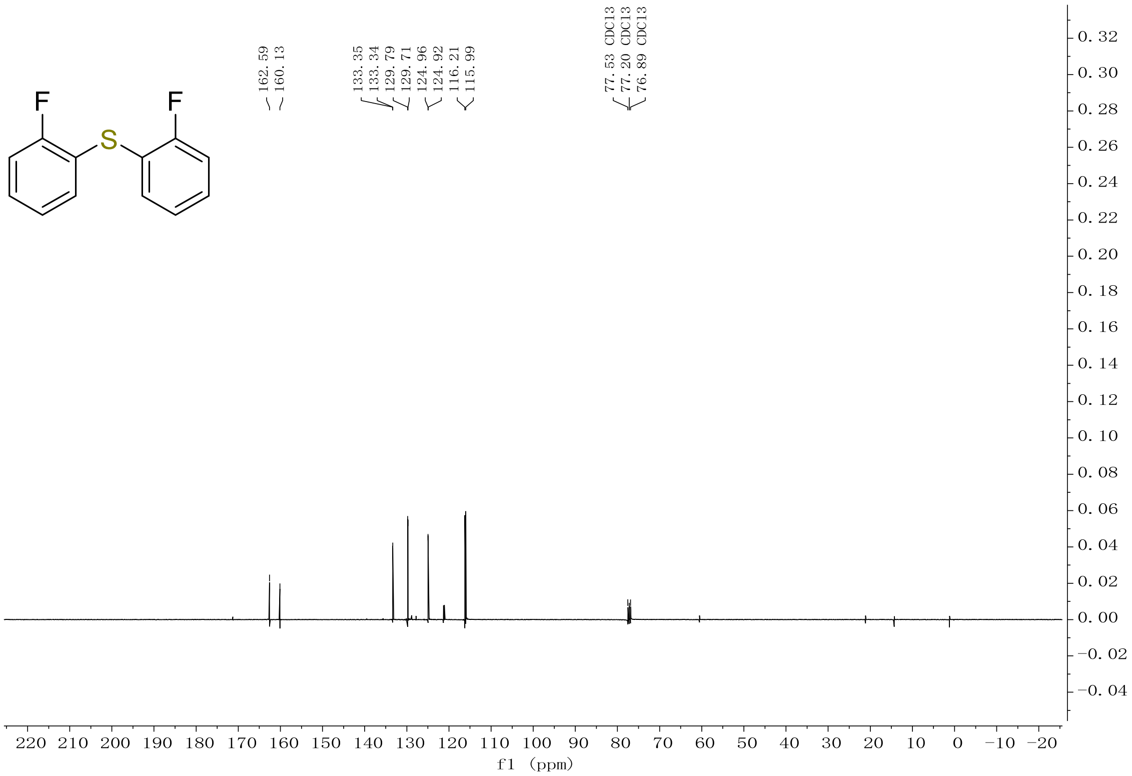

^{19}F NMR

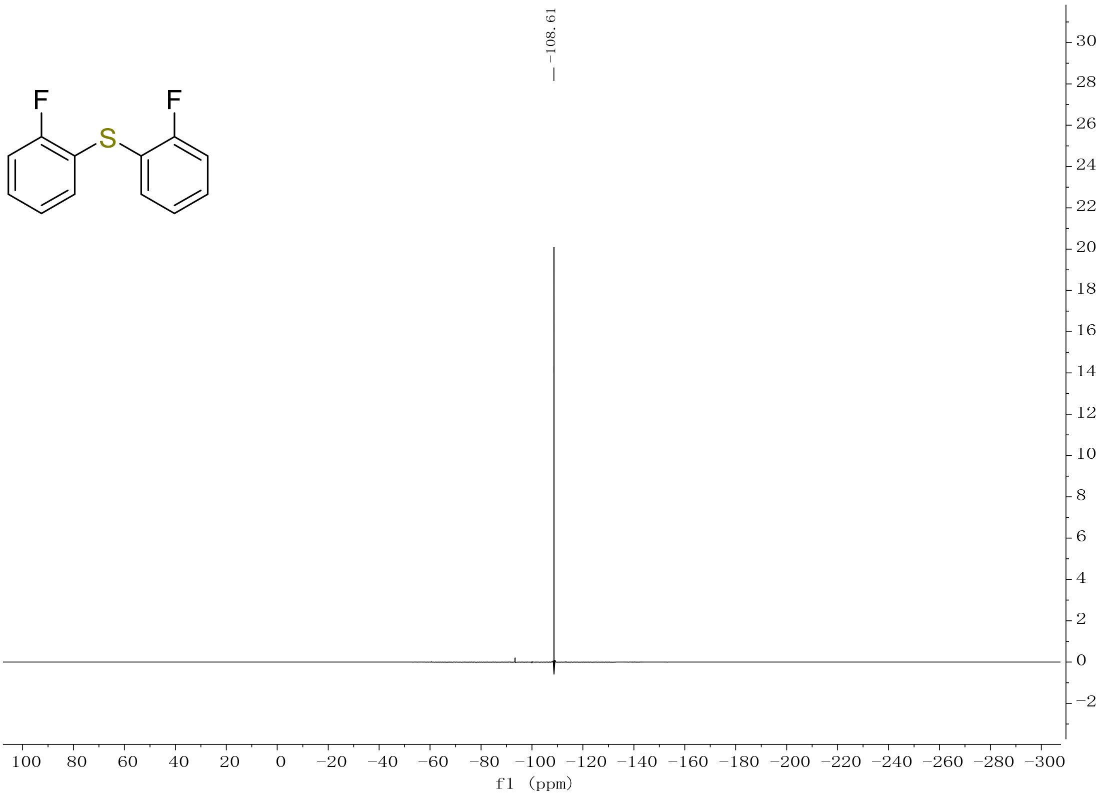

F
S
F
−108.61
30
28
26
24
22
20
18
16
14
12
10
8
6
4
2
0
−2
100 80 60 40 20 0 −20 −40 −60 −80 −100 −120 −140 −160 −180 −200 −220 −240 −260 −280 −300
f1 (ppm)

Bis(4-chlorophenyl) sulfide（1m）

^{1}H NMR

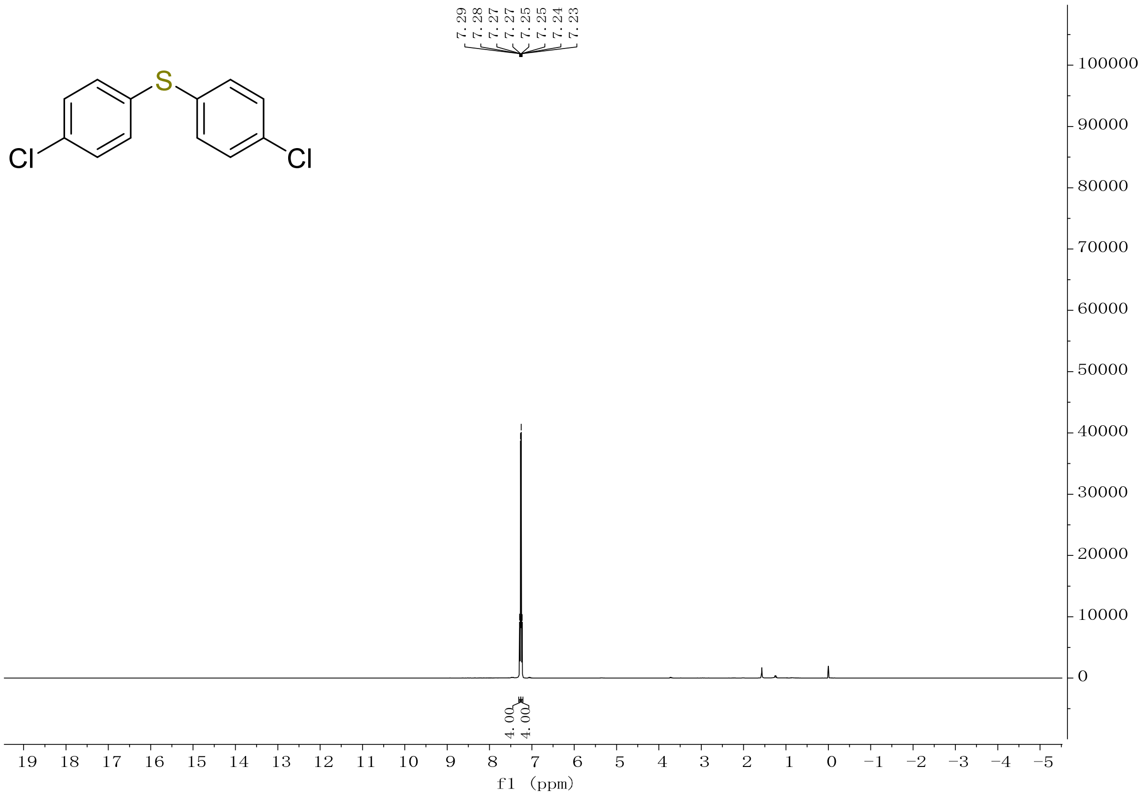

^{13}C NMR

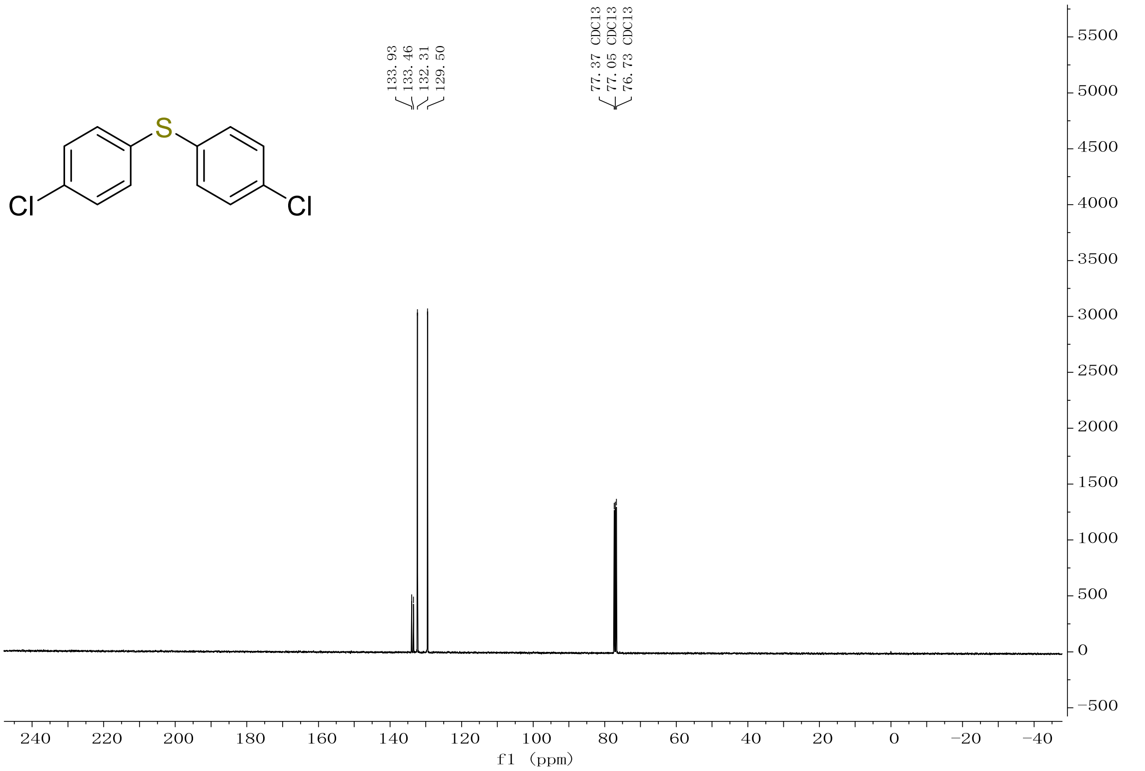

Bis(4-bromophenyl) sulphide（1n）

^{1}H NMR

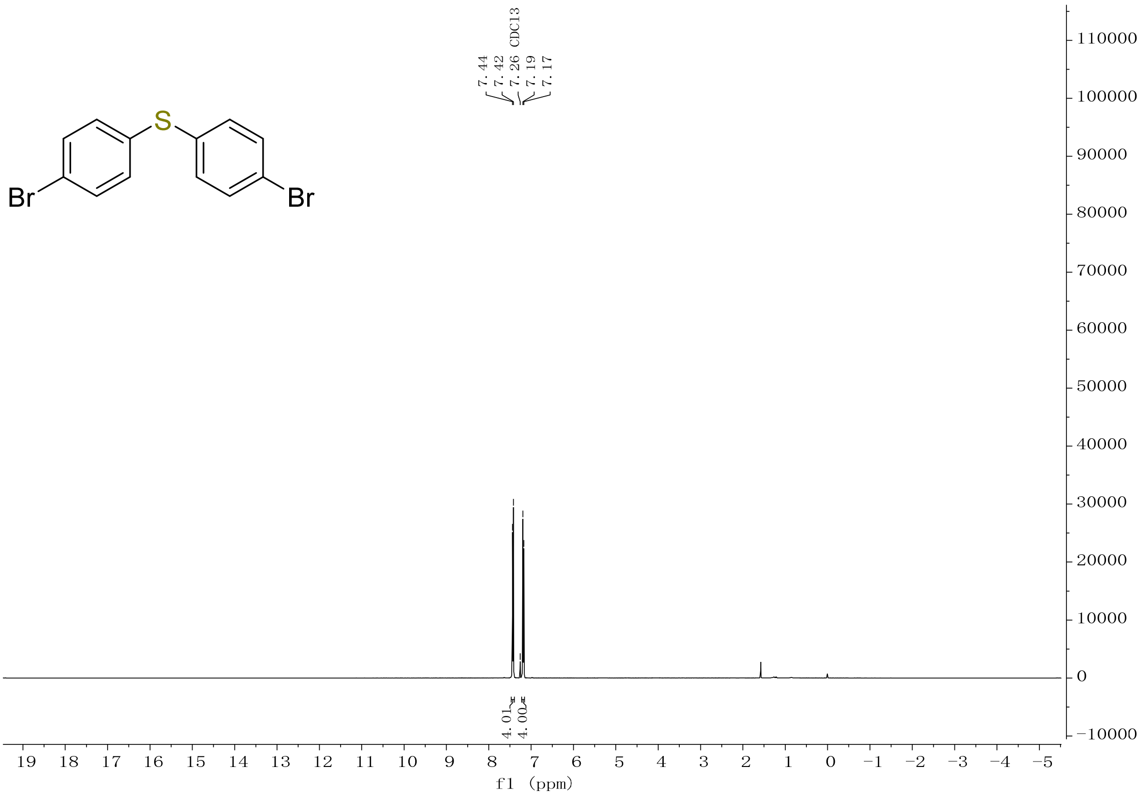

^{13}C NMR

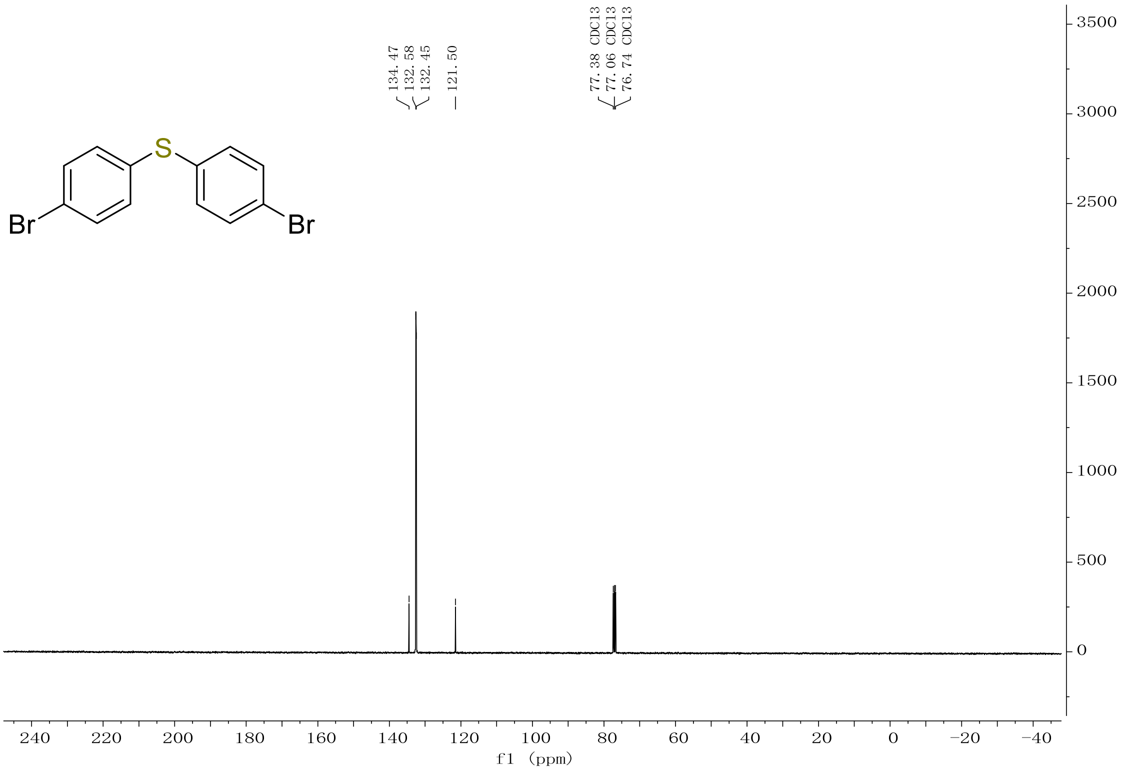

4,4´-Dicyanodiphenyl sulfide（1o）

^{1}H NMR

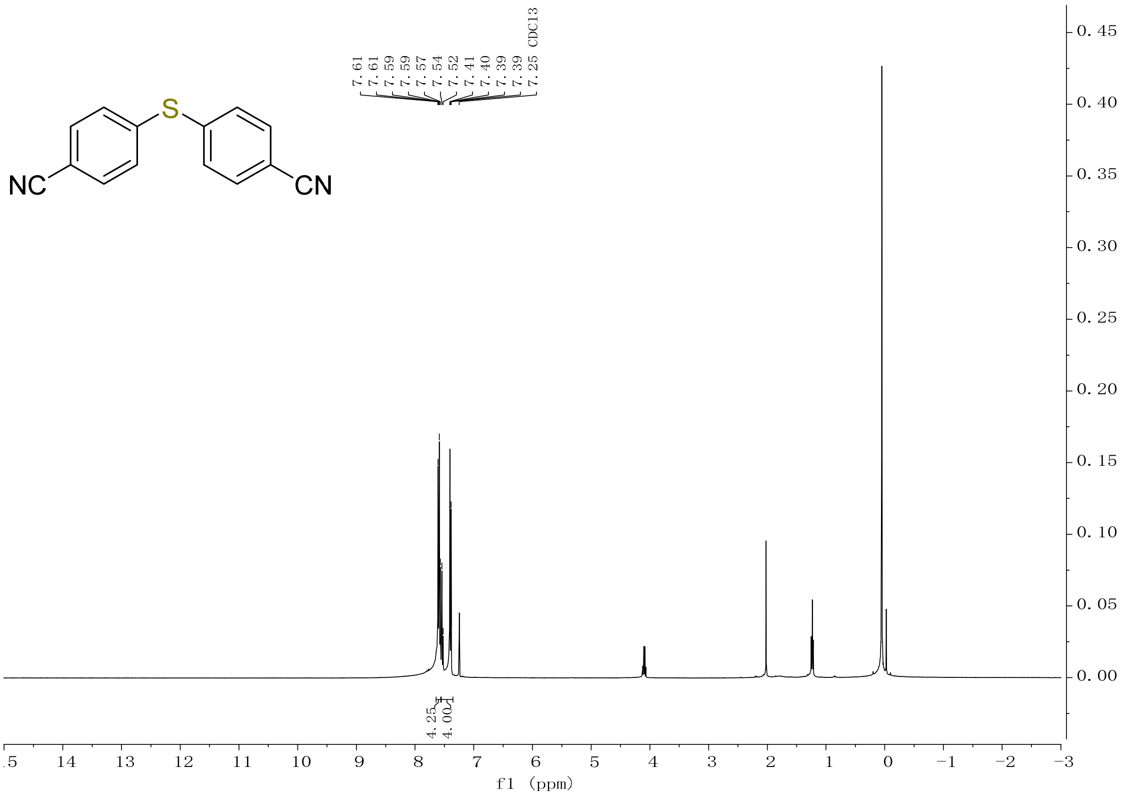

^{13}C NMR

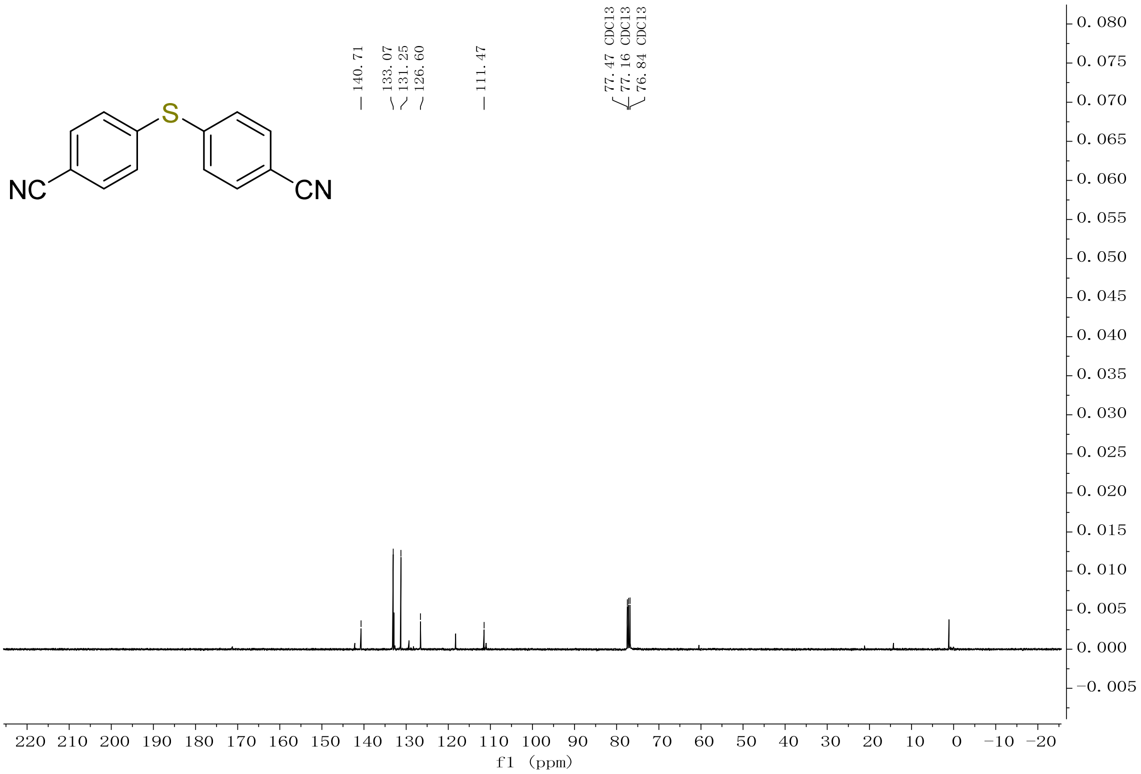

Bis(4-(trifluoromethyl)phenyl)sulfane（1p）

^{1}H NMR

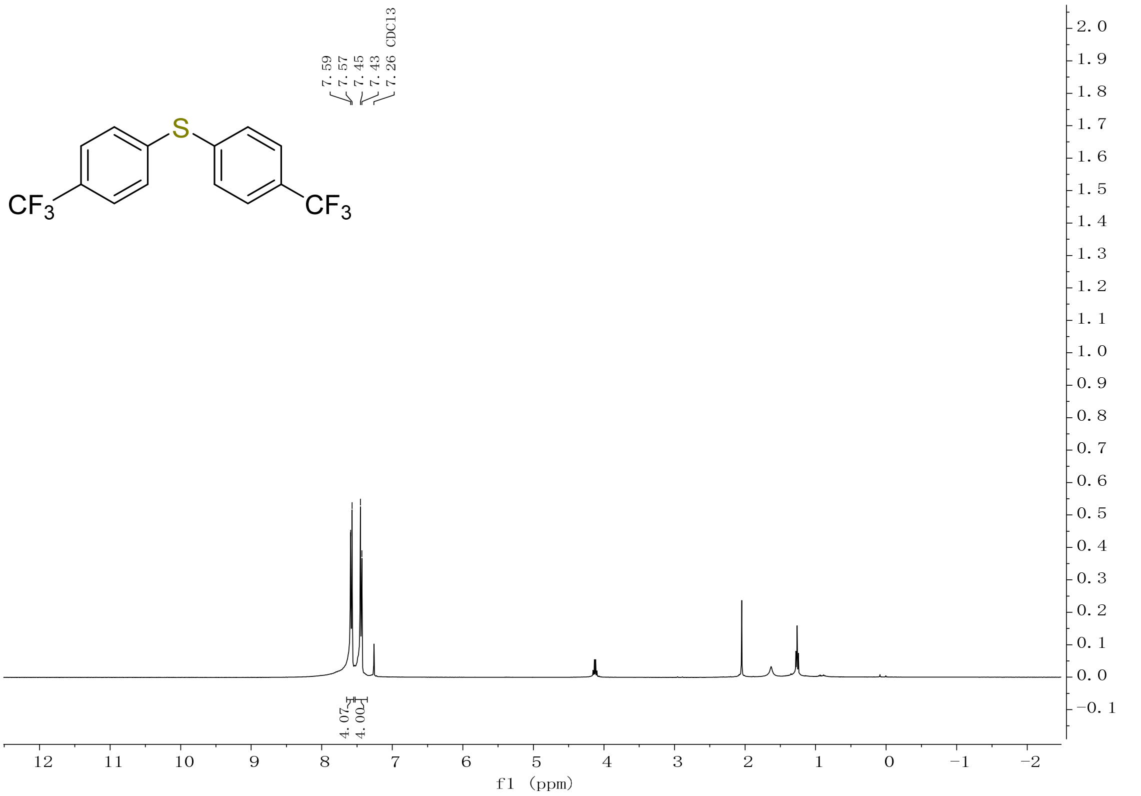

^{13}C NMR

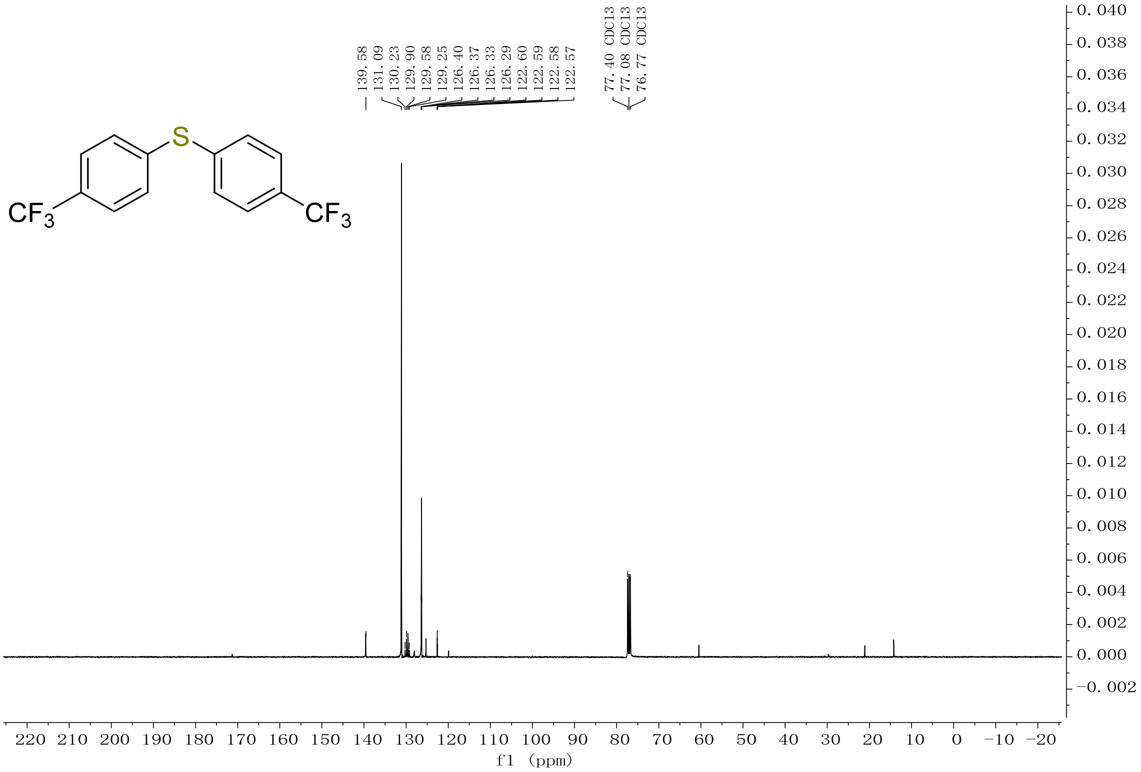

^{19}F NMR

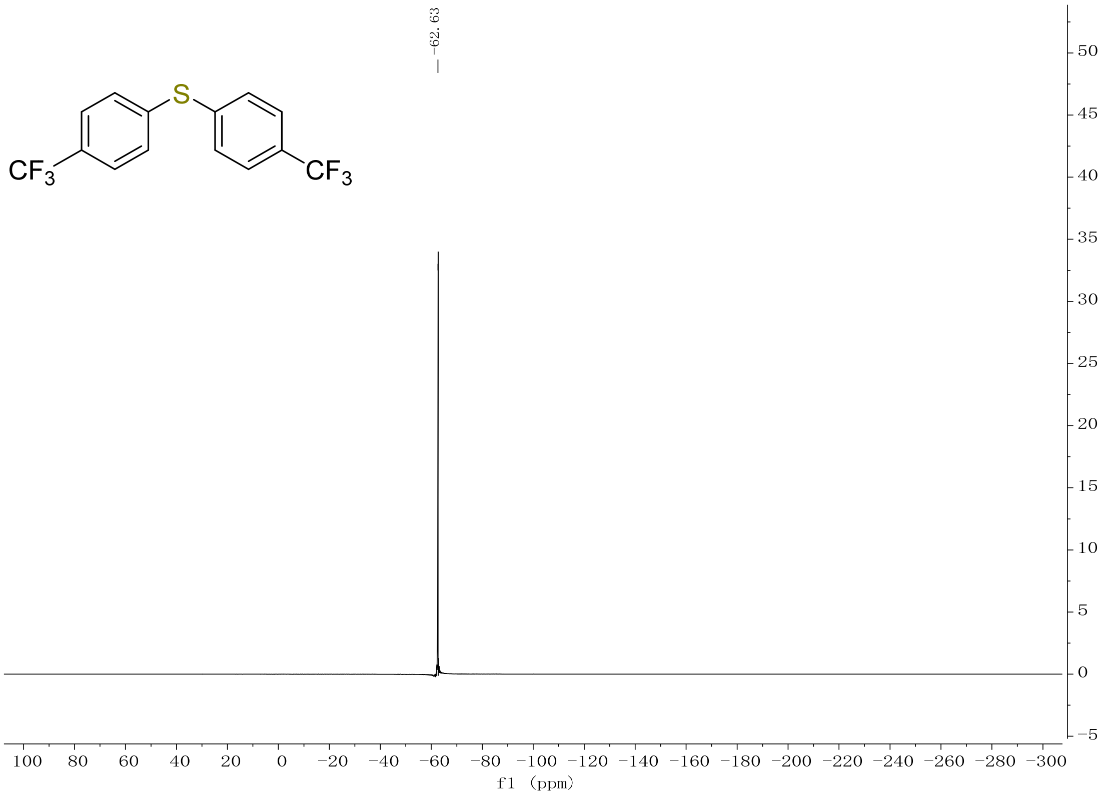

4,4′-Dinitrodiphenyl sulfide（1q）

^{1}H NMR

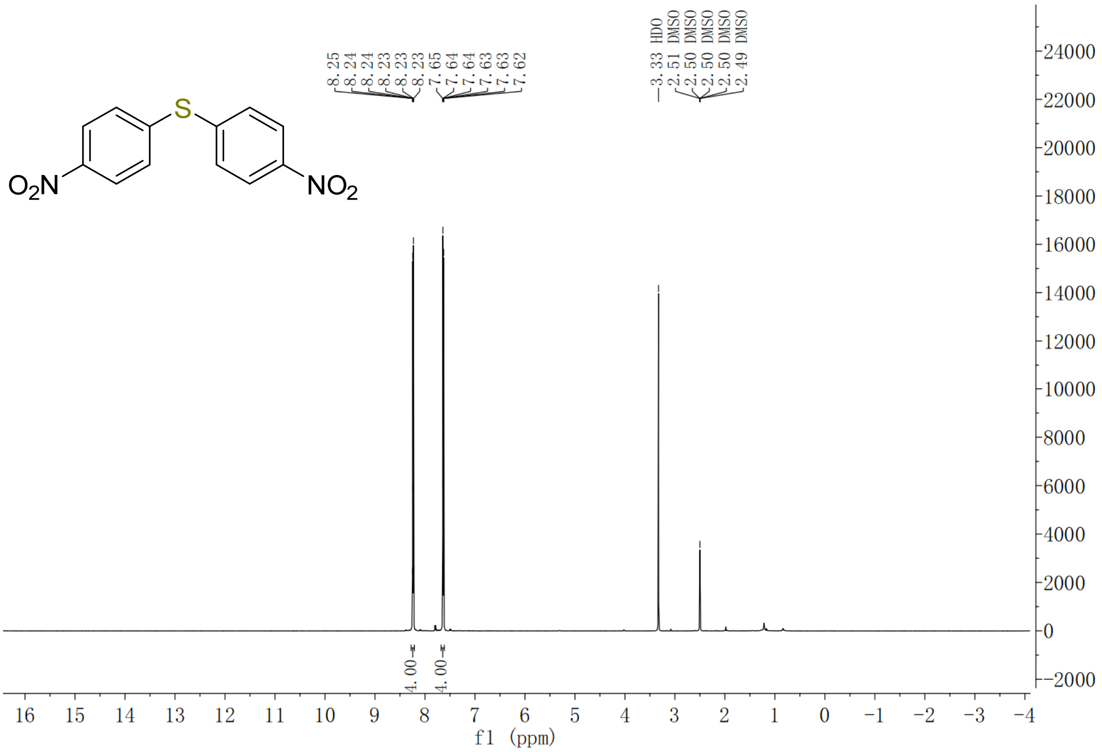

^{13}C NMR

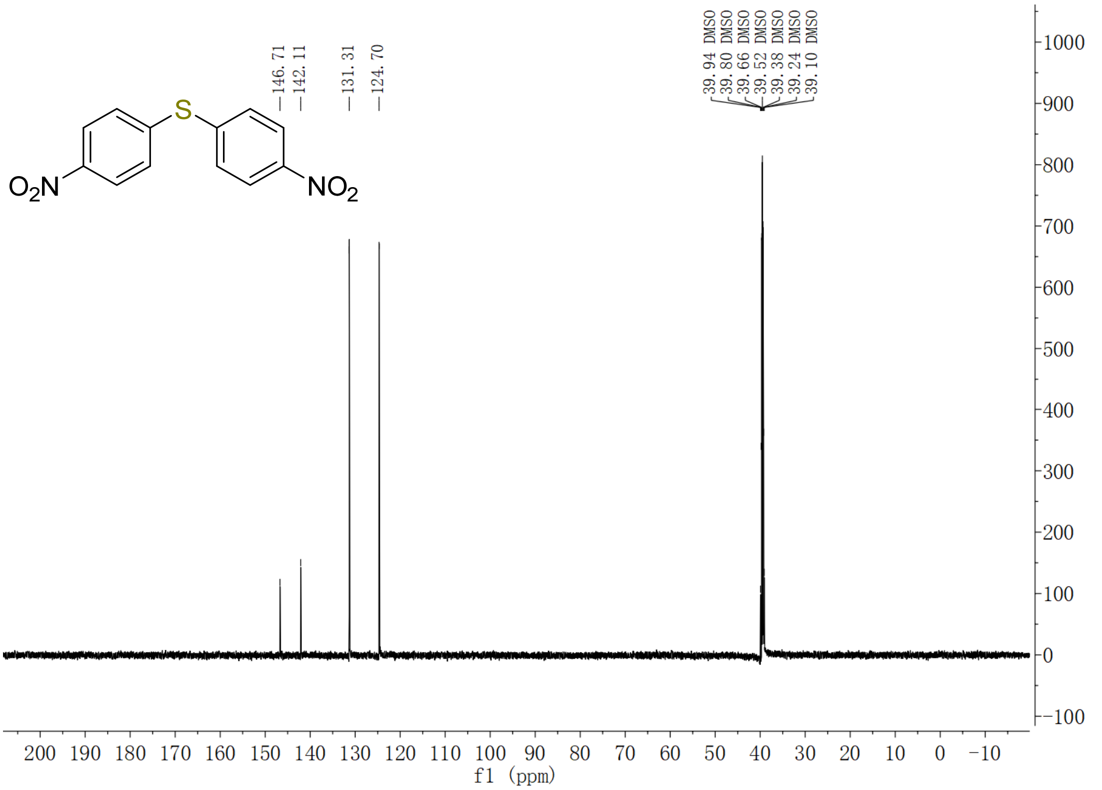

Bis(3,5-dimethylphenyl) sulfide（1r）

^{1}H NMR

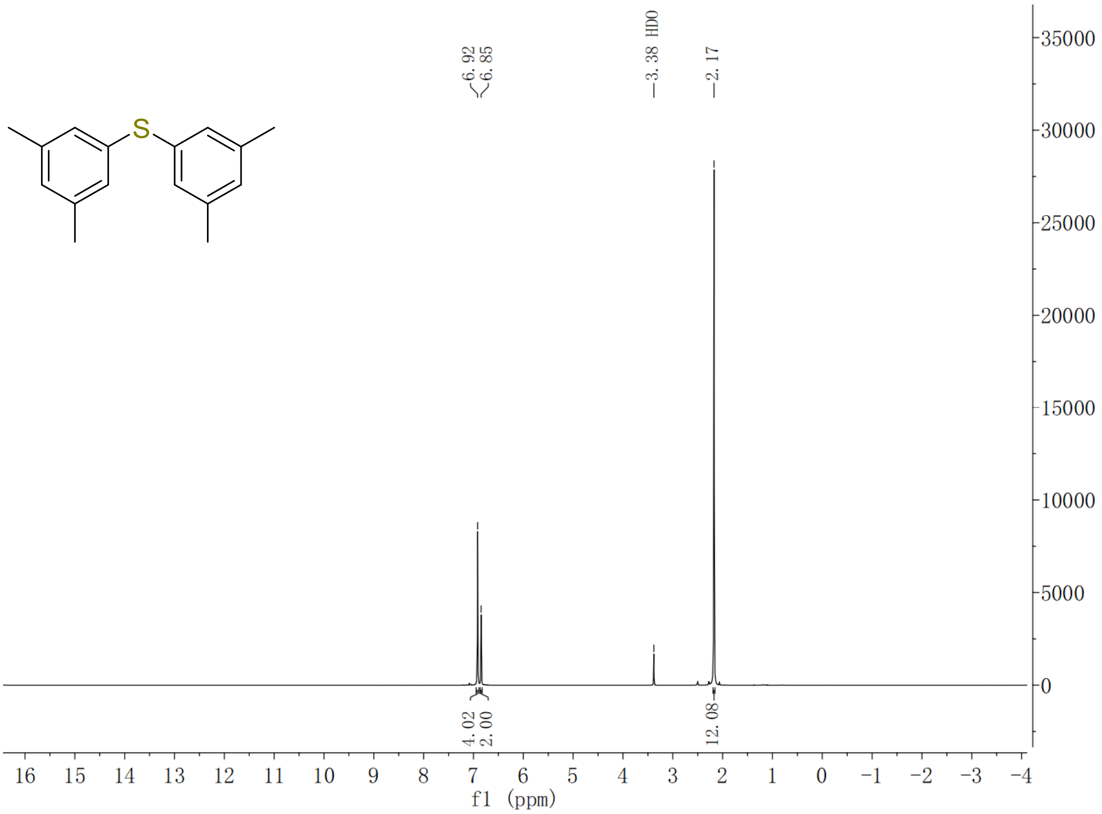

^{13}C NMR

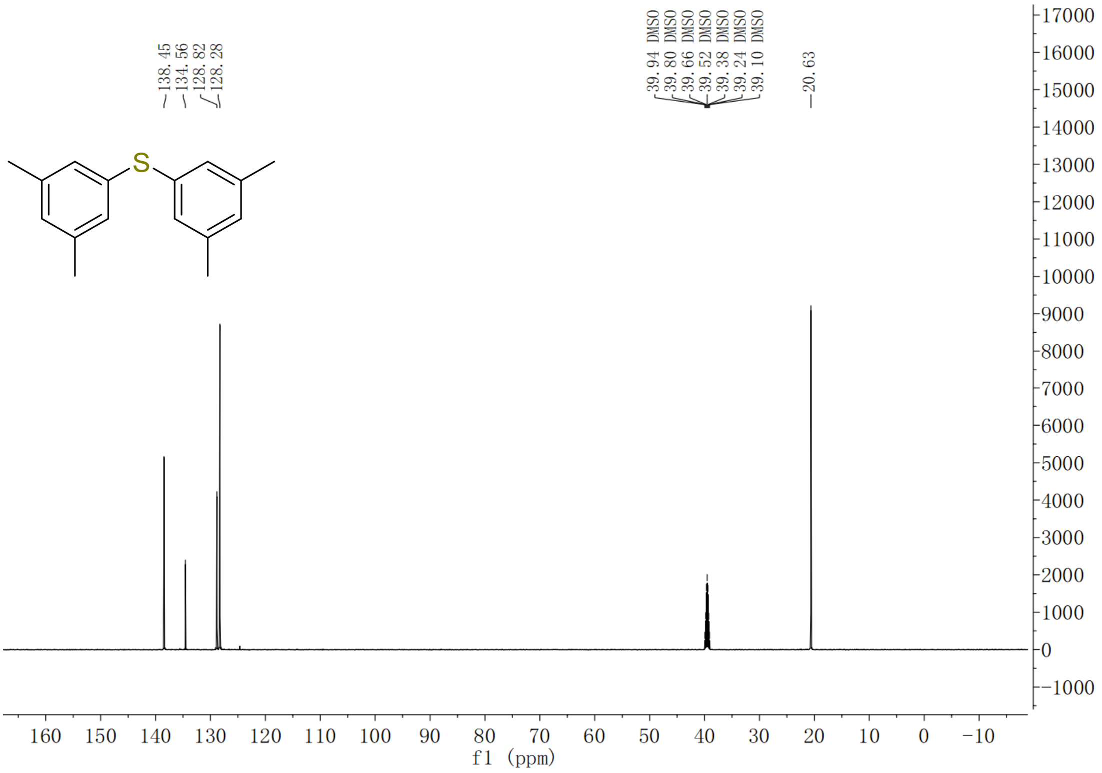

Bis(2-hydroxy-5-chlorophenyl) sulfide（1s）

^{1}H NMR

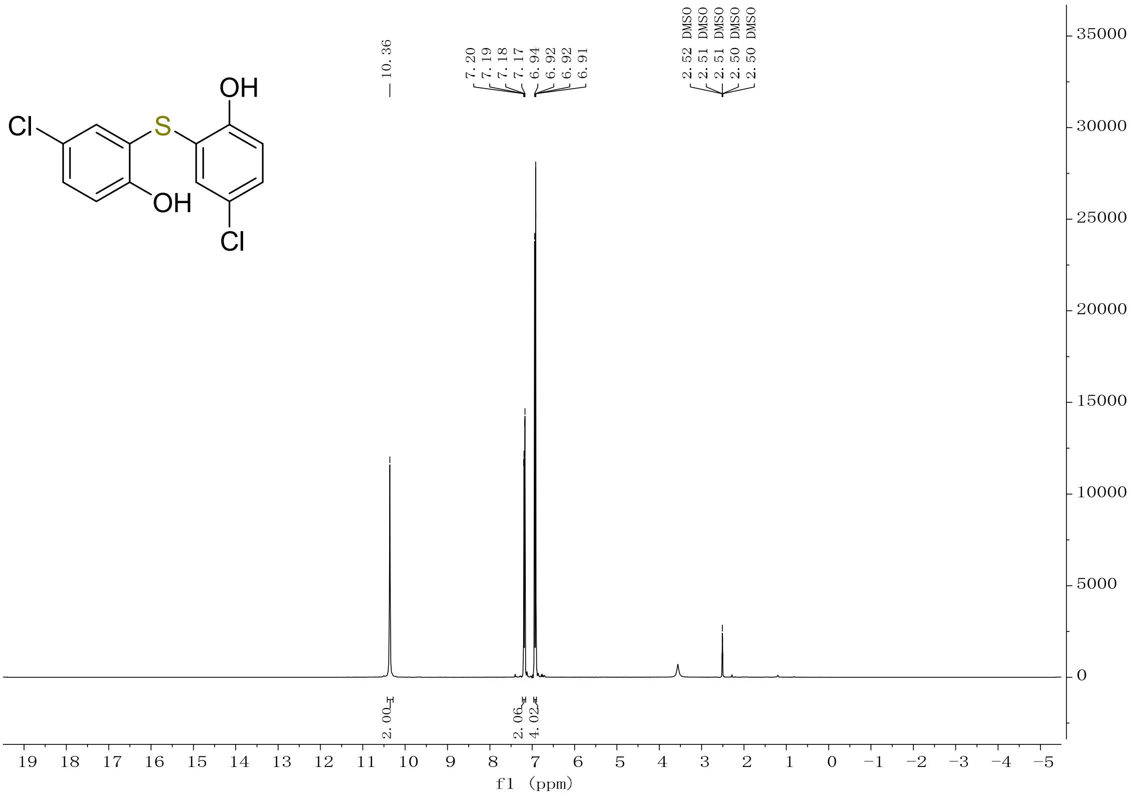

^{13}C NMR

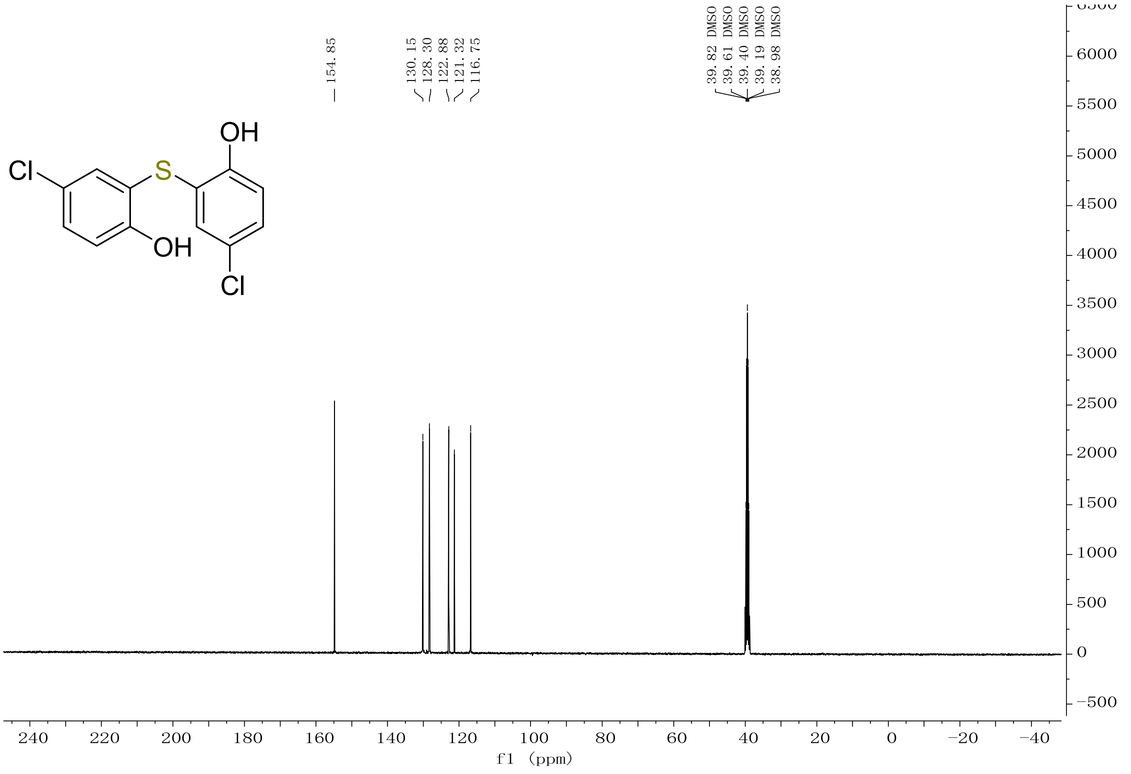

Di(1-naphthyl) sulfide（1t）

^{1}H NMR

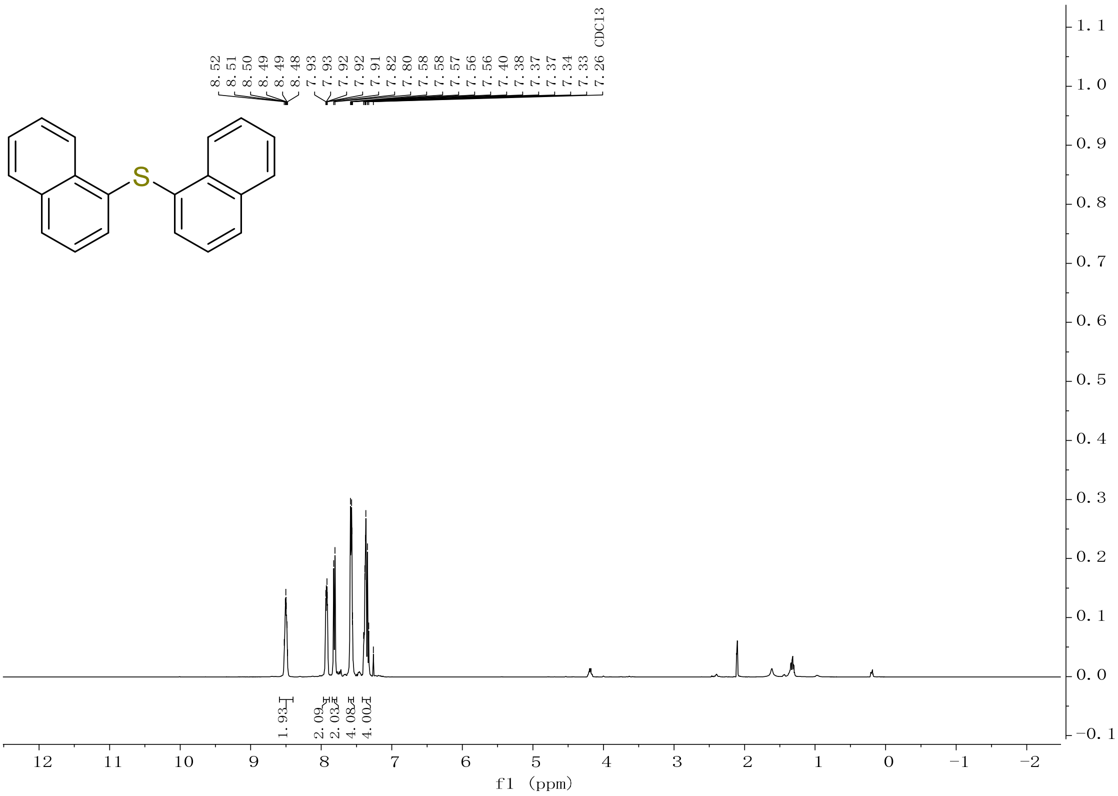

^{13}C NMR

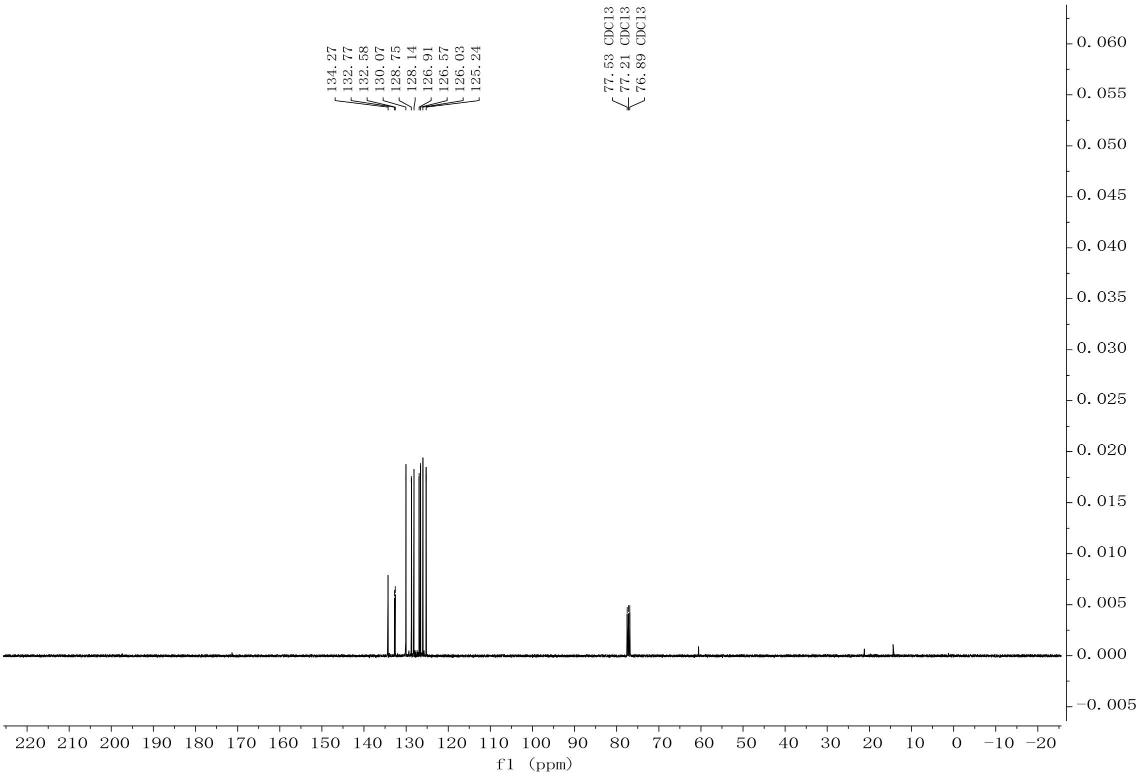

Dithienyl sulphide（1u）

^{1}H NMR

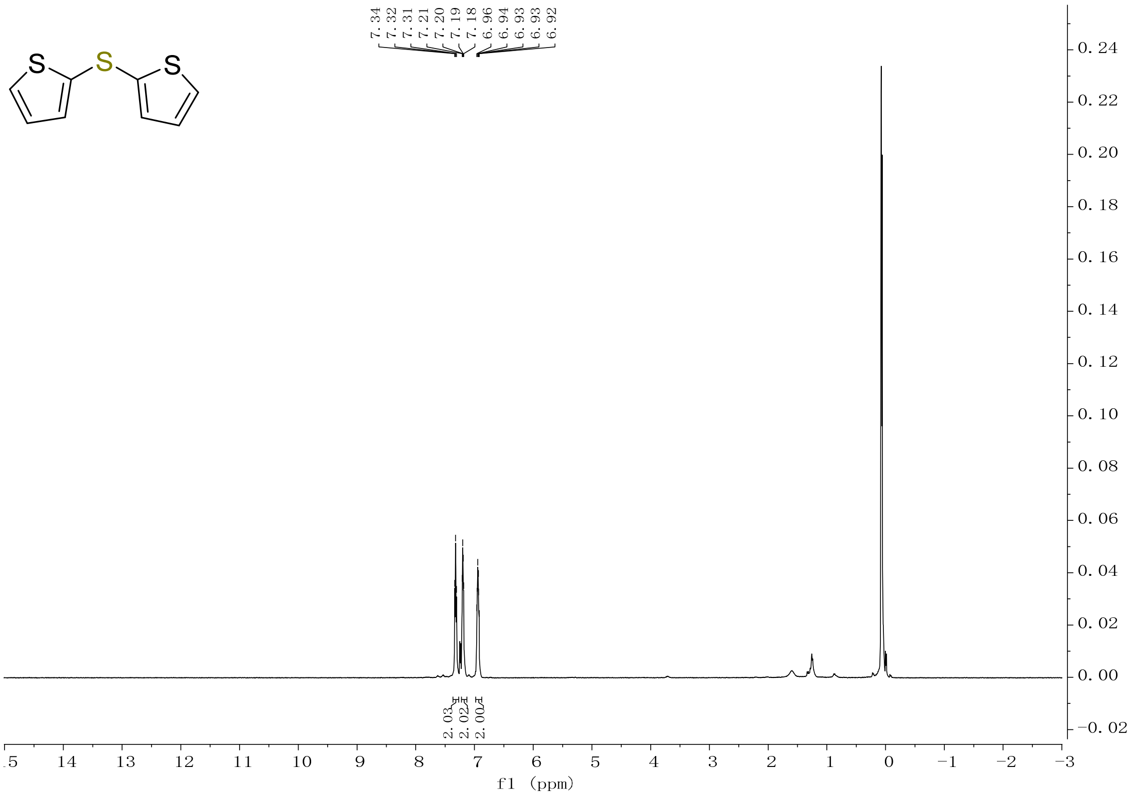

^{13}C NMR

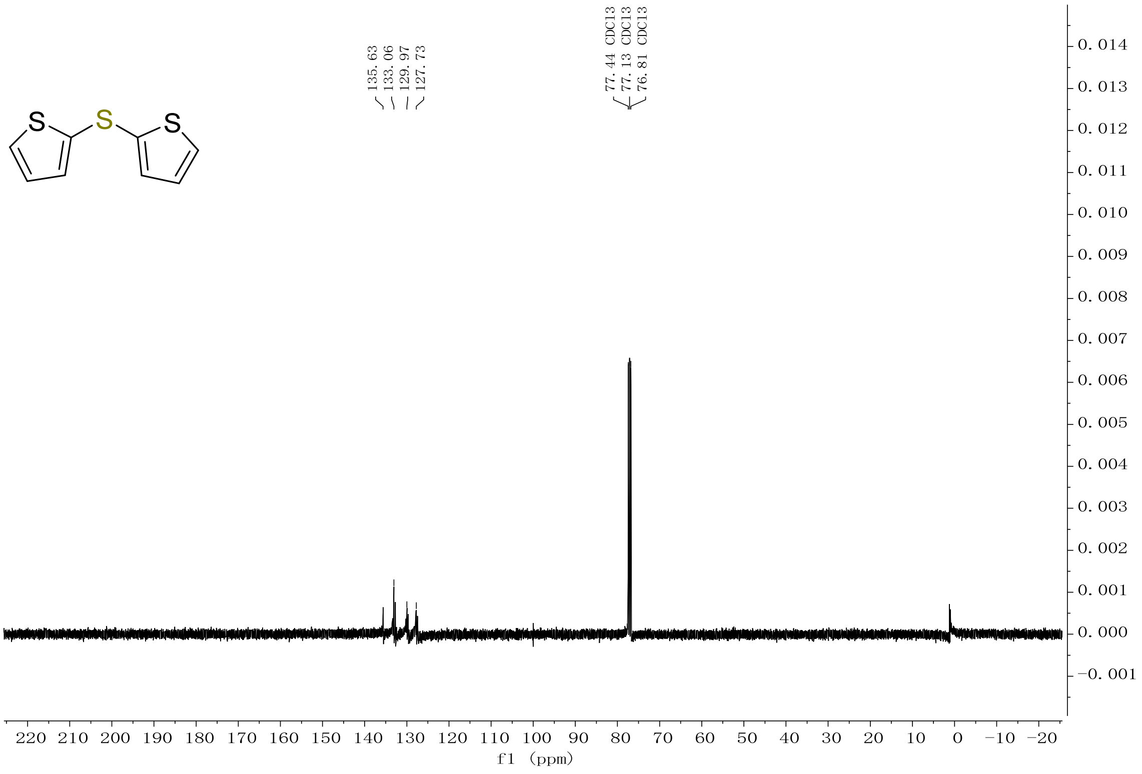

3-Thiophen-3-ylsulfanyl-thiophene[9]（1v）

^{1}H NMR

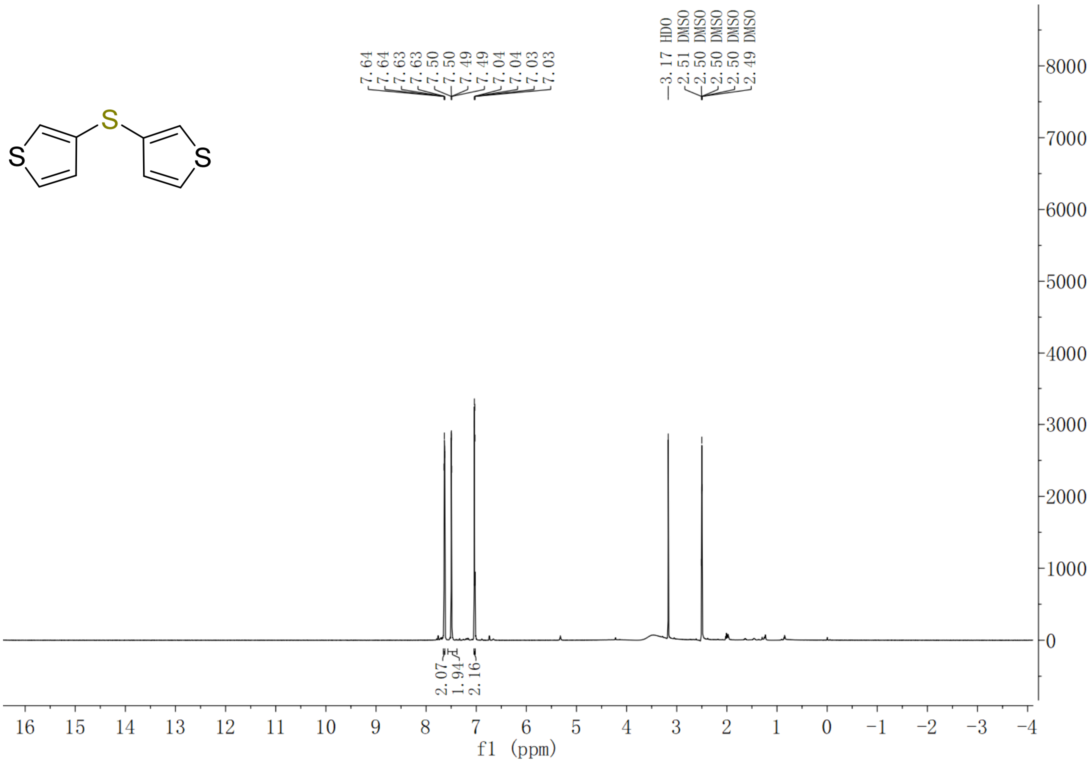

^{13}C NMR

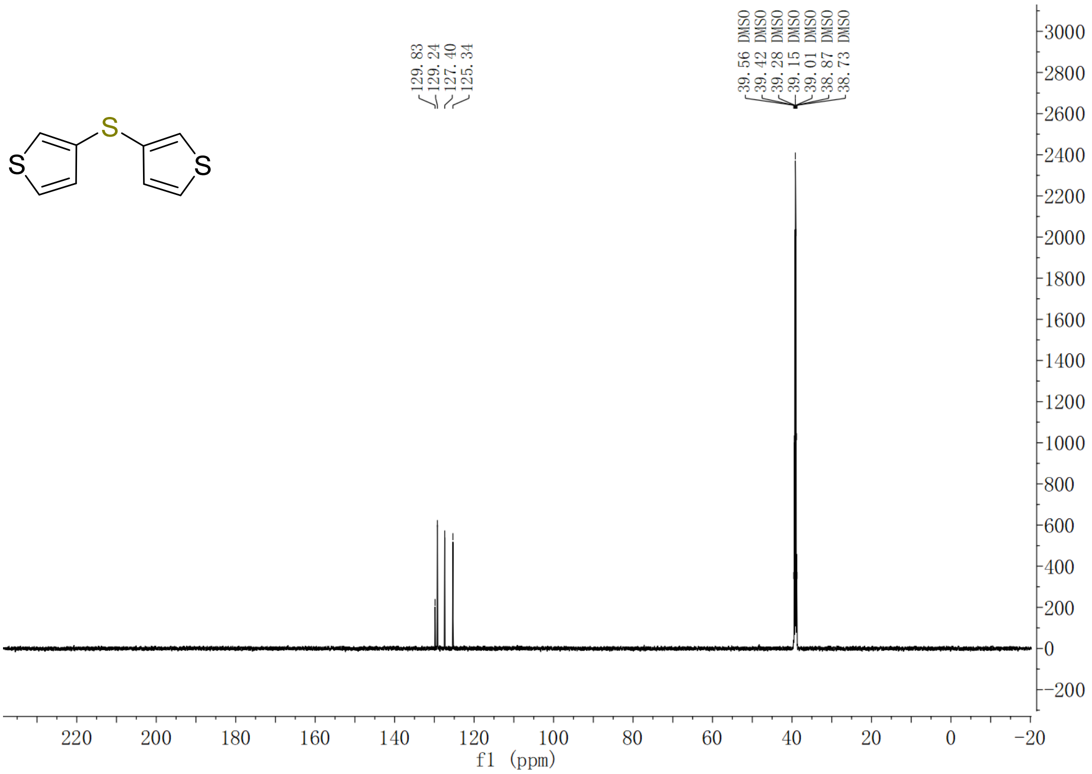

Di(2-pyridyl) sulfide（1w）

^{1}H NMR

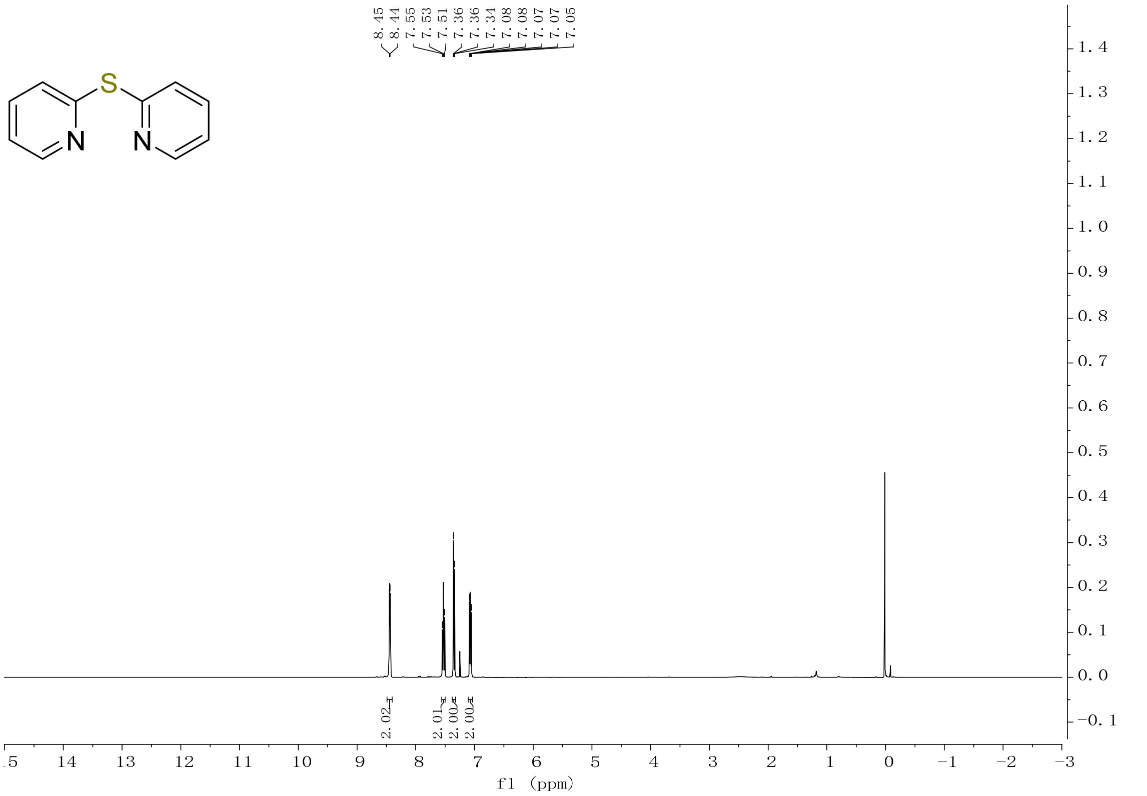

^{13}C NMR

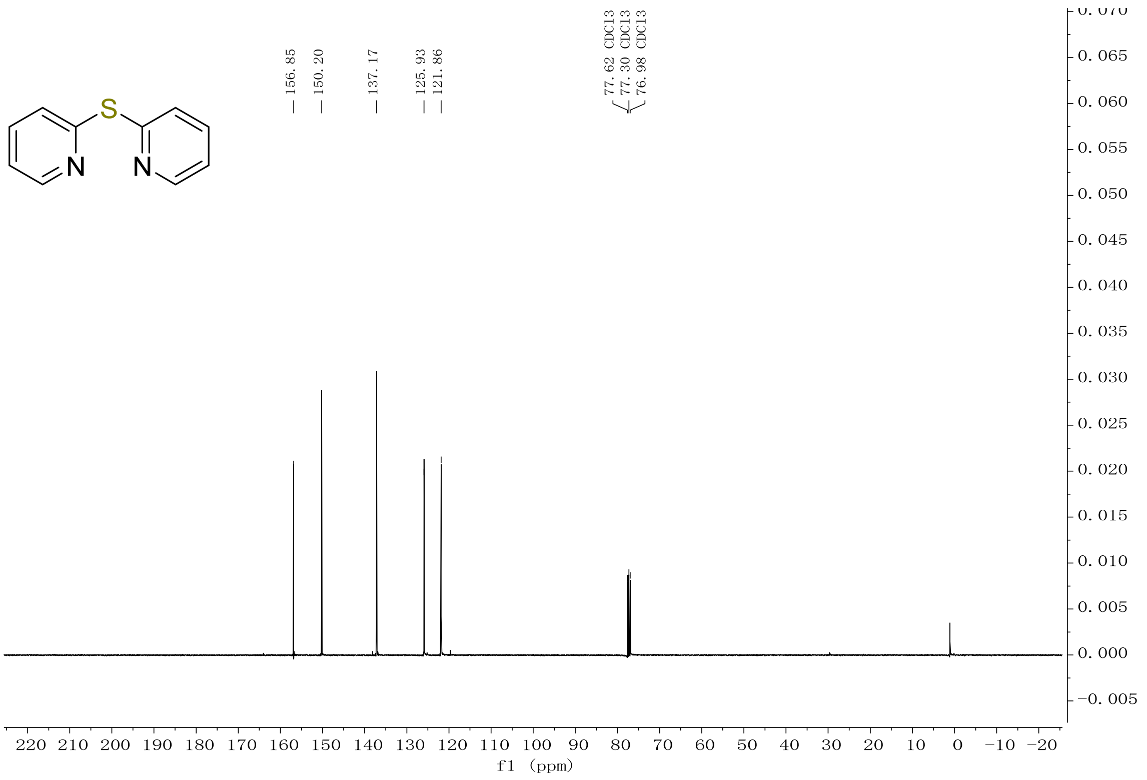

Di(3-pyridyl) sulfide（1x）

^{1}H NMR

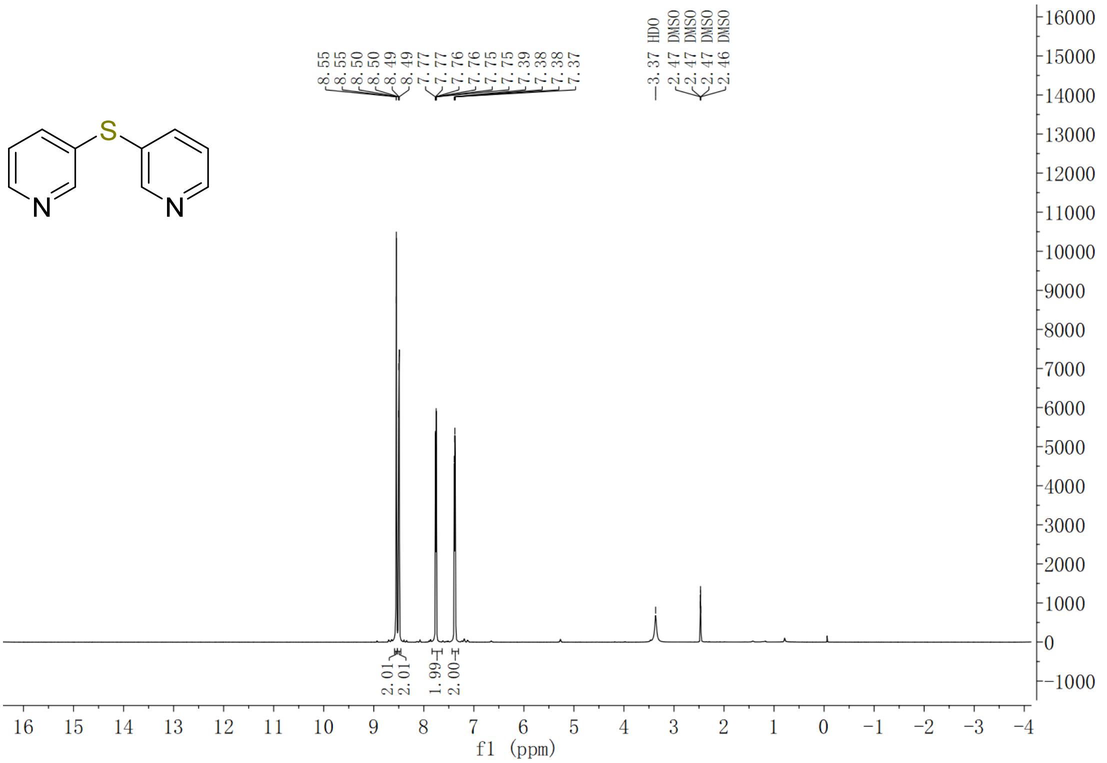

^{13}C NMR

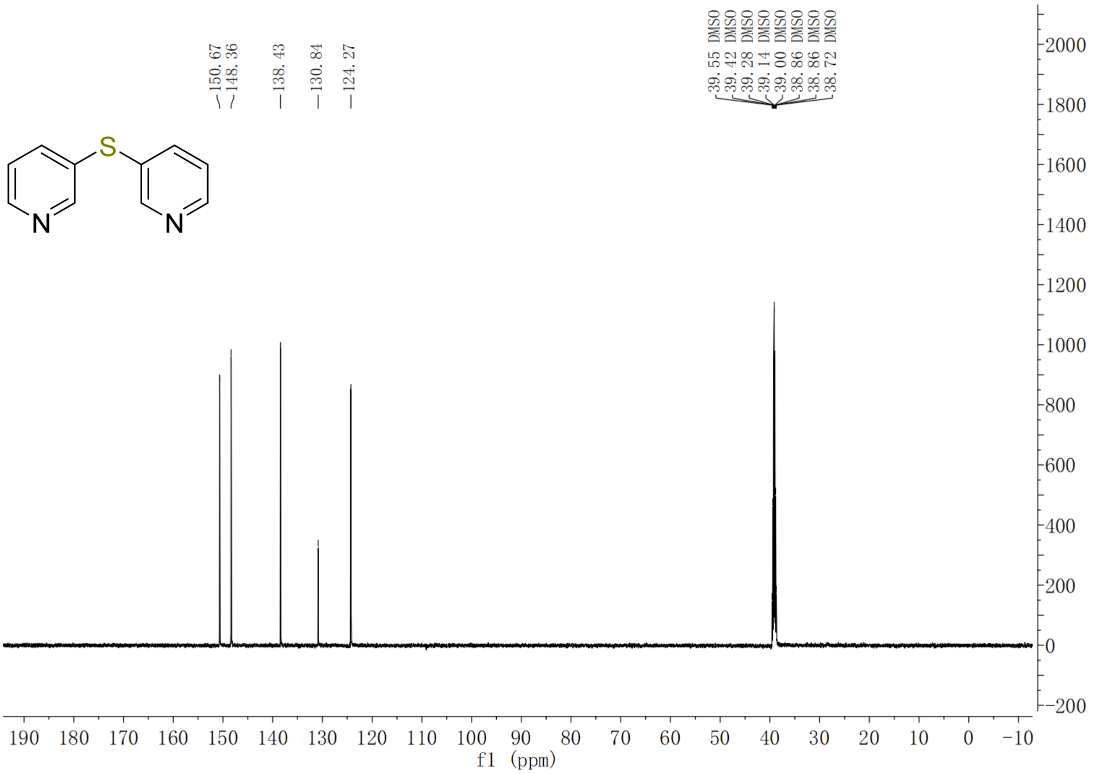

Di(4-pyridyl) sulfide（1y）

^{1}H NMR

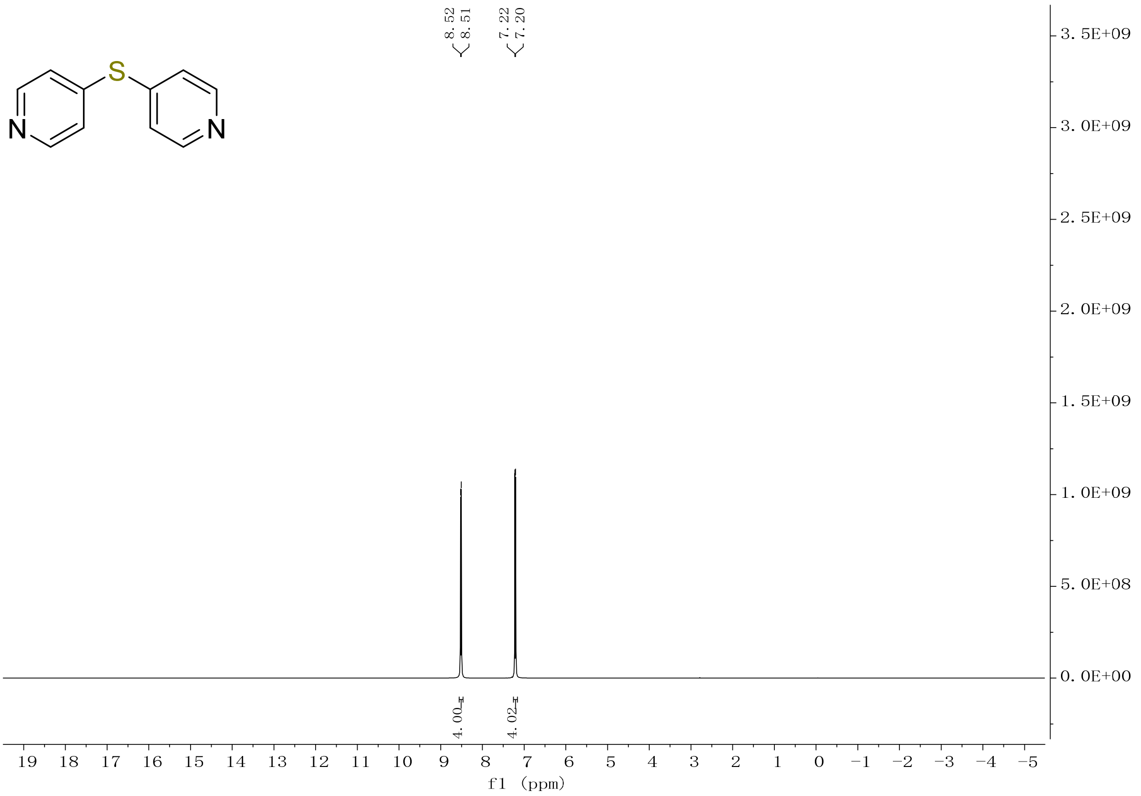

^{13}C NMR

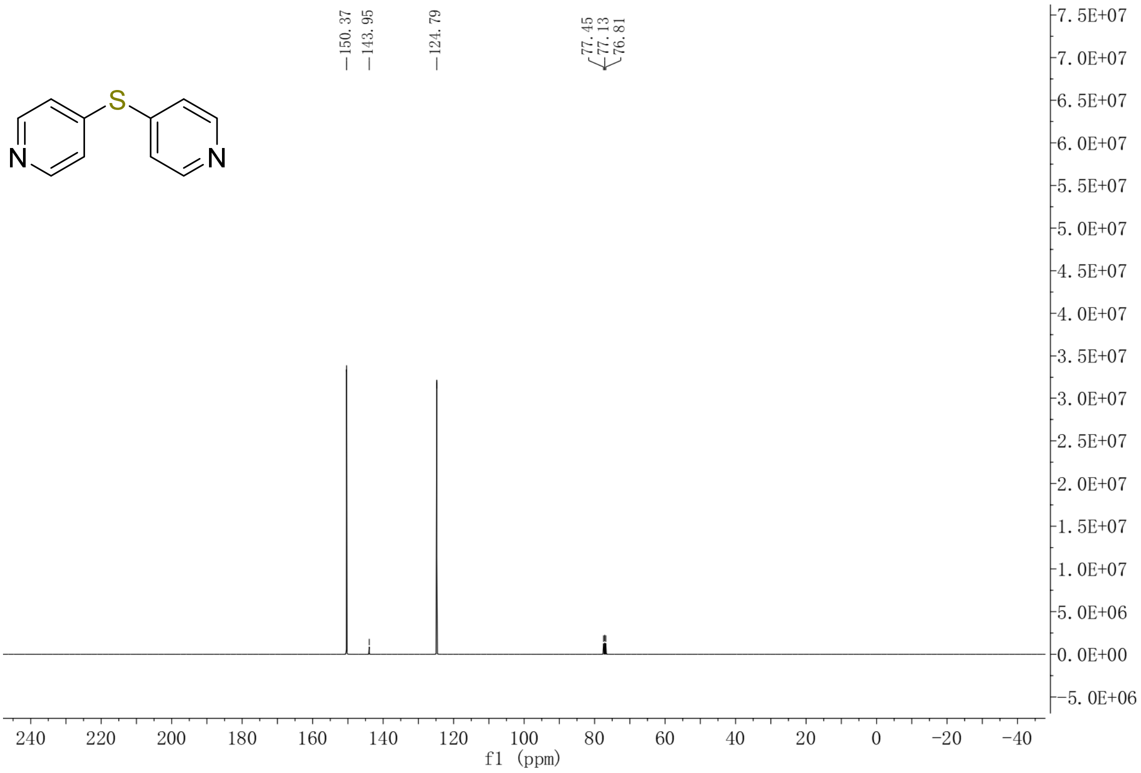

Supplement: Supplementary file 1 [file ijms-23-15360-s001.zip › ijms-2012366-supplementary.pdf]
